# Target Agnostic Photoaffinity Labelling by Sulfonylhydrazones

**DOI:** 10.1002/anie.202408701

**Published:** 2025-02-25

**Authors:** Kristóf Garami, Nikolett Péczka, László Petri, Tímea Imre, Tamás Langó, Zoltán Szabó, Zoltán Orgován, Pál Szabó, György Miklós Keserü, Péter Ábrányi‐Balogh

**Affiliations:** ^1^ Medicinal Chemistry Research Group HUN-REN Research Centre for Natural Sciences Magyar tudósok krt. 2 1117 Budapest Hungary; ^2^ National Drug Research and Development Laboratory HUN-REN Research Centre for Natural Sciences Magyar tudósok krt. 2 1117 Budapest, Hungary; ^3^ Department of Organic Chemistry and Technology Faculty of Chemical Technology and Biotechnology Budapest University of Technology and Economics Műegyetem rkp. 3. H-1111 Budapest Hungary; ^4^ MS Metabolomics Research Group HUN-REN Research Centre for Natural Sciences Magyar tudósok krt. 2 1117 Budapest Hungary; ^5^ Protein Bioinformatics Research Group HUN-REN Research Centre for Natural Sciences Magyar tudósok krt. 2 1117 Budapest Hungary; ^6^ Department of Medical Chemistry Albert Szent-Györgyi Medical School University of Szeged Dóm tér 8 H-6720 Szeged Hungary

**Keywords:** photoaffinity, sulfohydrazone, acetylcholine esterase, KRas, STAT

## Abstract

Photoaffinity labeling is a widely used methodology for interrogating small molecule‐protein interactions. However, these applications are limited by the few photo‐crosslinkers that typically modify the affinity and the binding mode of the original ligand. Here, we report the development of new target agnostic photoaffinity warheads, sulfohydrazones that form a reactive carbene upon UV irradiation. Careful optimization of the reaction conditions allowed us to effectively label five different amino acid residues in proteins. Our approach turned biologically relevant hydrazones and sulfohydrazones to intrinsically irreversible covalent binders without structural modifications by photoactivation as demonstrated on monoamine oxidase A (MAO‐A) enzyme and STAT5b (Signal transducer and activator of transcription 5b) transcription factor. Sulfohydrazones are readily accessible by transforming the corresponding carbonyl group of a ligand or a suitable tag that extends the application domain of the method for any ligands exemplified by conditional labelling of the acetylcholine esterase enzyme and the oncogenic mutant of GTP‐ase KRas^G12D^.

## Graphical abstract

Sulfonyl hydrazones were identified as new photo‐crosslinkers exploiting their base catalyzed low‐energy photoactivation that leads to reactive carbenes. The amino acid promiscuity, their usefulness in proteomics application and the range of labelled targets including enzymes, a PPI target and a transcription factor suggests that sulfonyl hydrazones might be a target agnostic alternative to current photoaffinity tags. Their synthetic availability and the option of late‐stage formation make them beneficial in both chemical biology and drug discovery applications.

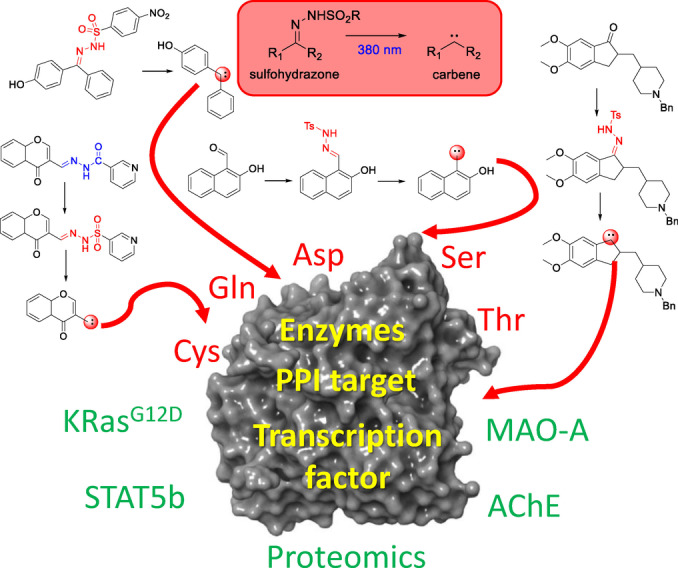

## Introduction

**Scheme 1:**
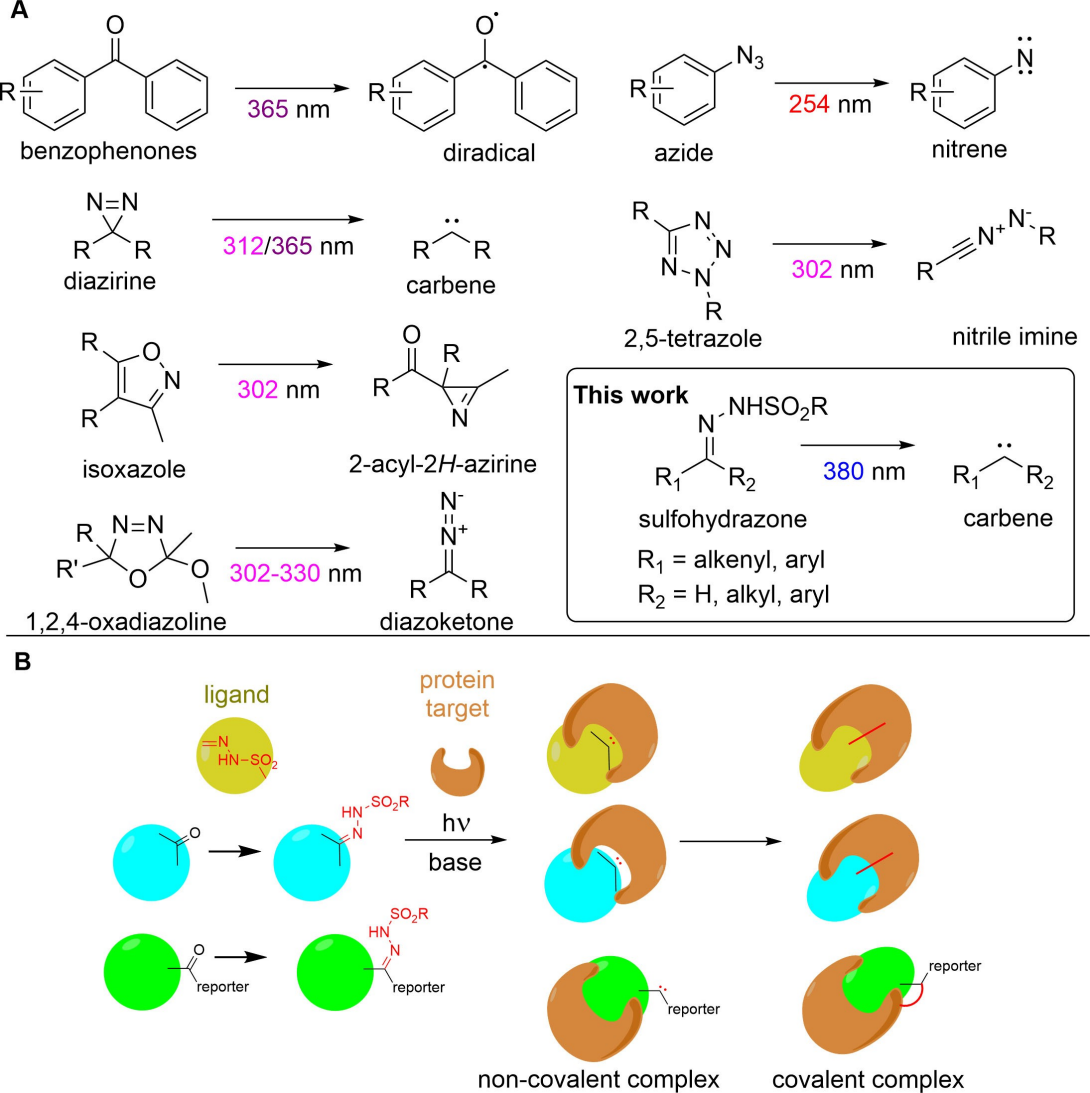
A) Collection of the most frequently used and the most recent photoaffinity tags and photo‐crosslinkers and their reaction upon photoactivation highlighting the activating wavelength by different colors. B) Protein labeling strategies with sulfonyl hydrazones: natively embedded sulfohydrazone moiety in the ligand (yellow), oxo group transformed to sulfohydrazone (cyan) and sulfohydrazone tag resulting in fully functionalized ligands (green).

Photoaffinity labeling is a powerful tool used frequently in both medicinal chemistry and chemical biology to investigate small molecule‐protein interactions.[^1^, ^2^, ^3^, ^4^, ^5^] Despite the rapid development of this general methodology, only a few photo‐crosslinkers producing carbenes, nitrenes or other radicals were described. The most frequently used warheads are aryl azides, benzophenones and diazirines (Scheme 1A). Due to their small size, highest activating wavelength and short irradiation time, diazirines are widely applied from fragment‐sized to drug‐like compounds, peptides, proteins and also in DNA‐encoded libraries.[^6^, ^7^, ^8^, ^9^, ^10^, ^11^, ^12^, ^13^, ^14^, ^15^, ^16^] More recently, four‐ and five‐membered rings were also introduced as photo‐crosslinkers, in particular tetrazoles, isoxazoles and 1,2,4‐oxadiazoline were shown to be efficient in photoaffinity labeling.[^17^, ^18^, ^19^, ^20^] All of these photo‐crosslinkers, however, need high energy activation (low wavelength: 254–330 nm for cyclic warheads) or long irradiation time at higher wavelengths (30 min for benzophenones at 365 nm) that might result in protein degradation.^[16]^ Furthermore, most of the known photoactive warheads are rarely involved in ligand binding pharmacophores and therefore the affinity of the ligand usually decreases by introducing the photoaffinity tags. Thus, stable photoactivable groups embedded in or formed easily from known ligands might be beneficial for direct photo‐cross‐linking.[^17^, ^21^, ^22^, ^23^, ^24^, ^25^]

Synthetic organic chemistry is a rich source of biocompatible and biorthogonal reactions that earned numerous applications in chemical biology.[^26^, ^27^, ^28^, ^29^, ^30^, ^31^] Searching for a photoreactive functional group in the organic chemistry toolbox that is readily available in bioactive compounds,^[32]^ we identified sulfohydrazones being the validated precursors of carbenes.[^33^, ^34^, ^35^, ^36^, ^37^, ^38^, ^39^, ^40^]

**Figure 1:**
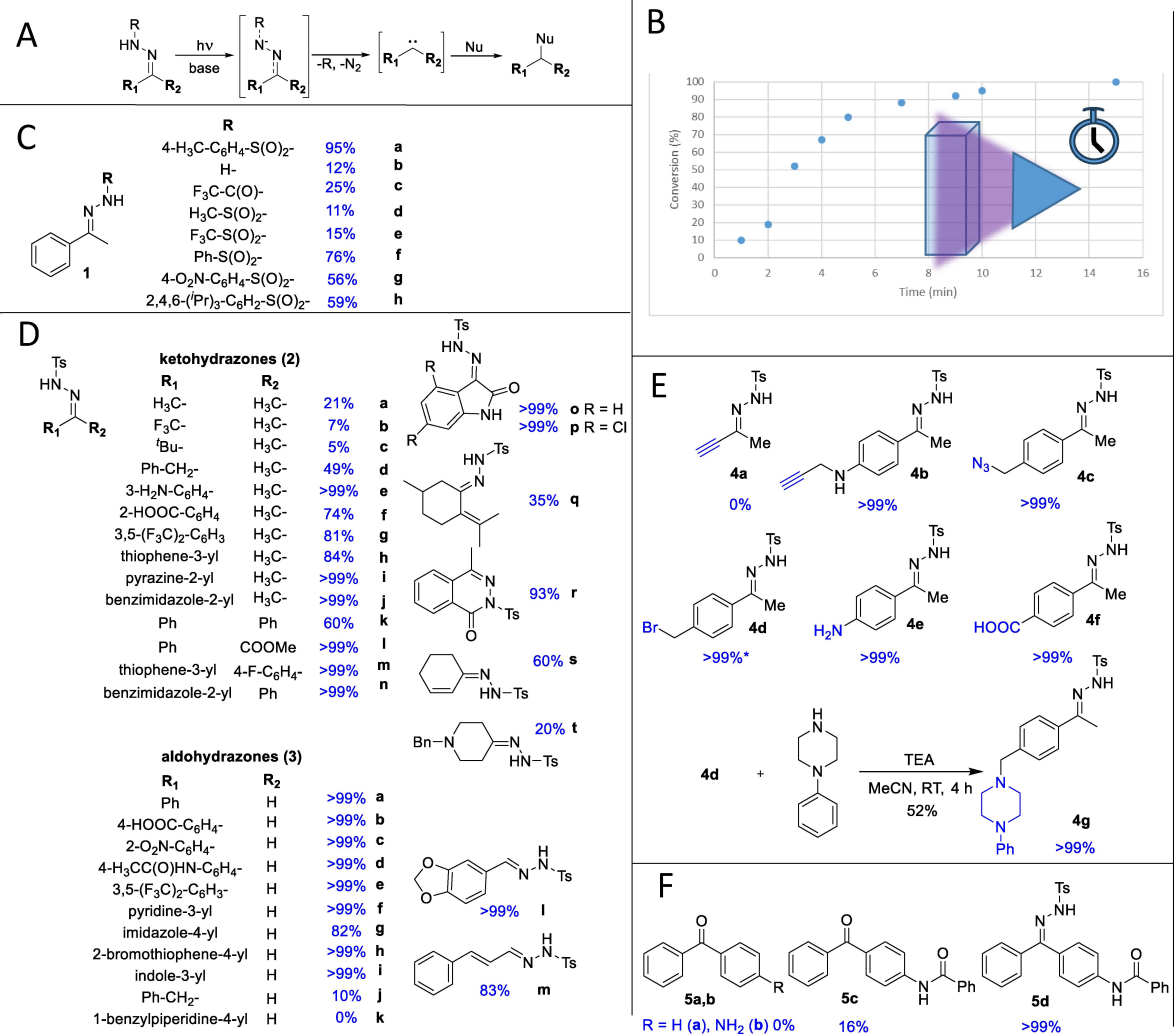
A) General reaction scheme from the base and photocatalyzed Bamford‐Stevens reaction. Nu: nucleophile e.g thiol/thiolate, alcohol, water, amine, carboxylate. B) Conversion of tosylhydrazone **1** **a** in time at 380 nm irradiation in PBS pH 7.4. C) Scope of acetophenone hydrazones. Blue percentage indicate conversion of the hydrazone after photoactivation. Irradiations were performed at 380 nm in PBS pH 7.4 for 10 min. D) Scope of ketohydrazones and aldohydrazones after 10 min irradiation at 380 nm in PBS pH 7.4. Blue percentage indicate conversion of the hydrazone after photoactivation. E. Conversion of the tosylhydrazone tags after 10 min irradiation at 380 nm in PBS pH 7.4. Blue percentage indicate conversion of the hydrazone after photoactivation. * During the full conversion of **4** **d** rapid substitution of the Br to OH was also detected. F. Conversion of benzophenone and derivatives compared to the corresponding tosylhydrazone after 10 min irradiation at 380 nm in PBS pH 7.4. Blue percentage indicate conversion of the probe after photoactivation.

We envisaged that carbenes formed upon the base catalyzed photoactivation of sulfohydrazones (Figure 1A) might enable photo‐cross‐linking of diverse probe ligands. The most trivial way is that the ligand has a sulfohydrazone moiety. Alternatively, oxo groups available in the probe can be transformed easily to sulfohydrazones. Finally, the sulfohydrazone moiety can be coupled as a photoaffinity tag (Scheme 1B).[^33^, ^37^, ^41^, ^42^]

## Results and Discussion

First, we synthesized the tosylhydrazone of acetophenone (**1** **a**), and investigated the impact of the wavelength on its photoactivation in the presence of unreactive indoprofen as internal standard.[^43^, ^44^] Measuring the depletion of **1** **a** as a surrogate of the reactive carbene we reached full conversion after 15 min irradiation at 360 nm and 380 nm. Irradiation at 405 nm resulted in 27 % conversion, while no photoactivation was observed above 440 nm. We choose 380 nm as optimum that is more advantageous for biology applications than the wavelengths (312 nm, 320 nm and 360 nm) typically needed for photoaffinity labelling.^[16]^ Second, we investigated the effect of the irradiation time that showed 10 minutes as optimum (Figure 1B). Third, we turned our attention to the buffers generally used in protein labeling experiments.[^45^, ^46^] In TRIS (*tris*(hydroxymethyl)aminomethane) buffer the conversion at pH 7.4, 7.9 and 8.4 was 3 %, 86 % and 98 %, respectively, while that for PBS (phosphate buffered saline) buffer at the same pH values was 82 %, 94 % and 95 %, respectively. The Bamford‐Stevens reaction requires a base for the deprotonation of the hydrazone that is in line with the basicity of the buffer components i.e. p*K*_a_ (*tris*‐(hydroxymethyl)aminomethane)=8.07, p*K*_a_ (HPO_4_^2−^)=7.21.

Monitoring the scope of the photocatalyzed carbene formation reaction, a set of hydrazones and tosylhydrazones were synthesized from acetophenone and their reactivity was investigated in PBS buffer (pH 7.4) using 380 nm irradiation for 10 min (Figure 1C, for the details of syntheses see Supporting Information). We have found that acetophenone hydrazone (**1** **b**) and the trifluoroacetyl derivative (**1** **c**) were slightly activated. Methanesulfonyl (**1** **d**) and trifluoromethanesulfonyl (**1** **e**) substituents provided compounds with limited reactivity, while the conversion increased significantly with phenylsulfonyl (**1** **f**), but did not reach the full conversion as **1** **a**. Nitro (**1** **g**) or isopropyl (**1** **h**) substituents on the phenyl ring decreased further the reactivity of the sulfohydrazones. The high reactivity and the appropriate aqueous stability (>4 h at pH 7.4, 7.9 and 8.4) suggested tosylhydrazone as an ideal carbene source in photoaffinity based applications.

We also investigated the photoactivation of **1** **a** in the presence of glutathione (GSH). The small fragment reacted with GSH, and we checked the labeling site on GSH by LC‐MS/MS (liquid chromatography coupled tandem mass spectrometry) measurements. Based on the total ion chromatogram and the fragmentation pattern we concluded that >90 % of labelling occurred at cysteine while a small amount of labelled terminal amino‐ and carboxyl groups were observed (Supplementary Figure S1).

The scope and limitations of the photoactivation was investigated with a large number of diverse tosylhydrazones prepared from ketones (**2** **a**–**t**) and aldehydes (**3** **a**–**m**) (Figure 1D, for the details of synthesis see Supporting Information) in PBS buffer at pH 7.4. We observed that having only alkyl or benzyl substituents (**2** **a**–**d**, **2** **t**, **3** **j**, **3** **k**) blocked the reaction significantly, but in the presence of at least one carbocyclic or heterocyclic aromatic ring (**2** **e**–**p**, **2** **r**, **3** **a**–**i**, **3** **l**) or an olefinic double bond (**2** **q**, **2** **s**, **3** **m**) the photoactivation occurs that can be explained by the carbene‐stabilizing effect of conjugated systems.^[47]^ Introducing further aromatic rings (**2** **k**), however, decreased the conversion presumably by steric hindrance. Focusing on the functional group tolerance of the reaction, several functional groups [COOMe (**2** **l**), COOH (**2** **f**, **3** **b**), NH_2_ (**2** **e**), NO_2_ (**3** **c**), CF_3_ (**2** **g**, **3** **e**), Br (**3** **h**), F (**2** **m**), amides (**3** **d**)] were tolerated, and endocyclic tosylhydrazone (**2** **r**) reacted, as well.

Next, we have synthesized a set of tosylhydrazones that could be used as labeling tags equipped with acetylene (**4** **a**, **b**), azide (**4** **c**), bromo (**4** **d**), further amino (**4** **e**) and carboxyl (**4** **f**) groups ready for coupling to protein ligands (Figure 1E, for the details of synthesis see Supporting Information). These were tested in the model assay and were found to be appropriately reactive. During the aqueous treatment **4** **d** reacted with water, so we detected the photoactivation of the hydroxymethyl derivative with full conversion. Next, as an example, we alkylated phenylpiperazine with the bromobenzyl tag (**4** **d**) resulting in derivative **4** **g** and confirmed its reactivity (Figure 1E).

We have compared the photoactivation of tosylhydrazones with the corresponding benzophenone photoaffinity tags at 380 nm irradiation for 10 min in PBS 7.4 buffer (Figure 1F). Tosylhydrazone **1** **a** showed 95 % conversion compared to benzophenone (**5** **a**) that did not react at all. Similarly, 4‐aminobenzophenone (**5** **b**) did not react compared to the 4‐amino tosylhydrazone (**4** **d**) showing full conversion. The benzamido benzophenone derivative (**5** **c**), however, was slightly reactive (16 % conversion in 10 min), but still did not reach the reactivity of the corresponding tosylhydrazone (**5** **d**, full conversion in 10 min).

**Figure 2:**
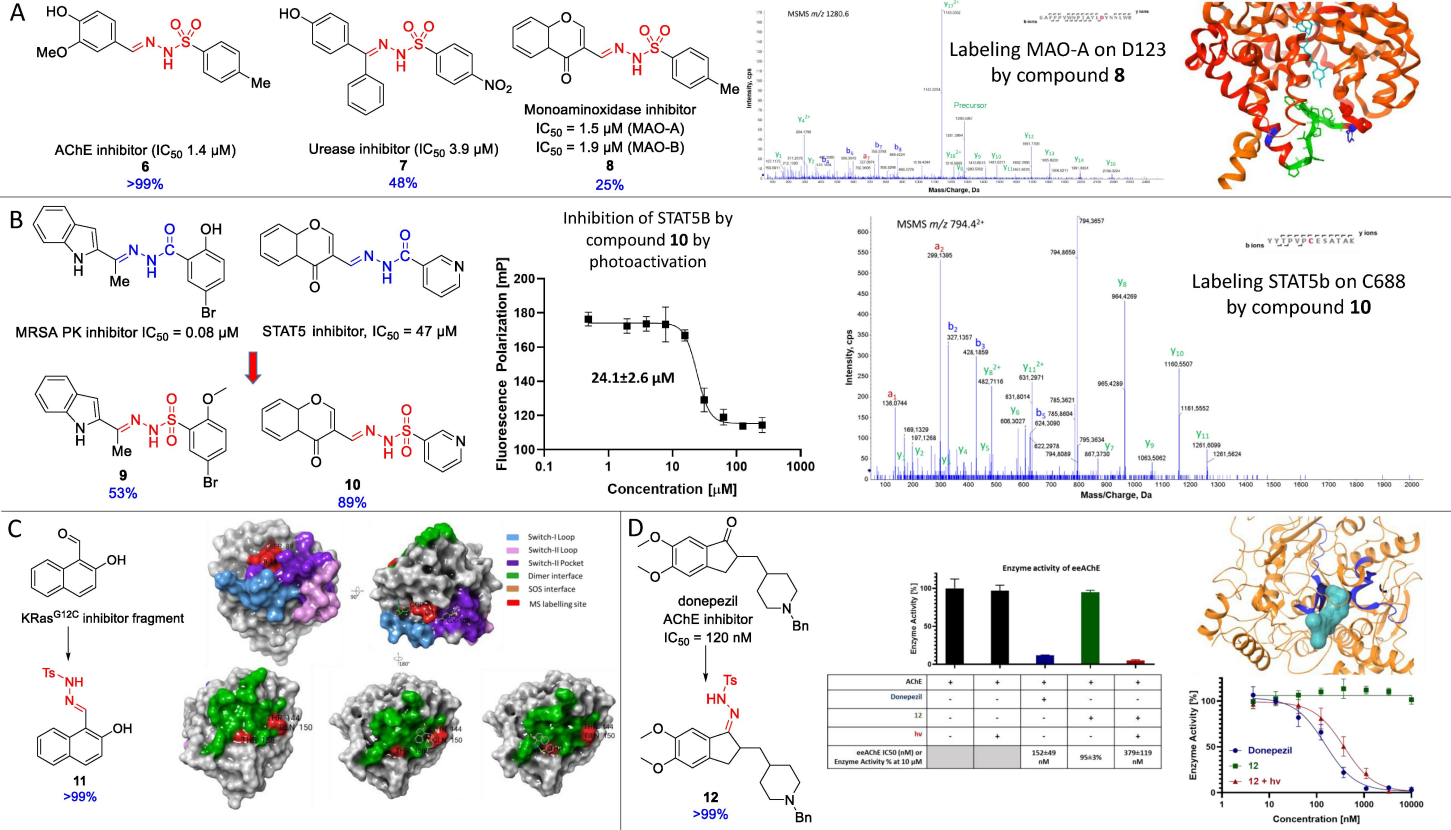
A) Bioactive sulfonyl hydrazone inhibitors against AChE (**6**), urease (**7**) and MAOs (**8**). MSMS spectrum proving the labelling of compound **8** on D123. Crystal structure of MAO‐A (PBD: 1O5 W) highlighting cofactor FAD (flavin adenine dinucleotide) by cyan, G110‐loop by green and D123 by dark blue. Blue percentage values refer for the conversion of the tosylhydrazones after 10 min irradiation in PBS pH 7.4. B) Sulfohydrazone derivatives (**9** and **10**) of bioactive carbohydrazones. Blue percentage values refer for the conversion of the tosylhydrazones after 10 min irradiation PBS pH 7.4. STAT5b inhibition assay curve (*n*=3) showing IC_50_ with irradiation. MSMS spectrum proving the labeling of compound **10** on STAT5b C688. C) KRas^G12C^ fragment and the tosylhydrazone derivative **11**. Modelling of binding sites based on tryptic digestion and MSMS (PDB: 7RPZ). Blue percentage values refer for the conversion of the tosylhydrazone after 10 min irradiation in PBS pH 7.4. D) Donepezil and donepezil‐tosylhydrazone (**12**). Results of the biochemical enzyme inhibition assay (*n*=3) with control experiments. Blue percentage values refer for the conversion of the tosylhydrazone after 10 min irradiation in PBS pH 7.4. Crystal structure of AChE with donepezil (PDB: 4EY7) highlighting the labelled amino acid sequences and binding site surfaces by blue and donepezil by cyan.

In order to show intrinsic reactivity of known ligands with embedded sulfohydrazone moiety we have synthesized acetylcholine esterase (AChE),^[48]^ urease,^[49]^ monoamine oxidase^[50]^ targeting molecules (Figure 2A, for the details of synthesis see Supporting Information). We proved that AChE inhibitor **6** can be fully activated, while urease inhibitor **7** was cleaved partially potentially due to steric hindrance. Non‐competitive MAO‐A inhibitor **8** also showed somewhat lower activation possibly due to the neighboring chromone core. However, we have successfully turned this non‐covalent compound into a covalent binder. A close derivative of inhibitor **8** was reported to bind to the G110 loop near the C‐terminal transmembrane helix of MAO‐A (PDB: 1O5 W) interacting non‐covalently with Y116, F104, P105 and Y113.^[50]^ This loop has fundamental role in controlling substrate access to the binding site and was identified as a potential allosteric site.^[51]^ Here, we have proven by tryptic digestion and LC‐MS/MS that compound **8** labeled 8 % of MAO‐A covalently on Y106 and D123 those are in close proximity to the predicted allosteric binding site (Figure 2A, Supplementary Figure S2). The labeling efficiency at specific sites of the target protein were determined by comparing the total intensity of all modified peptides to the intensity of all unmodified peptides identified during the LC‐MS peptide mapping.

Next, we have synthesized the sulfohydrazone analogues of embedded hydrazone ligands of MRSA protein kinase^[52]^ and the transcription factor STAT5b[^53^, ^54^] (Figure 2B, for the details of synthesis see Supporting Information). Inhibitor derivative **9** reacted partially, while non‐covalent STAT5b inhibitor **10** showed high conversion. Sulfohydrazone **10** showed no inhibitory activity on STAT5B under 250 μM, however after irradiation the IC_50_ increased to 24.1 μM. Covalent labeling was proven by tryptic digestion and LC‐MS/MS on C279 and C688. Labelling efficiency (25 % and 52 %, respectively) was determined by comparing the intensities of the probe‐ and the iodoacetamide labelled cysteines (Supplementary Figure S3). C279 is part of the coiled‐coil domain, and its labeling on the analogue protein STAT3 inhibits phosphorylation, dimerization, nuclear translocation, and transcriptional activity.^[55]^ C688 can be found on the C‐terminal end of the SH2 domain close to the highly conserved phosphorylation site (Y699). Binding closely to that tyrosine might inhibit dimerization and entering STAT5b to the nucleus and transcription.^[54]^

Extending the methodology to ligands having aldehyde or keto functions we synthesized the tosylhydrazone analogues of an aldehyde type fragment inhibitor of KRas^G12C[56]^. and anti‐Alzheimer drug donepezil^[57]^ (Figure 2C and 2D, for the details of synthesis see Supporting Information).

Ruling out trivial labelling of the most accessible C12, we have chosen oncogenic KRas^G12D^ and labeled by photoactivation with compound **11**. In this case, intact MS showed single and multiple labelling, in total 27 % of the protein. The binding sites were identified by tryptic digestion and LC‐MS/MS (Supplementary Figure S4). KRas participates in several protein‐protein interactions (PPI) having role in cancer, thus mapping its surface is an important research field.^[58]^ Inhibitor **11** labeled S89 and D12 on the Switch‐II pocket near the GDP binding site and amino acids on the dimerization interface (T144, T156, Q150) that we visualized on Figure 2C.

In case of AChE we showed that donepezil tosylhydrazone (**12**) did not inhibit the activity of the enzyme, however photoactivation led to significant enzyme inhibition (Figure 2D, IC_50_=379 nM). Covalent binding of **12** was confirmed by mass spectrometry. Enzymatic digestions of the labeled enzyme (Supplementary Figure S5) showed labeling at the donepezil binding site (sequences W^473^MGVIH^478^ GYEIE^452^ and LNVWVPATPRP^145^HNLTVMVWIY^155^ GGGF^159^, Figure 2D).[^57^, ^58^] Detected peptides originated from these sites (modified or unmodified) by Glu‐C or chymotrypsin digestion are listed in Supporting Information Table S5. The labeling was approximately 99 % for the 452–473 sequence and 75 % for the 135–159 sequence.

**Figure 3:**
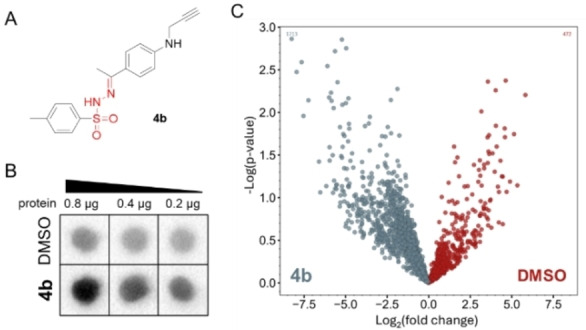
A) Chemical structure of the probe applied in proteomic labeling experiments; B) Dot blot analysis of biotinylation rate in HEK293 lysates treated with generic fragment probe (**4** **b**). The blot confirms the increased biotinylation rate after **4** **b** probe treatment; C) Volcano plot depiction of MS‐identified protein labelling effectiveness in HEK293 cell lysates achieved by probe **4** **b** compared to the DMSO‐treated control.

It should be noted that in the above examples we have proven the labeling of Cys, Asp, Ser, Thr and interestingly Gln available in proteins that suggests tosylhydrazones being a powerful photoactivable group in chemoproteomics studies. To further demonstrate their usefulness in complex biological systems we used **4** **b** as a generic small fragment probe in cell lysates. For the enrichment of the labelled proteins we synthesized azido‐PEG3‐SS‐biotin (see Supporting Information) that enabled biotinylation by click reaction and we developed a two‐step enrichment protocol exploiting the presence of biotin and the specific cleavage of the disulfide bond in the linker. HEK293 cell lysates were treated with the **4** **b** probe (Figure 3A), and following biotin conjugation via click chemistry, dot blot analysis confirmed the increased biotinylation of the **4** **b** treated lysates as compared to the control samples (Figure 3B). In addition, mass spectrometry (MS) analysis revealed the enrichment of a large number of labeled proteins compared to the DMSO‐treated control samples (Figure 3C, Supporting Information Table S1).

## Conclusion

We found that sulfonyl hydrazones can be considered as new photo‐crosslinkers exploiting their base catalyzed photoactivation that leads to reactive carbenes. Due to their low‐energy activation, they can avoid target degradation upon irradiation. Investigating a large number of sulfonyl hydrazones prepared from a range of aromatic or conjugated ketones and aldehydes, their photoactivation showed high functional group tolerance and a wide substrate specificity enabling the formation of the photo‐crosslinker from diverse structural moieties. We have shown on monoamine oxidase A and on STAT5b transcription factor that pharmacophoric sulfohydrazones embedded in the ligand can be used as photoactivable warheads turning non‐covalent inhibitors to irreversible binders without influencing their binding properties. Furthermore, our experiments with acetylcholine esterase and KRas^G12D^ inhibitors demonstrated that bioactive ligands can be caged by transforming their oxo functional group to tosylhydrazone. Photoactivation of these tosylhydrazones led to the covalent labeling of their protein target on a range of amino acids. In addition, a sulfohydrazone fragment probe was successfully applied in a chemoproteomics study showing concentration dependent enrichment of the labelled proteins. The amino acid promiscuity and the presented range of targets including enzymes, a PPI target and a transcription factor together with the different applications and the low‐energy activation at 380 nm suggests that sulfohydrazone photo‐crosslinkers might be a beneficial target agnostic alternative to other photoaffinity tags in both chemical biology and medicinal chemistry settings.

## Supporting Information

Supporting Information contains synthetic procedures, compound characterization, biochemical assays, MSMS data, protein labeling protocols and proteomics.

## Conflict of Interests

The authors declare no conflict of interest.

1

## Supporting information

As a service to our authors and readers, this journal provides supporting information supplied by the authors. Such materials are peer reviewed and may be re‐organized for online delivery, but are not copy‐edited or typeset. Technical support issues arising from supporting information (other than missing files) should be addressed to the authors.

Supporting Information

Supporting Information

## Data Availability

The data that support the findings of this study are available in the supplementary material of this article.
