# Supplementary material for: Target Agnostic Photoaffinity Labelling by Sulfonylhydrazones
Source: Angew Chem Int Ed Engl. 2025 Feb 25;64(17):e202408701. doi: 10.1002/anie.202408701 (PMC12015381; doi:10.1002/anie.202408701)

Angewandte Chemie
Eine Zeitschrift der Gesellschaft Deutscher Chemiker

Supporting Information

Target Agnostic Photoaffinity Labelling by Sulfonylhydrazones

K. Garami, N. Péczka, L. Petri, T. Imre, T. Langó, Z. Szabó, Z. Orgován, P. Szabó, G. M. Keserü, P. Ábrányi-Balogh**

Supporting Information

Target agnostic photoaffinity labelling by tosylhydrazones

Kristóf Garami,[a,b,c] Nikolett Péczka[a,b,c], László Petri[a,b], Tímea Imre,[a,b,d] Tamás Langó[e], Zoltán Szabó[f], Zoltán Orgován,[a,b] Pál Szabó,[b,d] György Miklós Keserű[a,b,c]* and Péter Ábrányi-Balogh*[a,b,c]

[a] K.G., L. P., N. P., Z.O., T.I., P. Á.-B and G. M. K.

Medicinal Chemistry Research Group, Research Centre for Natural Sciences, Magyar tudósok krt. 2, 1117 Budapest, Hungary

E-mail: keseru.gyorgy@ttk.hu; abranyi-balogh.peter@ttk.hu

[b] K.G., L. P., N. P., Z.O., T.I., P. Á.-B. and G. M. K.

National Drug Research and Development Laboratory, Research Centre for Natural Sciences, Magyar tudósok krt. 2, 1117 Budapest, Hungary

[c] K. G., N. P., P. Á.-B. and G. M. K.

Department of Organic Chemistry and Technology, Faculty of Chemical Technology and Biotechnology, Budapest University of Technology and Economics, Műegyetem rkp. 3., H-1111 Budapest, Hungary

[d] T.I., P. Sz.

MS Metabolomics Research Group, Research Centre for Natural Sciences, Magyar tudósok krt. 2, 1117 Budapest, Hungary

[e] T. L.

Protein Bioinformatics Research Group

HUN-REN Research Centre for Natural Sciences

Magyar tudósok krt. 2, 1117 Budapest, Hungary

[f] Z. Sz.

Department of Medical Chemistry

Albert Szent-Györgyi Medical School, University of Szeged

Dóm tér 8, H-6720 Szeged, Hungary

Table of Contents

Figure S1. GSH labeling by compound **1a** 3
Experimental conditions of LC-MS measurements of GSH labeling 3
Figure S2: MAO-A labeling by compound **8** 4
Figure S3: STAT5B labeling by compound **10** 6
Figure S4: $KRas^{G12D}$ labeling by compound **11** 8
Figure S5: AChE labeling by compound **12** 9
Table S1. Detailed results of chemoproteomic analysis on HEK293 lysates 9
Experimental conditions of sulfohydrazone photoactivation assay 9
Experimental conditions of LC-MS measurements of intact protein adducts 9
MAO-A labeling protocol 10
STAT5b labeling protocol 10
STAT5b biochemical assay 10
$KRas^{G12D}$ labeling protocol 11
Induced fit docking 11
AChE biochemical assay 11
AChE labeling protocol 12
Cell culture and lysate production for chemoproteomics 13
HEK293 lysate labelling for chemoproteomics 14
Dot blot analysis of HEK293 lysates 15
LC-MS/MS analysis of HEK293 lysates 15
Experimental for the syntheses 16
General 16
General synthetic procedure 17

Figure S1. GSH labeling by compound **1a**

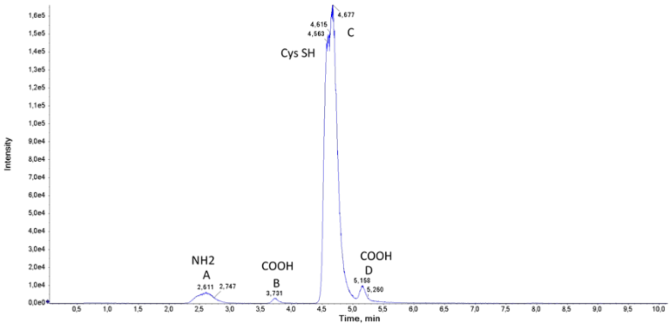

Fig 1. Total ion chromatogram of MSMS 410.1 ion (GSH + **1a** adduct) in negative ESI mode

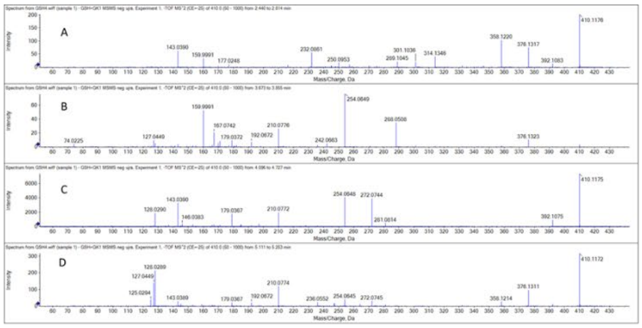

Fig 2. MSMS spectra of *m/z* 410 ion (GSH + **1a** adduct in negative ESI mode) from the Peak A-D. The binding positions were identified on the basis of differences in MSMS spectra

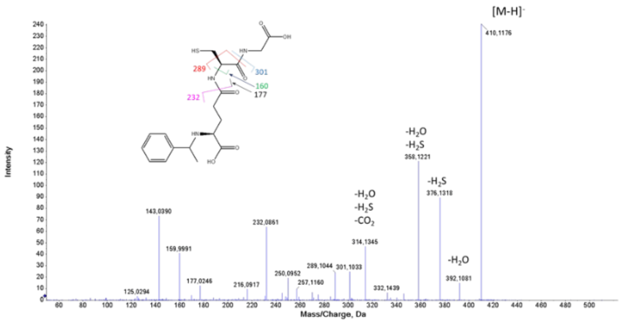

Fig 3. MSMS spectrum of 410 ion from **Peak A** : **1a** binds to GSH via amino group. *m/z* 177 and 232 ions are present only in this spectrum

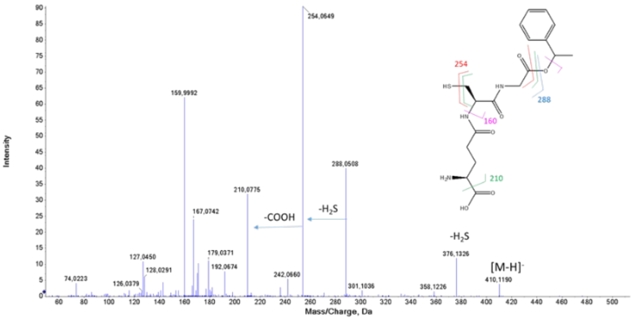

Fig 4. MSMS spectrum of 410 ion from **Peak B**. **1a** is binding to the carboxy group of Gly. Ions 288, 254 and 210 support this structure.

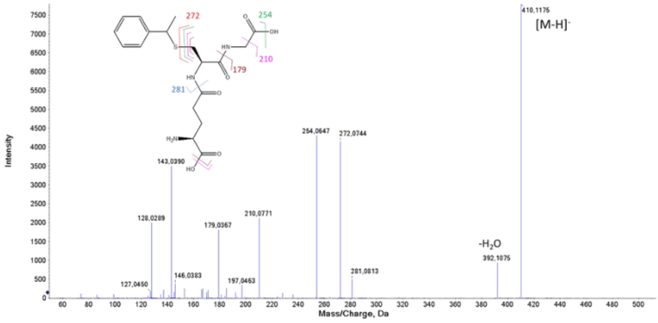

Fig 5. MSMS spectrum of 410 ion from **Peak C**. **1a** is connected via SH of cystein. This is the only MSMS spectrum where the H_2S loss from the molecular ion is not present.

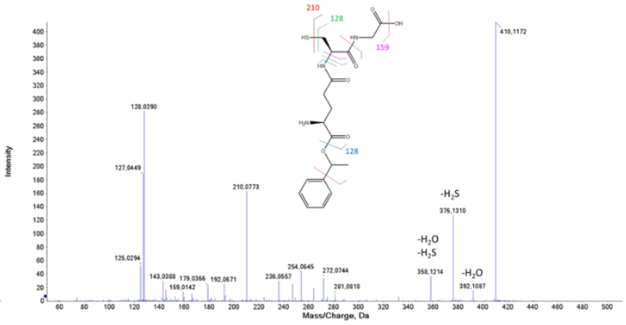

Fig 6. MSMS spectrum of 410 ion from **Peak D. 1a** is connected to the carboxyl group of Glu. Ion of 159 can only be explained from this structure.

Experimental conditions of LC-MS measurements of GSH labeling

The molecular weights of the conjugates of GSH were identified using a Triple TOF 5600+ hybrid Quadrupole-TOF LC/MS/MS system (Sciex, Singapore, Woodlands) equipped with a DuoSpray IonSource coupled with a Shimadzu Prominence LC20 UFLC (Shimadzu, Japan) system consisting of binary pump, an autosampler and a thermostated column compartment. Data acquisition and processing were performed using Analyst TF software version 1.7.1 (AB Sciex Instruments, CA, USA). Chromatographic separation was achieved on a Phenomenex Luna Omega PS C18 (50 mm × 2.1 mm, 3 µm, 100 Å) HPLC column. Sample was eluted in gradient elution mode using solvent A (0.1% formic acid in water) and solvent B (0.1% formic acid in ACN). The initial condition was 5 % B followed by a linear gradient to 55 % B by 10 min, to 95 % B by 4 min, 14 to 15.5 min 95% B was retained; and from 15.5 to 16 min back to initial condition with 10 % eluent B and retained from 16 to 18 min. Flow rate was set to 0.4 ml/min. The column temperature was 40 °C and the injection volume was 10 µL. UV- VIS

spectrometer was used in 254 nm wave length. Nitrogen was used as the nebulizer gas (GS1), heater gas (GS2), and curtain gas with the optimum values set at 40, 45 and 25 (arbitrary units), respectively. Data were acquired in negative electrospray mode in the mass range of m/z = 50 to 1000, with 0. 1s accumulation time. The source temperature was 400 °C and the spray voltage was set to -4500 V. Declustering potential value was set to -80 V. In MS2 experiment (Product Ion scan mode): the mass range was m/z=50 to 1000 with an accumulation time of 0.1 sec and with -25 V Collision Energy.

PeakView® V.2.2 software (version 2.2, Sciex) software were used to assign and evaluate the peaks in the MSMS spectra.

Figure S2: MAO-A labeling by compound **8**

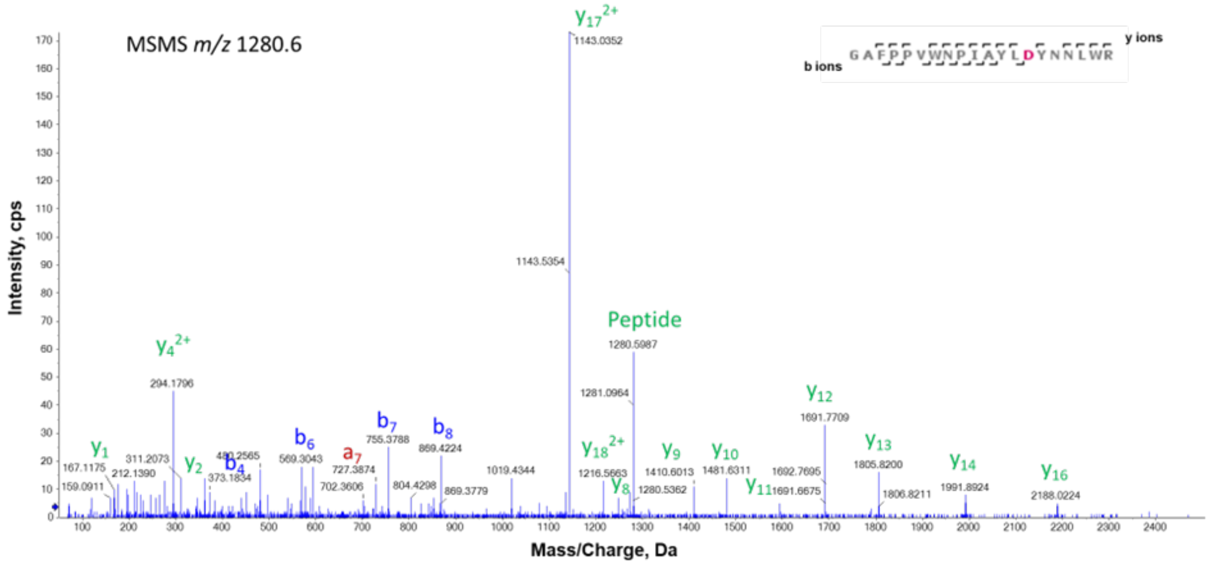

The MSMS spectrum of **8** modified MAO A peptide [110-129]. Molecule **8** binds to D123.

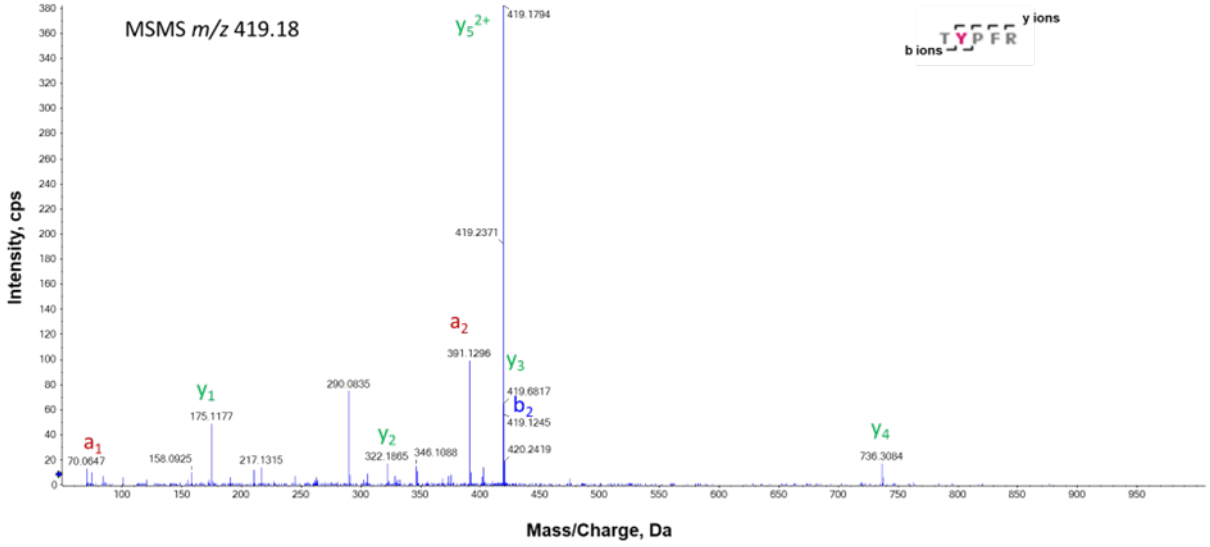

The MSMS spectrum of **8** modified MAO A peptide [105-109]. Molecule **8** binds to Y106.

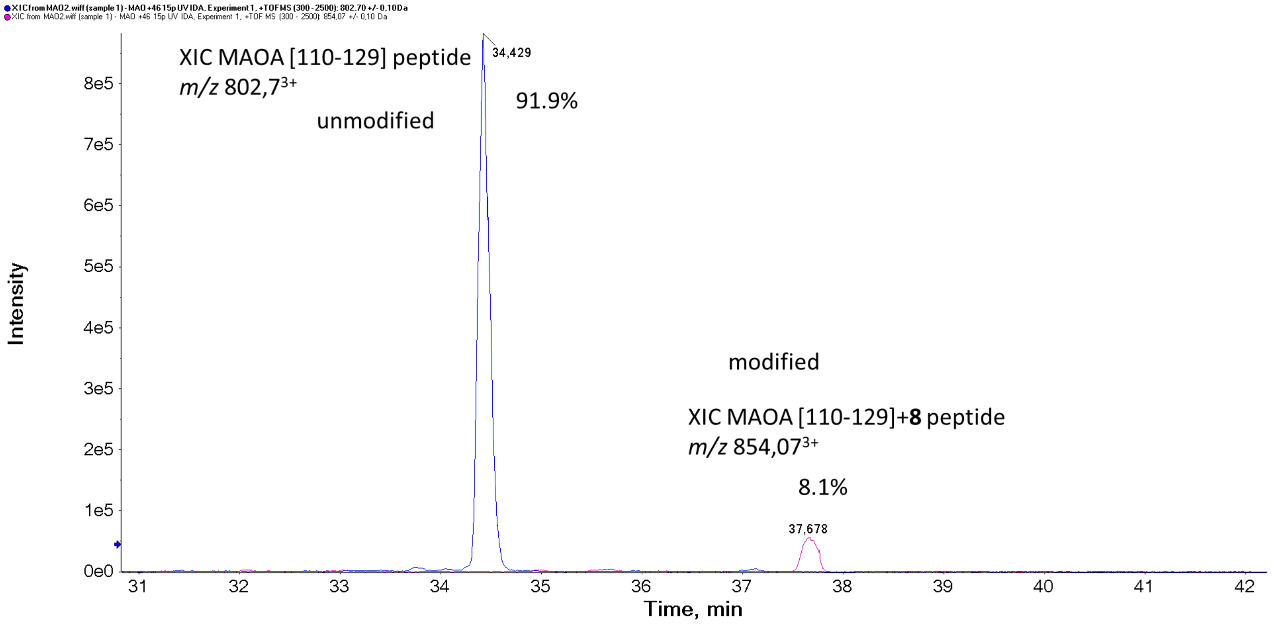

MAOA [110-129] peptide: 92/8 % unmodified/modified.

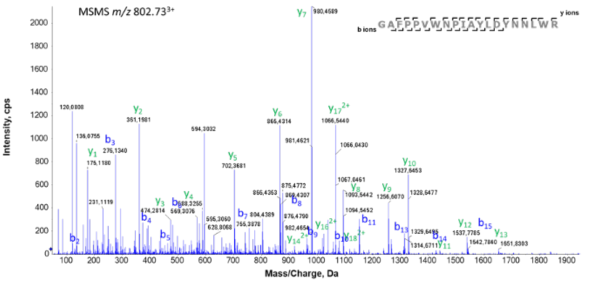

The MSMS spectrum of unmodified MAO A peptide [110-129]

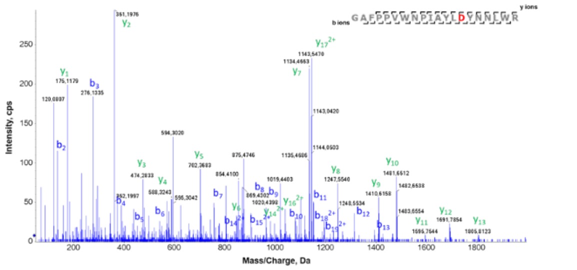

The MSMS spectrum of **8** modified MAO A peptide [110-129] The **8** fragment molecule binds to D123.

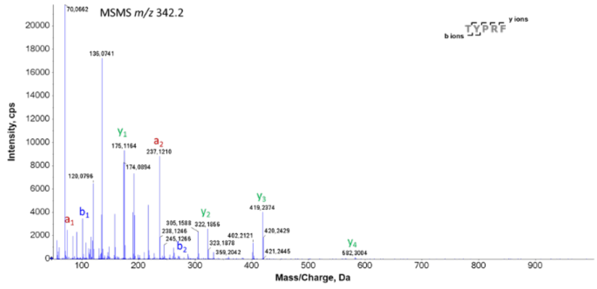

The MSMS spectrum of unmodified MAO A peptide [105-109]

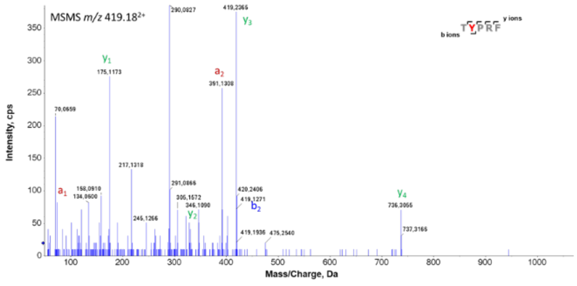

The MSMS spectrum of **8** modified MAO A peptide [105-109] The **8** fragment molecule binds to Y106.

Figure S3: STAT5B labeling by compound **10**

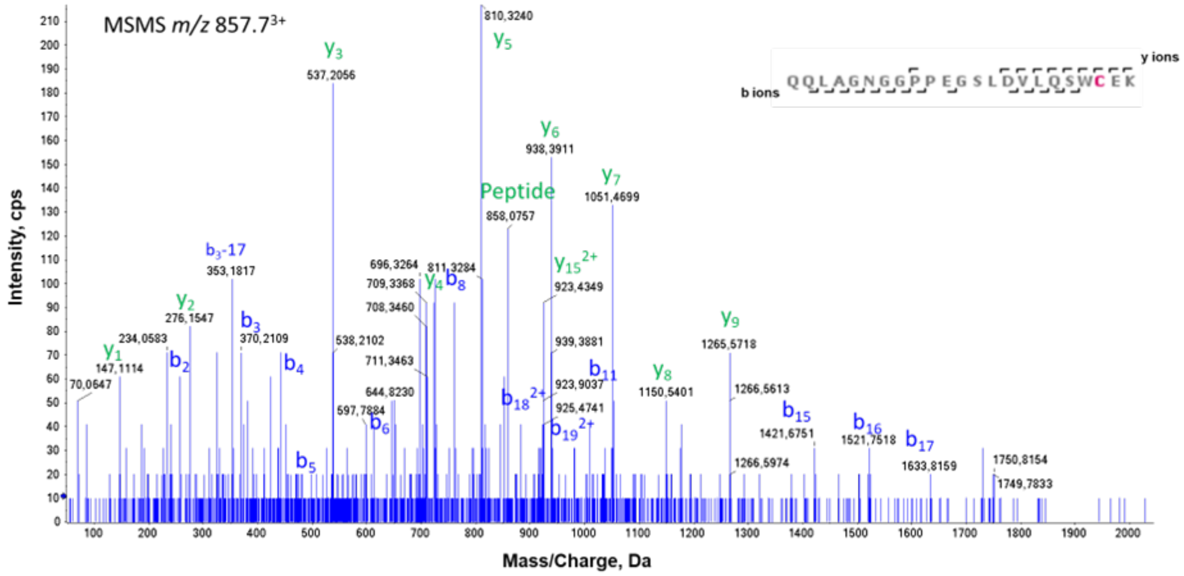

The MSMS spectrum of **10** modified STAT5b peptide [249-271]. Molecule **10** binds to C269.

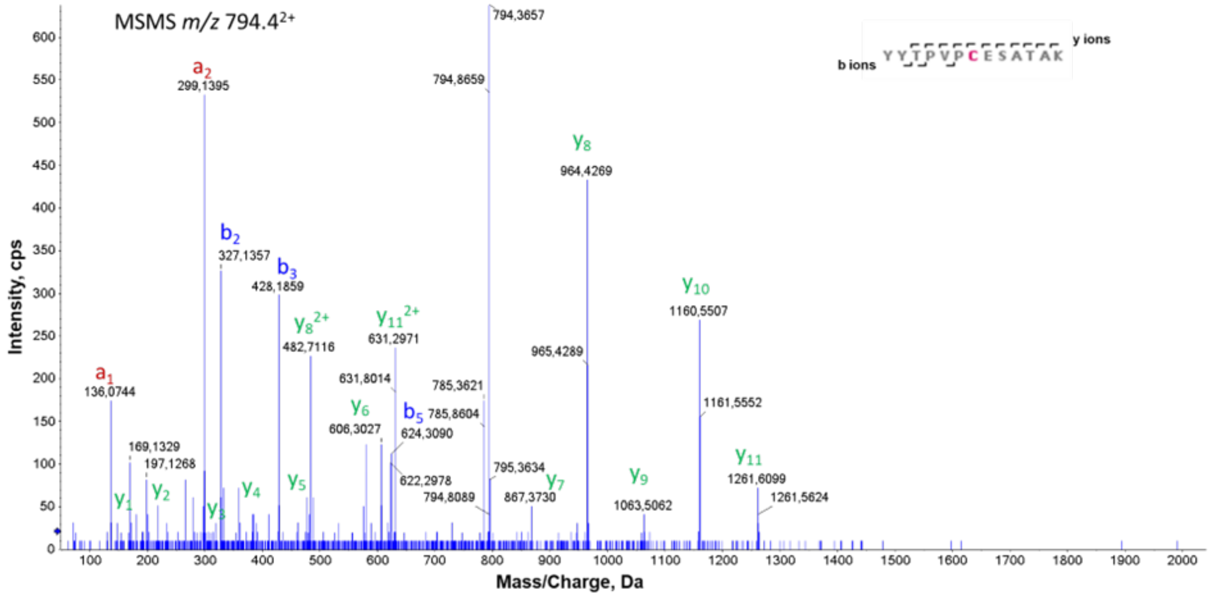

The MSMS spectrum of **10** modified STAT5b peptide [672-684]. Molecule **10** binds to C678.

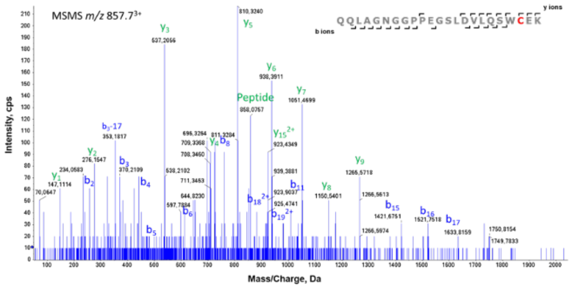

The MSMS spectrum of **10** modified STAT5b peptide [249-271] The **10** fragment molecule binds to C269.

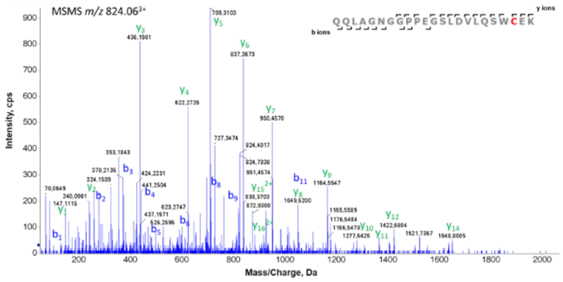

The MSMS spectrum **CAM** modified STAT5b peptide [249-271]

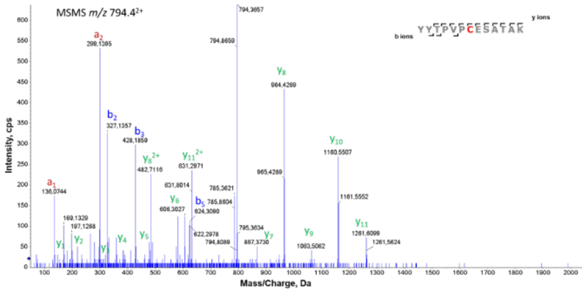

The MSMS spectrum of **10** modified STAT5 peptide [672-684] The **10** fragment molecule binds to C678.

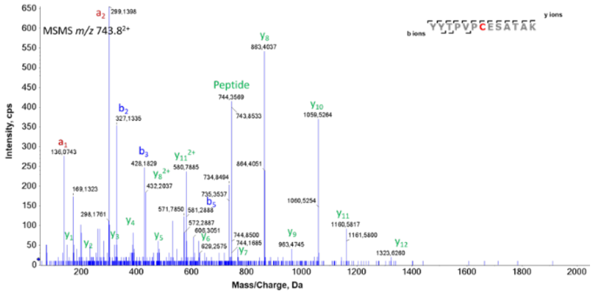

The MSMS spectrum **CAM** modified STAT5b peptide [672-684]

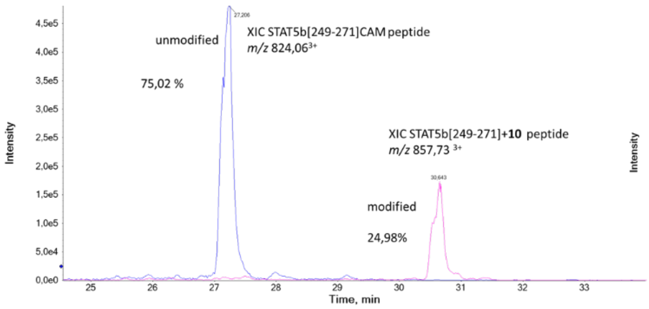

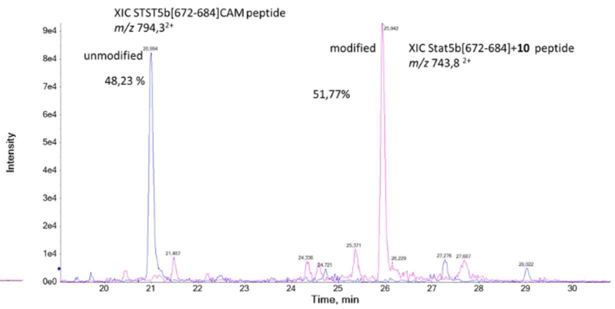

Figure S4: KRas^{G12D} labeling by compound **11**

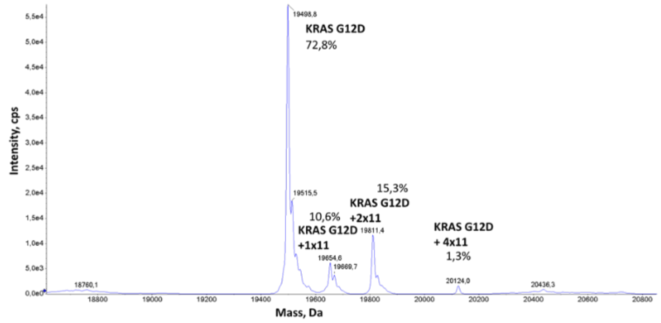

Deconvoluted mass spectrum of **11** modified KRAS G12D protein adduct.

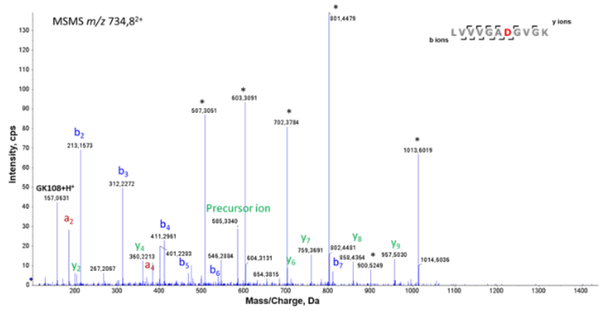

The MSMS spectrum of **11** modified KRASG12D peptide [6-16] at Rt 26.8 min. The **11** fragment molecule binds to **D12.** *:11-lost y-ion series. While the precursor mass contains the modification this loss takes places in the MS.

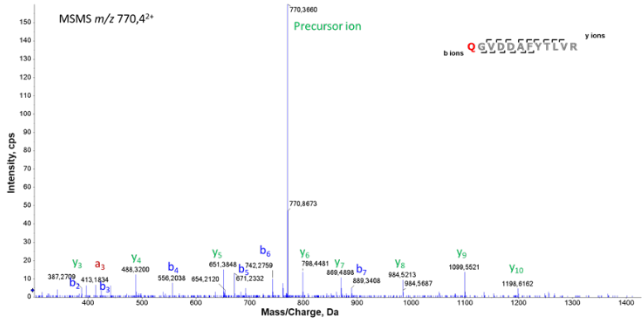

The MSMS spectrum of **11** modified KRASG12D peptide [150-161] The **11** fragment molecule binds to **Q150.**

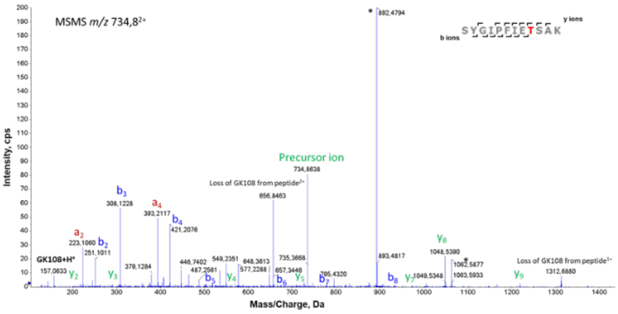

The MSMS spectrum of **11** modified KRASG12D peptide [136-147] The **11** fragment molecule binds to **T144.** *:11-lost y-ion series. While the precursor mass contains the modification this loss takes places in the MS.

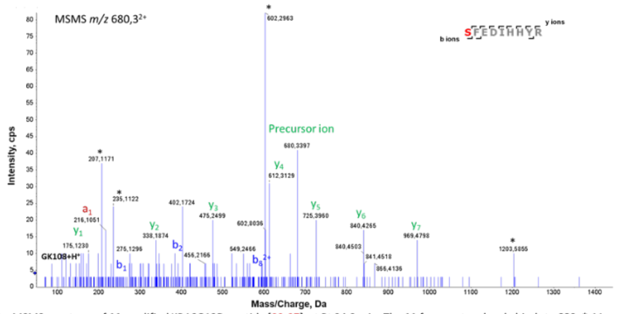

The MSMS spectrum of **11** modified KRASG12D peptide [89-97] at Rt 24,8 min. The **11** fragment molecule binds to **S89.** *:11-lost y-ion series. While the precursor mass contains the modification this loss takes places in the MS.

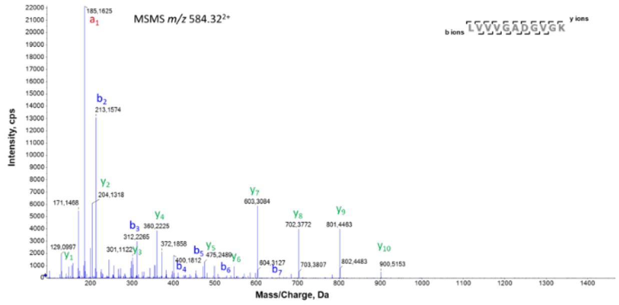

The MSMS spectrum of unmodified KRASG12D peptide [6-16].

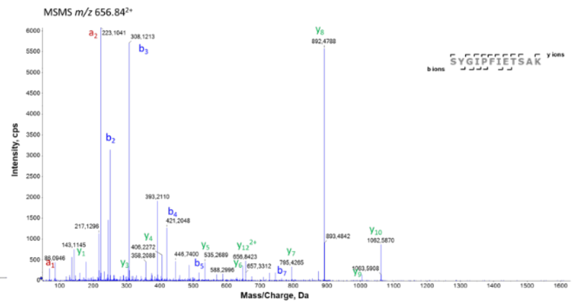

The MSMS spectrum of unmodified KRASG12D peptide [136-147]

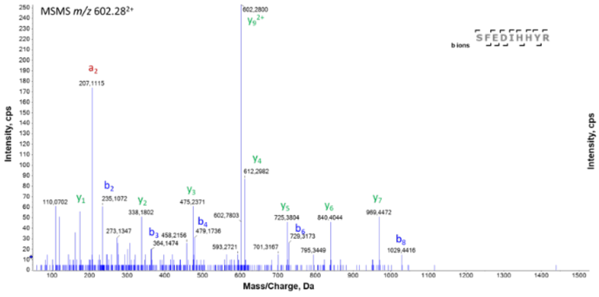

The MSMS spectrum of unmodified KRASG12D peptide [89-97]

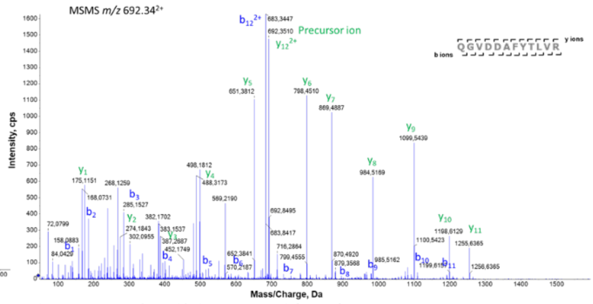

The MSMS spectrum of unmodified KRASG12D peptide [150-161]

Figure S5: AChE labeling by compound **12**

Peptide sequence	Modified?	Sequence start	Sequence end	Observed mass (Da)	Mass error (ppm)	Observed RT (min)	Intensity (cps)	Observed m/z	Charge
HRASNLVWPEWMGVIHGYE	Yes	479	497	2644.2815	-8.6	13.50	34974	661.8258	4
WMGVIHGYEIE	Yes	489	499	1696.8308	-0.6	20.37	4440201	566.2818	3
WMGVIHGYE	No	489	497	1091.5013	3.2	24.08	55291	546.2543	2
LNVWVPATPRPHNLTVMVW	Yes	121	139	2593.4145	4.6	41.93	124212	865.1430	3
VPATPRPHNLTVMVWIY	Yes	125	141	2357.2649	-4.4	22.01	29792	590.0717	4
GGGFY	Yes	142	146	863.4147	-7.6	6.98	125560	432.2110	2
GGGFYSGSSSLDVY	No	142	155	1395.6072	0.7	38.57	93612	698.3073	2

Table S1. Detailed results of chemoproteomic analysis on HEK293 lysates

Reported in a separate Excel spreadsheet.

Experimental conditions of sulfohydrazone photoactivation assay

For the photoactivation assay the hydrazone was dissolved in PBS buffer (pH 7.4) with 5% acetonitrile (in case of **8** and **10** DMSO was the co-solvent) including indoprofen as the internal standard (175 µM hydrazone, 100 µM indoprofen as final concentrations). The reaction mixture was irradiated in translucent cuvettes by using LED light sources, and analyzed by HPLC-MS. The AUC (area under the curve) values were determined via integration of HPLC or MS chromatograms and then corrected with the internal standard.

Experimental conditions of LC-MS measurements of intact protein adducts

The molecular weights of the conjugates of the protein targets were identified using a Triple TOF 5600+ hybrid Quadrupole-TOF LC/MS/MS system (Sciex, MA, USA) equipped with a DuoSpray IonSource coupled with a Shimadzu Prominence LC20 UFLC (Shimadzu, Japan) system consisting of binary pump, an autosampler and a thermostated column compartment. Data acquisition and processing were performed using Analyst TF software version 1.7.1 (Sciex Instruments, CA, USA). Chromatographic separation was achieved on a Merck BIOshell™ 400 Å Protein C18 (75 mm × 2.1 mm, 3.4 µm, 400 Å) HPLC column. Sample was eluted in gradient elution mode using solvent A (0.1% formic acid in water) and solvent B (0.1% formic acid in ACN). The initial condition was 10% B for 2 min, followed by a linear gradient to 90% B by 8 min, from 10 to 12 min 90% B was retained; and from 12 to 12.5 min back to initial condition with 10 % eluent B and retained from 12.5 to 15 min. Flow rate was set to 0.5 mL/min. The column temperature was 50 °C and the injection volume was 7 µL. Nitrogen was used as the nebulizer gas (GS1), heater gas (GS2), and curtain gas with the optimum values set at 40, 45 and 40 (arbitrary units), respectively. Data were acquired in positive electrospray mode in the mass range of m/z = 250 to 3000, with 1 s accumulation time. The source temperature was 400 °C and the spray voltage was set to 5000 V. Declustering potential value was set to 80 V. Peak View Software® V.2.2 (Sciex, Redwood City, CA, USA) was used for deconvoluting the raw electrospray data to obtain the neutral molecular masses.

MAO-A labeling protocol

Human recombinant MAO-A (50 µM nominal concentration) in 50 mM sodium phosphate buffer at pH 7.8 together with 300 mM NaCl, 20 mM imidazole, 0.05 % FOS-choline-12 and 40 % glycerol was used for the labelling. The enzyme was expressed in *Pichia pastoris* and purified following published protocols (M. Li, F. Hubálek, P. Newton-Vinson, D. E. Edmondson, *Protein Expression Purif.* 2002, **24**, 152–162.). The MAO-A sample was quickly thawed from −78 °C to 37 °C, and centrifuged (5 min at 7 500 g) in order to remove the aggregated protein. MAO-A was buffer exchanged to 50 mM K_3PO_4 at pH 7.5 together with 0.25 % Triton X-100 and stored on ice. The inhibitor was added in 0.5 µL DMSO (100 mM) to 50 µL of the enzyme solution to reach 50-fold excess of the small molecule. The samples were incubated at 4 °C for 24 h and illuminated with a 380 nm wavelength LED light source (8 W) at 4 °C for 10 minutes.

STAT5b labeling protocol

The STAT5b protein and the inhibitor were incubated at 37 °C for 1 hour. The final concentration of the protein was 15.3 µM and that of the inhibitor was 1.5 mM, the final DMSO content was 5%. After the incubation the mixture was illuminated with a 380 nm wavelength LED light source (8 W) at 4 °C for 10 minutes.

STAT5b biochemical assay

Fluorescence polarization assays were performed on a Molecular Devices SpectraMax iD5 Multimode Microplate Reader (San Jose, CA, USA) using Greiner black 384-well flat-bottom non-binding microplates with 40 µL final well volumes. The fluorescent peptide (5-FAM-G(pTyr)LVLDKW-NH_2, purchased from GenScript Biotech Ltd., Piscataway, NJ, USA), as well as the protein were diluted with a buffer containing 50 mM NaCl, 10 mM HEPES (4-(2-hydroxyethyl)-1-piperazineethanesulfonic acid), 1mM EDTA (ethylenediaminetetraacetic acid), 2 mM DTT (dithiothreitol) and 0.1% Triton® X-100, pH 7.5. The final concentration of the STAT5b protein was 125 nM, and the fluorescent peptide was added at a final concentration of 10 nM. The wells were treated with varying concentrations of inhibitor compounds with a 5% final DMSO content. Protein and inhibitors were incubated at 37 °C for 1 hour, then illuminated with a 380 nm wavelength LED lamp (8 W) at 5 °C for 10 minutes. After the illumination the fluorescence peptide was added, then the plate was incubated for another 20 minutes prior to the fluorescence readout (extinction wavelength: 475 nm, emission wavelength: 520 nm). The measurements were carried out using 3 parallel biological replicas. Fluorescence polarization was calculated from the perpendicular and parallel fluorescence intensities, then

plotted against concentration and the IC_{50}s were determined after fitting quadratic dose-response curves on data points.

$KRas^{G12D}$ labeling protocol

The $KRas^{G12D}$ protein and the inhibitor were incubated at room temperature for 1 hour. The final concentration of the protein was 7 μM and that for the inhibitor was 700 μM, the final DMSO content was 5%. After the incubation the mixture was illuminated with a 380 nm wavelength LED light source (8 W) at 4 °C for 10 minutes.

Induced fit docking

X-ray structure of $KRas^{G12D}$ in complex with MRTX-1133 (PDBID: 7RPZ) was used to perform the docking simulations. Protein was prepared with Protein Preparation Wizard (Schrödinger Release 2021-3) using default methods. Ligands were prepared with Schrödinger Ligprep (Schrödinger Suite 2021-3: LigPrep, Schrödinger, LLC, New York, NY,. 2021.) using default methods. Docking was performed with Schrödinger Induced Fit Docking (Schrödinger Release 2021-3, Farid, R.; Day, T.; Friesner, R. A.; Pearlstein, R. A. New Insights about HERG Blockade Obtained from Protein Modeling, Potential Energy Mapping, and Docking Studies. *Bioorganic and Medicinal Chemistry* **2006**, *14* (9), 3160–3173. https://doi.org/10.1016/j.bmc.2005.12.032.; Sherman, W.; Day, T.; Jacobson .P, M.; Friesner A., R.; Farid, R. Novel Procedure for Modeling Ligand/Receptor Induced Fit Effects. **2005**. https://doi.org/10.1021/JM050540C.; Sherman, W.; Beard, H. S.; Farid, R. Use of an Induced Fit Receptor Structure in Virtual Screening. *Chemical Biology and Drug Design* **2006**, *67* (1), 83–84. https://doi.org/10.1111/j.1747-0285.2005.00327.x.; Schrödinger Release 2021-3: Induced Fit Docking Protocol. Schrödinger, LLC, New York, NY 2021.). The receptor box was defined as centroid of selected amino acids using the amino acids detected by LCMS/MS. 20 possible binding conformations were generated. Redocking was done into structures within 30 kcal/mol of the best structure, and within the top 20 structures overall using single precision method.

AChE biochemical assay

To assess the inhibition activity of the test compounds we utilized Sigma-Aldrich Acetylcholinesterase Inhibitor Screening Kit (Cat#MAK324, St. Louis, MO, USA) and we purchased Electrophorus electricus Acetylcholinesterase (*ee*AChE) from Sigma-Aldrich (Cat#C3389-500UN, St. Louis, MO, USA). Briefly acetyl-thiocholine was applied as substrate, and while the *ee*AChE catalyzed its hydrolysis, dithiobis(2-nitrobenzoic acid) (DTNB or Ellmann's reagent) was applied to monitor the free thiol of the thiocholine generated in-situ by

the enzyme reaction The assay was performed as follows. In 96-well microtiter plates (transparent, F-bottom, Greiner #655101) 5 µL of test compounds were added from stock solutions in DMSO, next 45 µL of 400 U/L *ee*AChE (diluted in assay buffer) was added and incubated for 30 minutes, then photoactivation of the samples were performed *via* illumination with a 380nm wavelength LED lamp (8W) at 4 °C for 10 minutes. Next 150 µL of the predissolved Reaction Mixture was added containing of the enyme substrate and DTNB, both, and the absorbance at 412 nm was measured immediately and after 10 min. The measurements were carried 2 parallels and the IC_{50}s were determined after fitting quadratic dose-response curves on data points.

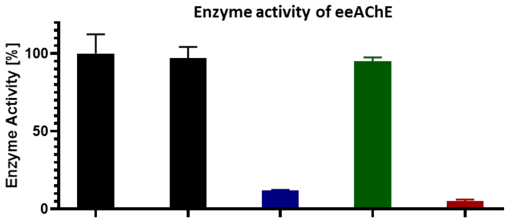

AChE	+	+	+	+	+
Donepezil	-	-	+	-	-
12	-	-	-	+	+
hv	-	+	-	-	+
eeAChE IC50 (nM) or Enzyme Activity % at 10 µM			152±49 nM	95±3%	379±119 nM

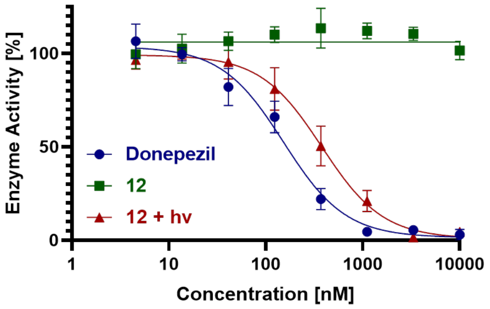

AChE labeling protocol

For the MS experiments, isolated *Electrophorus electricus* Acetylcholinesterase (eeAChE) was purchased from Sigma-Aldrich (Cat#C3389-500UN, St. Louis, MO, USA). and dissolved 1 mg/mL in 100 mM TRIS buffer (pH 7.5) and treated with 50 eq. of the probe (added from 10 mM stock solutions in DMSO) and incubated for 30minutes, then photoactivation of the samples were performed via illumination with a 380 nm wavelength LED lamp (8 W) at 4 °C for 10 minutes. Finally, the samples were frozen immediately after labelling experiments and stored at -80 °C until MS experiments.

Peptide mapping: Modification sites were determined by proteolysis and RPLC-MS peptide mapping. The protein was enzymatically digested after buffer exchange using Amicon Ultra-0.5 mL Centrifugal Filter units (10 KDa, Merck Millipore). GluC and chymotrypsin (Promega Corporation, Madison, USA) enzymes were used for the digestions. Briefly, protein samples were reduced by dithiothreitol at 37 °C for 30 min. After reduction, the protein were digested using 1:25 enzyme:protein ratio. Overnight digestion was performed in 50 mM NH_4HCO_3 solution at 37 °C. Digestion was stopped by adding formic acid in a final concentration of 0.2%

(V/V). Gradient elution was performed on a Waters Acquity CSH Peptide C18 UPLC column (2.1x150 mm, 1.7 µm) under the following parameters: mobile phase “A”: 0.1% formic acid in water, mobile phase “B”: 0.1% formic acid in acetonitrile; flow rate: 300 µL/min; column temperature: 60 °C; gradient: 2 min: 2%B, 80 min: 45%B, 81 min: 85%B. MS^E experiments were preformed using collision voltage ramping. MS data acquisition was performed under the following parameters: *m/z* 50-2000, scan time: 0.3 sec, single Lock Mass: leucine enkephalin; low energy: 6V, high energy: ramping 19-45V. BiopharmaLynx 1.3.5 software (Waters Corp., Wilmslow, U.K.) was used to for data analysis.

The labeling levels at specific sites of the protein were determined by comparing the total intensity of all modified peptides to the intensity of all unmodified peptides identified during the LC-MS peptide mapping.

Cell culture and lysate production for chemoproteomics

Lysate samples for proteomics analysis were prepared according to literature protocol (DOI: 10.3390/molecules28073042) with slight modifications. In details cells were grown in RPMI 1640 medium at 37 °C in a humidified atmosphere with 5% CO_2, as described. When the confluency of HEK293 cells reached 70%, the growth medium was discarded and cells were washed twice with phosphate-buffered saline (PBS; Biosera), harvested in 5 mL PBS (Biosera) using a cell scraper (Sarstedt) and collected in a 15 mL conical tube (Sarstedt). Then, cells were centrifuged for 5 min at 1000× g at 4 °C (Eppendorf 5804R centrifuge, Eppendorf AG, Hamburg, Germany), the supernatant was removed, and to the pellet was added a protease and phosphatase inhibitor cocktail mix (Thermo Scientific, Rockford, IL, USA). The cell pellet was resuspended in a 4× volume of PBS (Biosera) on ice, transferred into a 1.5ml tube (Corning, Reynosa, Mexico) and homogenized using a probe sonicator (model Q55, QSonica, Newtown, CT, USA), 10× for 1 sec at 20% power, on ice. After this, centrifugation was applied for 5 min at 12,000× g at 4 °C (Heraeus Biofuge Fresca centrifuge, Kendro Laboratory Products, Osterode, Germany) to pellet nuclei and unbroken cells. The supernatant was transferred to an ultracentrifuge sealing tube (OptiSeal, Beckman Coulter, Brea, CA, United States) and centrifuged for 1 h at 100,000× g at 4 °C (Optima MAX-XP Ultracentrifuge, Beckman Coulter) to separate the membrane (pellet) and soluble (supernatant) protein fractions. The supernatant fraction was transferred to a clean 1.5 mL tube (Corning), while the pellet was washed with cold PBS and resuspend in cold PBS (Biosera). The protein concentrations in the membrane and the soluble fractions were quantified by a Pierce BCA protein assay kit (Thermo Scientific) according to the manufacturer’s protocol, and the protein concentration was adjusted to the

desired concentration (4 mg/mL) with cold PBS (Biosera). Samples were stored at −80 °C. Prior to ABPP probe labelling, samples were thawed on ice and briefly allowed to warm up to room temperature.

HEK293 lysate labelling for chemoproteomics

500-500 µg lysates were diluted with PBS until 4 mg/mL and **4b** probe was added at 500 µM for labelling characterization of HEK293 lysates. DMSO-treated controls and labelled samples were prepared in 3-3 parallel. After 60 min incubation in dark at room temperature the mixtures were illuminated with 380 nm LED for 10 min at room temperature. Next, the samples were treated with 1.8 µL of 50 mM azido-PEG3-SS-biotin (500 µM final concentration), 2.4 µL of 75 mM Cu(II)SO_4 aqueous solution (1 mM final concentration), 9 µL of 100 mM THPTA solution (5 mM final concentration), 9 µL of 100 mM aminoguandine hydrochloride (5 mM final concentration), 9 µL of freshly dissolved 100 mM sodium-ascorbate solution (5 mM final concentration) and 28.8 µL PBS (pH=7.4) and the reaction samples were incubated further for 90 min at room temperature. Next 7x volume of ice-cold acetone was added to the samples and left the protein pellets precipitate for overnight storage at -20 °C. Next morning, the supernatant was discarded and the pellets were washed with ice-cold acetone:water 7:1 multiple times. Finally, the pellets were resuspended with 50 mM AMBIC supplemented with 1% SDS, incubated 10 min at 50 °C then sonicated for 3×1 min in ultrasound bath, repeated 3-times the latter steps. Samples were saved for dot blot analysis and the rest were then enriched on High-Capacity Neutravidin Agarose beads [DOI: 10.3390/ijms24010273], with addition of 70 µL slurry (transferred into Pierce Spin-Column, Thermo Fisher Scientific) and incubated for 60 min at room temperature with gently shaking. Affinity columns were then washed extensively as follows: 3×300 µL with 50 mM AMBIC buffer, next 3×300 µL with 1M NaCl aqueous solution and finally again 3×300 µL with 50 mM AMBIC buffer. The enriched peptides were eluted by a reducing agent by 10 mM Dithiothreitol (DTT, Thermo Fisher Scientific) in 50 mM AMBIC buffer in two consecutive incubations of 30 min, each at 37 °C, which were followed by an alkylation step with 25 mM iodoacetamide in dark at 37 °C for additional 30 min. The samples were then supplemented with 7x volume of ice-cold acetone and left the enriched protein pellets precipitate for overnight storage at -20 °C. Next morning, the supernatant was discarded and the pellets were washed with ice-cold acetone:water 7:1 multiple times. Finally, the pellets were resuspended with 50 mM AMBIC supplemented with 0.1% RapiGest-SF surfactant, sonicated for 3×1min in ultrasound bath. The proteins were then treated with trypsin (each sample set to reach 1:25 ratio) and incubated overnight (14 h) at 37 °C. Next, the samples

were dried using a SpeedVac. The residues were dissolved in LC-MS grade water containing 0.1% TFA and desalted with Pierce C-18 agarose packed column, which was activated with LC-MS grade methanol, then pre-equilibrated with LC-MS-grade water containing 0.1% TFA. C18 spin columns were loaded with the samples and washed 3 times with LC-MS grade water containing 0.1 % TFA and subsequently eluted with 2×50 µL 70% acetonitrile, 0.1% TFA and dried using a Speedvac.

Dot blot analysis of HEK293 lysates

We have developed a Dot blot analysis to compare the biotinylation levels of the isolated protein mixtures in the DMSO (control) or **4b** probe pre-treated HEK293 lysates (biotinylation process is detailed under the "Sulfohydrazones HEK293" subheading). Based on protein measurement (literature method [PMID: 14907713] was applied, using bovine serum albumin as a standard) serial dilutions were prepared (at least three dilutions per sample) between 0.8 µg to 0.2 µg protein content and stored them on ice for later use. Thereafter the PVDF membrane (0.22 µm pore size) was activated with methanol for 5 min in plastic container under constant shaking. Then the methanol was discarded and the membrane was equilibrated by TBS-T buffer (25 mM Tris-HCl, pH=7.4, 2.7 mM KCl, 137 mM NaCl, 0.05% (V/V) Tween-20), incubate for 5 min again. The PVDF membrane was inserted into the Bio-rad 96-well Bio-Dot microfiltration device (we used a TBS-T wetted filter paper under the PVDF membrane to avoid its drying) and the diluted samples were loaded into the wells. The biotinylated protein samples were directly immobilized onto the PVDF membrane using a vacuum pump conducted Bio-Dot microfiltration apparatus. After two minutes the vacuum pump was turned off and the PVDF membrane was put back into the plastic container under TBS-T solution (it was rinsed three times consecutively). Finally, the membrane was blocked with 2% (m/V) BSA-TBS-T buffer for 45 min at room temperature, then blocking buffer was discarded and treated by HRP conjugated streptavidin (Thermo Fisher Scientific, diluted 50000-fold in the blocking buffer) for 45 min in dark. Then the PVDF was washed three times with TBS-T for 10 minutes. The biotinylation efficiency was visualized by the enhanced chemiluminescence reagent (Merck Millipore Ltd, luminol and peroxide solutions were mixed in equal volumes immediately before use), and the images were captured by a ChemiDoc XRS+ Imaging system (Bio-Rad). The intensity of the spots were analysed by Image Lab 6.0 software.

LC-MS/MS analysis of HEK293 lysates

All measurements were carried out on a Waters ACQUITY UPLC M-Class LC system (Waters, Milford, MA, United States) coupled with an Orbitrap Exploris 240 mass spectrometer (Thermo

Fisher Scientific, Waltham, MA, United States). Symmetry C18 (100 Å, 5 µm, 180 µm × 20 mm) trap column was used for trapping and desalting the samples. Chromatographic separation of peptides was accomplished on an ACQUITY UPLC M-Class Peptide BEH C18 analytical column (130 Å, 1.7 µm, 75 µm × 250 mm) at 45 °C by gradient elution. Water (solvent A) and acetonitrile (solvent B), both containing 0.1% formic acid were used as mobile phases at a flow rate of 200 nL/min. The sample temperature was maintained at 5 °C. The mass spectrometer was operated using the equipped Nanospray Flex Ion Source. Data were collected using the data-dependent acquisition (DDA) method with MS1 scan between 360 and 2200 Th using 60,000 resolution, while ddMS2 scans with isolation windows of 2 Th were collected at 30,000 resolution keeping a 3 s cyícle time. Data acquisition was performed using Xcalibur™ 4.6 (Thermo Fisher Scientific, Waltham, MA, United States). Raw LC-MS data files were processed using Fragpipe v22.0 [DOI: 10.1038/nmeth.4256]. Uniprot Human reference proteome assuming 2 missed cleavage sites was used (20,575 proteins) for protein identification. A mass offset analysis was performed assuming Met oxidation, pyro Glu as variable and carbamidomethyl Cys as fixed modification and a mass offset of 520.24677 caused by **4b** was monitored. Protein quantification was performed within Fragpipe using IonQuant with default settings [DOI: 10.1038/s41598-018-31154-6; 10.1038/nmeth.3901] and enabling match between runs. Protein hits identified with at least unique peptides and quantified in all replicates of any group were involved in further analysis.

Experimental for the syntheses

General

All chemicals and solvents with >95% purity were purchased from commercial vendors (Sigma-Aldrich (Budapest, Hungary), Fluorochem (Hadfield, UK), Combi-Blocks (San Diego, CA, USA)) and used without further purification. ^{1}H NMR and ^{13}C NMR spectra were recorded in DMSO-d6, CD3CN, or D2O solution at room temperature on a Varian Unity Inova 500 spectrometer (500 and 125 MHz for ^{1}H NMR ^{13}C NMR spectra, respectively), with the deuterium signal of the solvent as the lock. Chemical shifts (δ) and coupling constants (*J*) are given in ppm and Hz, respectively. HPLC-MS measurements were performed using a Shimadzu LC-MS-2020 device equipped with a Reprospher-100 C18 (5 µm; 100 x 3 mm) column and a positive–negative double ion source (DUIS±) with a quadrupole MS analyzer in a range of m/z 50–1000. The sample was eluted with gradient elution using eluent A (0.1% formic acid in water) and eluent B (0.1% formic acid in acetonitrile). Flow rate was set to 1 mL/min. The initial condition was 0% B eluent, followed by a linear gradient to 100% B eluent by 1 min;

from 1 to 3.5 min, 100% B eluent was retained, and from 3.5 to 4.5 min, we went back to 5% B eluent, and this was retained from 4.5 to 5 min. The column temperature was kept at room temperature, and the injection volume was 1–10 μL. Purity of compounds was assessed by HPLC with UV detection at 254 nm; all tested compounds were >95% pure. High-resolution mass spectrometric measurements were performed using a Q-TOF Premier mass spectrometer (Milford, MA, USA) in positive or negative electrospray ionization mode. Reactions were monitored with Merck silica gel 60 F254 TLC plates (Darmstadt, Germany). The column chromatography purifications were performed using Teledyne ISCO CombiFlash Lumen+ Rf.

General synthetic procedure

In general, the sulfonylhydrazide and the ketone/aldehyde were dissolved in ethanol or methanol and stirred at room temperature, or reflux. In case of heating, catalytic amount of 1M HCl or p-toluene sulfonic acid was added to the mixture. The reactions were followed with HPLC-MS and TLC. After completion of the reaction the product was collected through filtration or evaporating the solvent. In some cases, the crude product was then purified by normal-phase flash chromatography or preparative HPLC. For specific syntheses see detailed reactions below.

4-Methyl-*N′*-(propan-2-ylidene)benzenesulfonohydrazide (**2a**)

In a round bottom flask 4-methylbenzenesulfonohydrazide (3.21 g; 17.24 mmol) and acetone (1.00 g; 17.24 mmol; 1.28 mL) were dissolved in 35 mL methanol. The resulting mixture was stirred at room temperature for 4 hours. After completion of the reaction the product was collected by filtration.

Yield: 3.60 g (92%); pale yellow solid; ^{1}H NMR (300 MHz, DMSO-d_6) δ 9.96 (s; 1H); 7.73 (d; J = 8.3 Hz; 2H), 7.39 (d; J = 8.0 Hz; 2H), 2.39 (s; 3H), 1.80 (s; 3H), 1.79 (s; 3H) ppm.

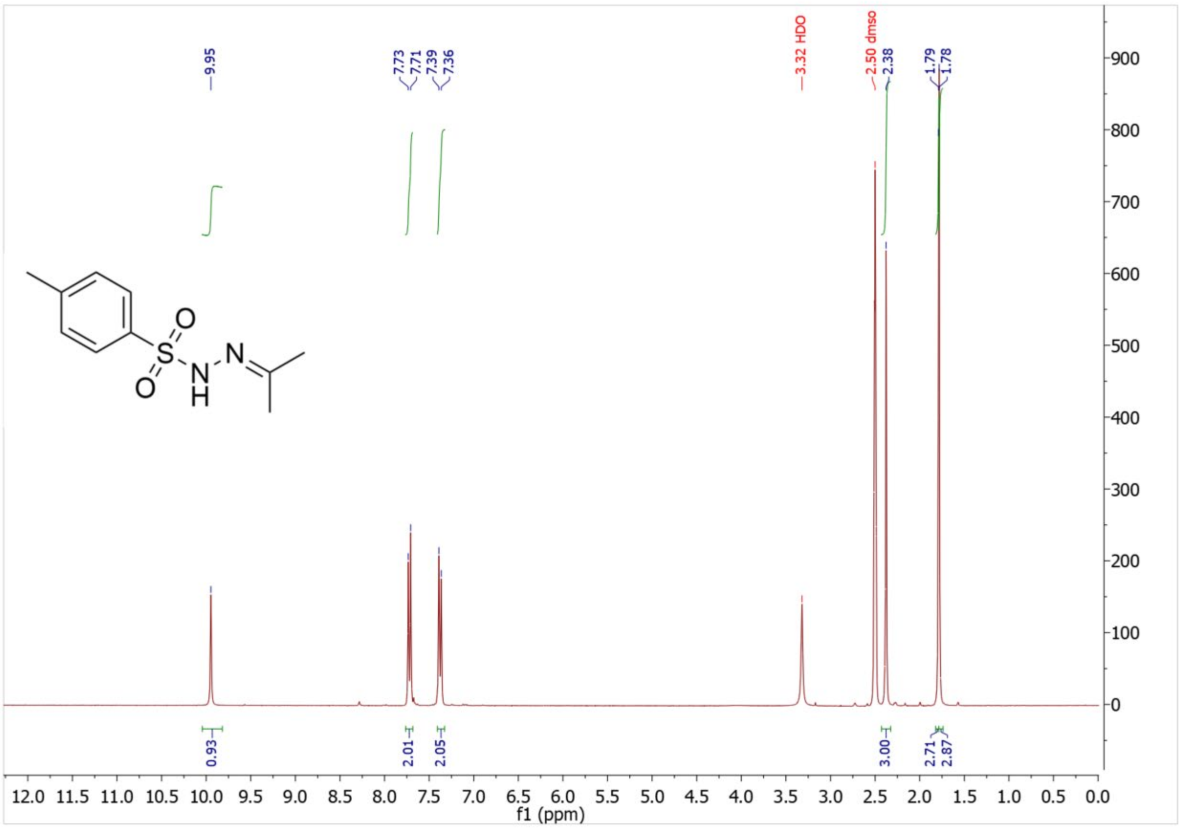

4-Methyl-*N'*-(1,1,1-trifluoropropan-2-ylidene)benzenesulfonohydrazide (**2b**)

In a round bottom flask 4-methylbenzenesulfonohydrazide (0.37 g; 2.00 mmol) and 1,1,1-trifluoropropan-2-one (0.22 g; 2.00 mmol) were dissolved in 2 mL ethanol. The resulting mixture was stirred at 70 °C for 5 hours. After completion of the reaction the mixture was cooled down, and the product was collected by filtration. The filtrate was purified by normal-phase flash chromatography.

Yield: 0.25 g (44%); white solid; ^{1}H NMR (500 MHz, DMSO-d_6) δ 11.41 (s, 1H), 7.72 (d, *J* = 8.0 Hz, 2H), 7.43 (d, *J* = 8.0 Hz, 2H), 2.39 (s, 3H), 1.99 (s, 3H) ppm.

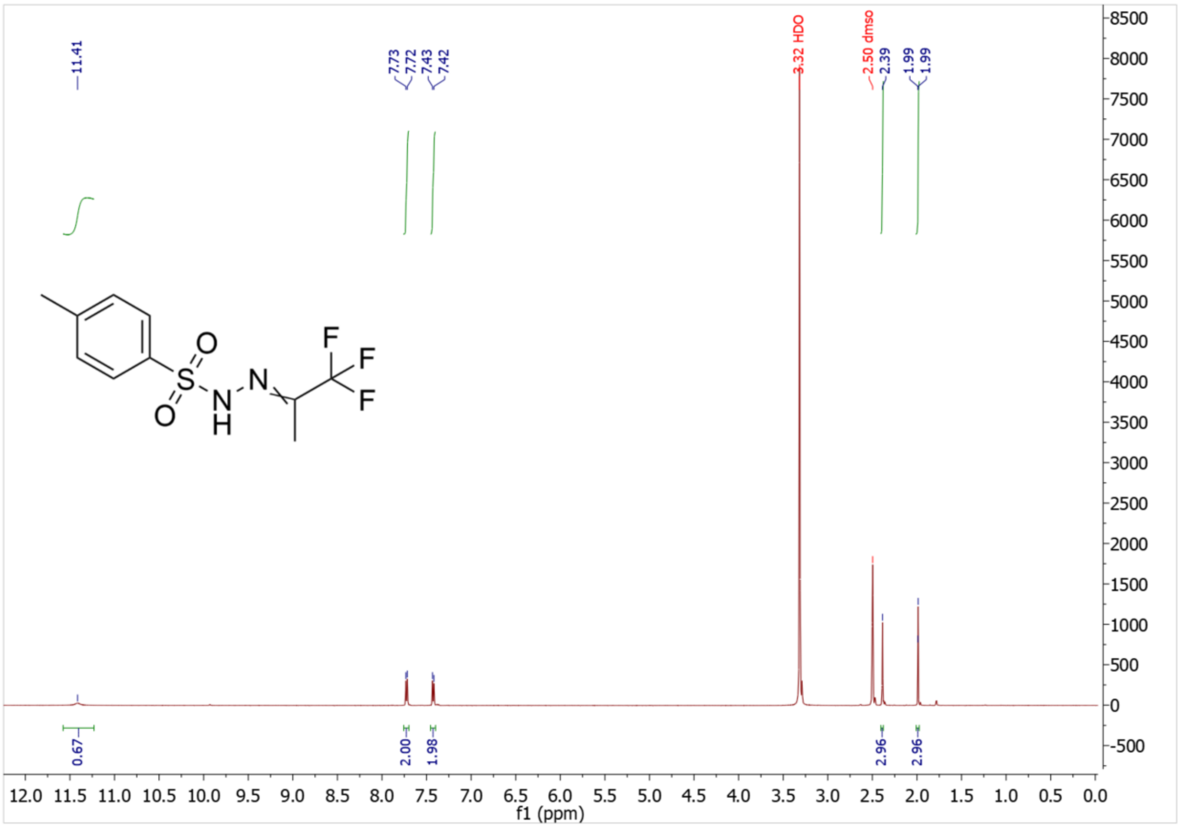

N'-(3,3-dimethylbutan-2-ylidene)-4-methylbenzenesulfonohydrazide (**2c**)

In a round bottom flask 4-methylbenzenesulfonohydrazide (0.37 g; 2.00 mmol) and 3,3-dymethylbutan-2-one (0.20 g; 2.00 mmol) were dissolved in 2 mL methanol. The resulting mixture was stirred at 60 °C for 2 hours. After completion of the reaction the mixture was cooled down, and the product was collected by filtration. The filtrate was purified by normal phase flash chromatography.

Yield: 0.30 g (55%); white solid; ^{1}H NMR (300 MHz, DMSO-d_6) δ 9.76 (s; 1H), 7.72 (d; J = 8.3 Hz; 2H), 7.38 (d; J = 8.0 Hz; 2H), 2.38 (s; 3H), 1.73 (s; 3H), 0.95 (s; 9H) ppm.

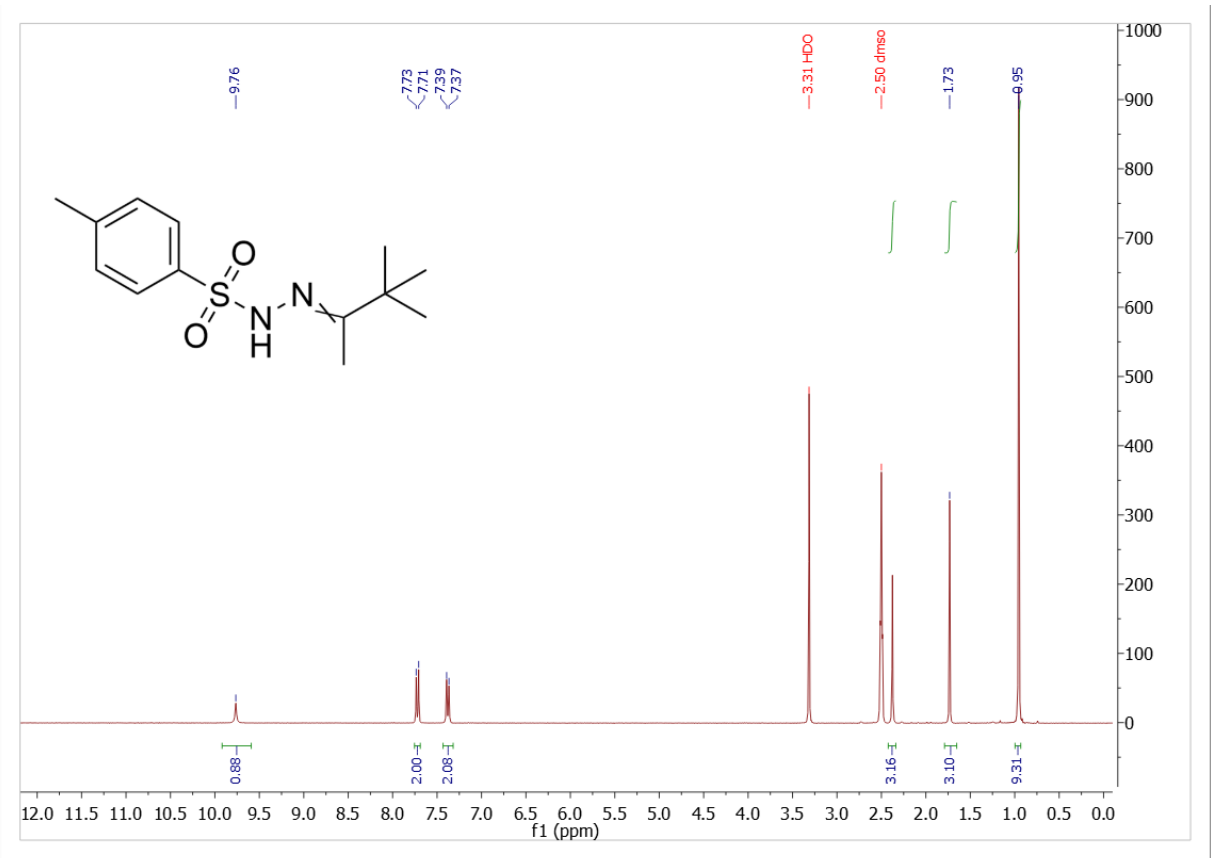

4-Methyl-*N′*-(1-phenylpropan-2-ylidene)benzenesulfonohydrazide (**2d**)

In a round bottom flask 4-methylbenzenesulfonohydrazide (1.42 g; 7.62 mmol) and 4-methyl-*N′*-(1-phenylpropan-2-ylidene)benzenesulfonohydrazide (1.00 g; 7.62 mmol; 0.12 mL) were dissolved in 15 mL ethanol. The resulting mixture was stirred at room temperature for 1 days. After completion of the reaction the product was collected by filtration.

Yield: 0.11 g (55%); white solid; ^{1}H NMR (500 MHz, DMSO-d_6) δ 10,00 (s; 1H), 7.72 (d; J = 7.9 Hz; 2H), 7.39 (d; J = 8.0 Hz; 2H), 7.25 – 7.14 (m; 3H), 7.01 – 6.89 (m; 2H), 3.38 (s; 2H), 2.41 (s; 3H), 1.69 (s; 3H) ppm.

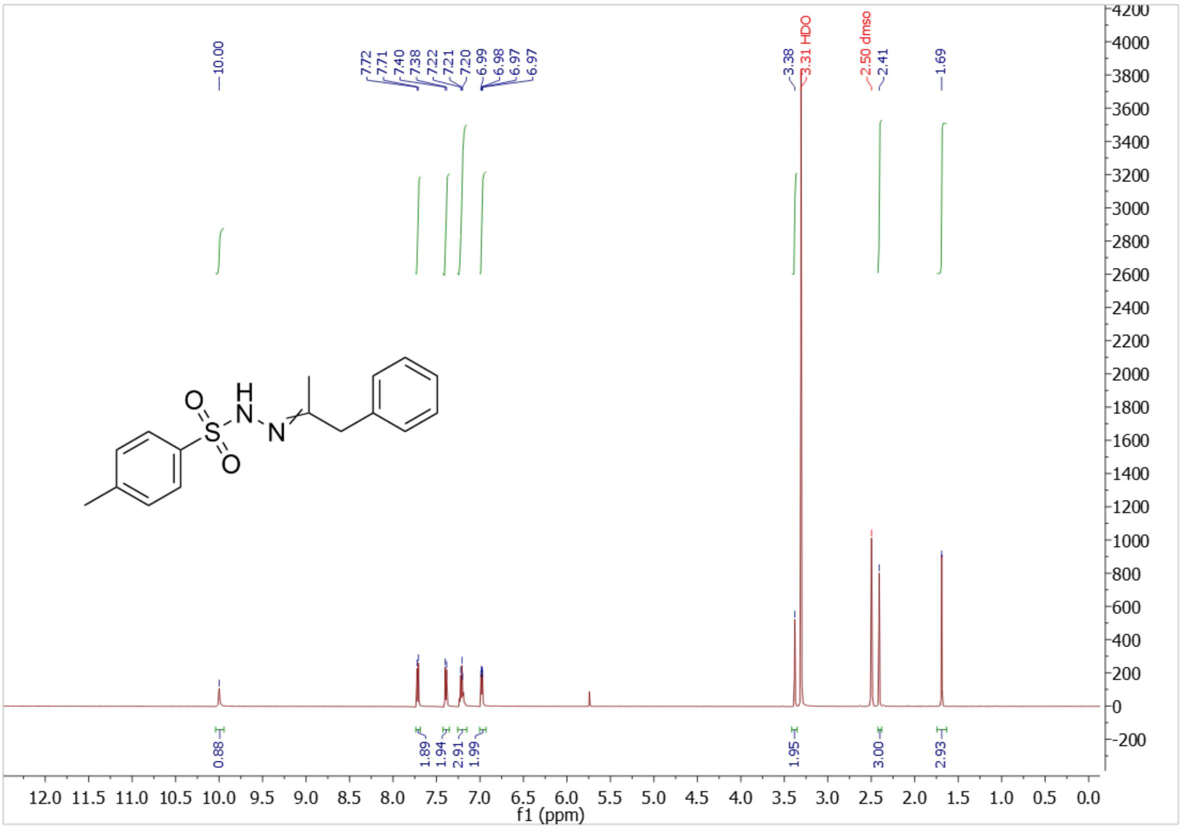

N′-(1-(3-aminophenyl)ethylidene)-4-methylbenzenesulfonohydrazide (**2e**)

In a round bottom flask 4-methylbenzenesulfonohydrazide (1.38 g; 7.40 mmol) and 3-aminoacetophenone (1.00 g; 7.40 mmol) were dissolved in 15 mL methanol. The resulting mixture was stirred at 60 °C for 4 hours. After completion of the reaction the mixture was cooled down, and the product was collected by filtration.

Yield: 1.79 g (80%); white solid; m.p.: 171 °C; ^{1}H NMR (300 MHz, DMSO-d_6) δ 10.35 (s; 1H), 7.83 (s; 2H), 7.40 (s; 2H), 6.94 (d; J = 29.9 Hz; 2H), 6.67 (d; J = 55.5 Hz; 2H), 5.15 (s; 2H), 2.35 (s; 3H), 2.10 (s; 3H) ppm; ^{13}C NMR (75 MHz, DMSO-d_6) δ 154.30; 148.98; 143.71; 138.54; 136.77; 129.89; 129.20; 128.06; 115.60; 114.40; 111.67; 21.48; 14.82 ppm; HRMS (ESI/Q-TOF) m/z: [M+H]$^+$ Calcd. for $C_{15}H_{18}N_3O_2S$ 304.1119; found 304.1117.

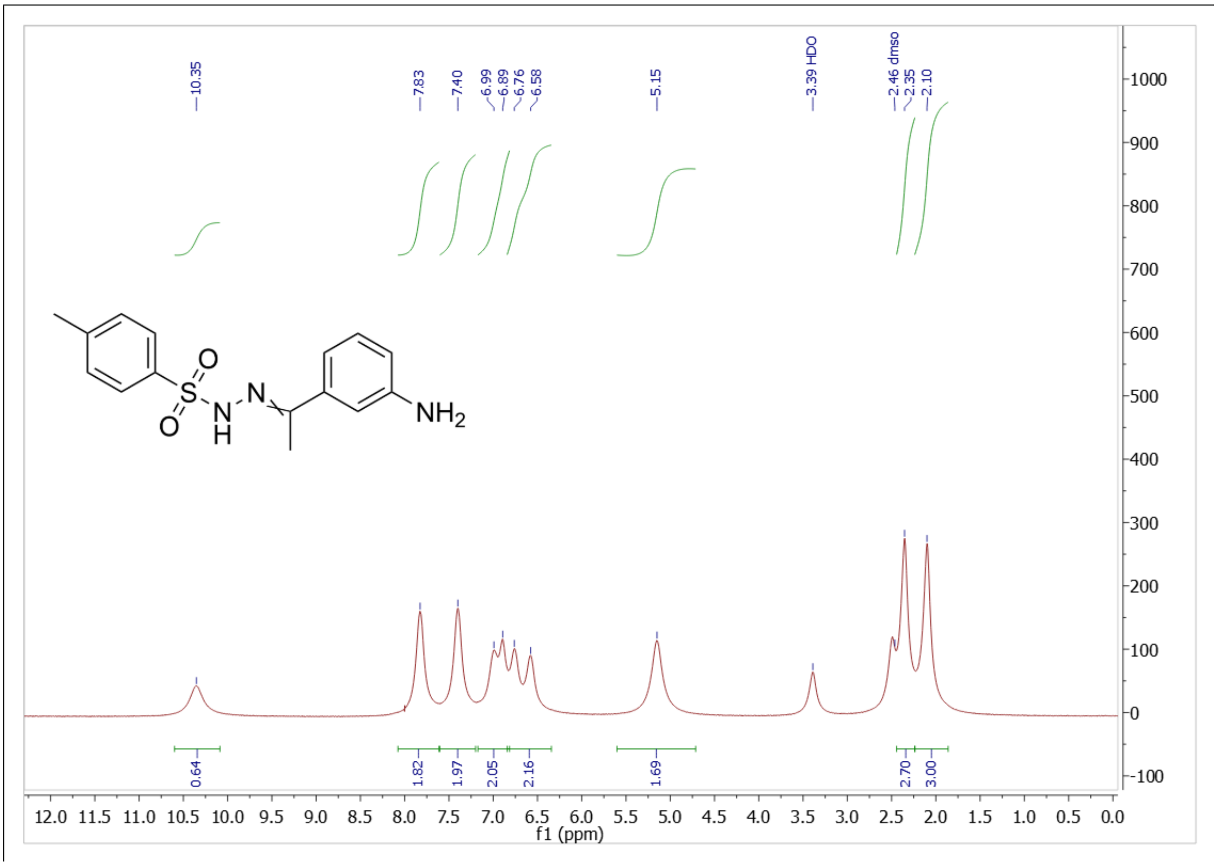

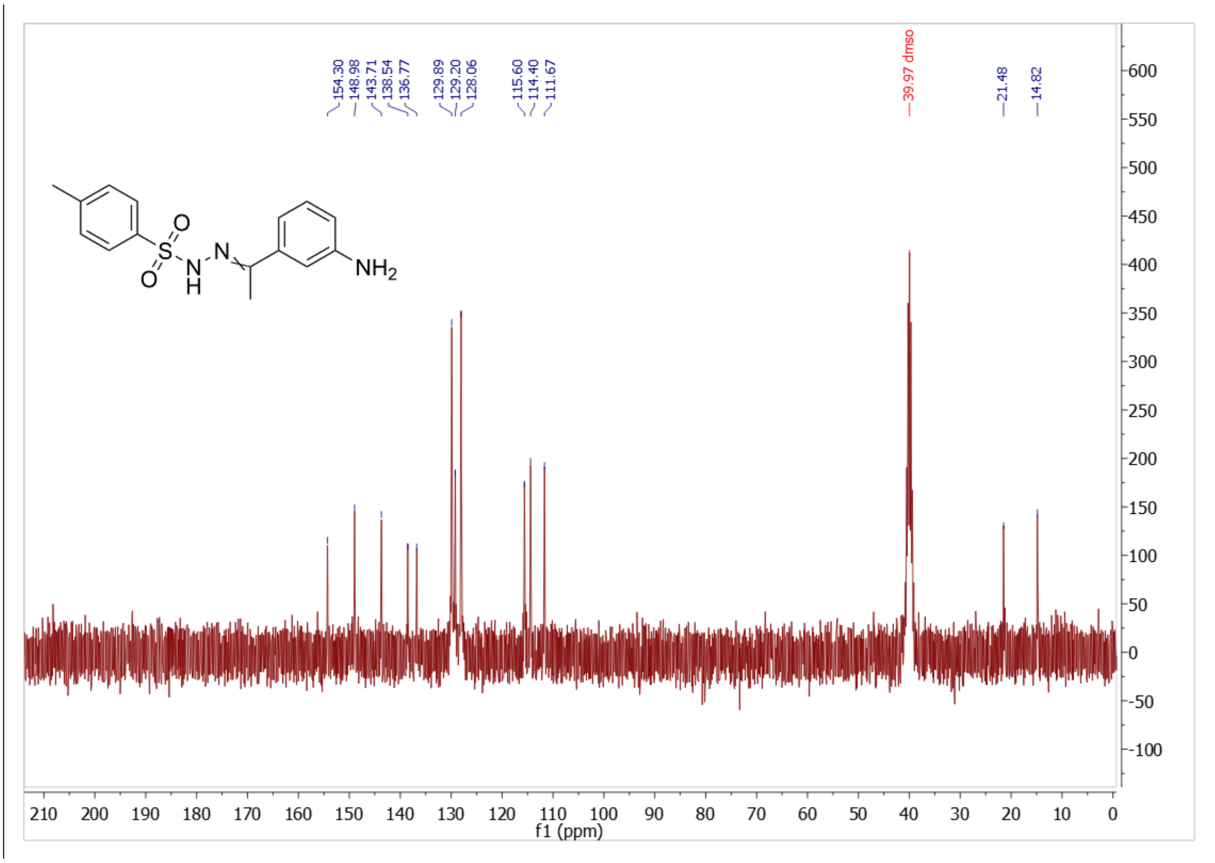

2-(1-(2-Tosylhydrazinylidene)ethyl)benzoic acid (**2f**)

In a round bottom flask 4-methylbenzenesulfonohydrazide (0.57 g; 3.05 mmol) and 2-acetylbenzoic acid (0.50 g; 3.05 mmol) were dissolved in 3 mL ethanol. The resulting mixture was stirred at room temperature for 1 week. After completion of the reaction the solvent was evaporated from the mixture, and the crude was purified by normal-phase flash chromatography.

Yield: 0.21 g (21%); white solid; m.p.: 75 °C; ^{1}H NMR (300 MHz, DMSO-d_6) δ 12.93 (s, 1H), 10.45 (s, 1H), 7.85 – 7.23 (m, 7H), 7.10 (m, J = 7.5, 1.5 Hz, 1H), 2.36 (s, 3H), 2.07 (s, 3H) ppm; ^{13}C NMR (75 MHz, DMSO-d_6) δ 168.35 , 157.07 , 143.48 , 140.98 , 137.03 , 132.01 , 130.95 , 130.14 , 129.83 , 129.32 , 129.01 , 127.74 , 21.48 , 19.34 ppm; HRMS (ESI/Q-TOF) m/z: [M+H]$^{+}$ Calcd. for $C_{16}H_{17}N_2O_4S$ 333.0909; found 330.0910.

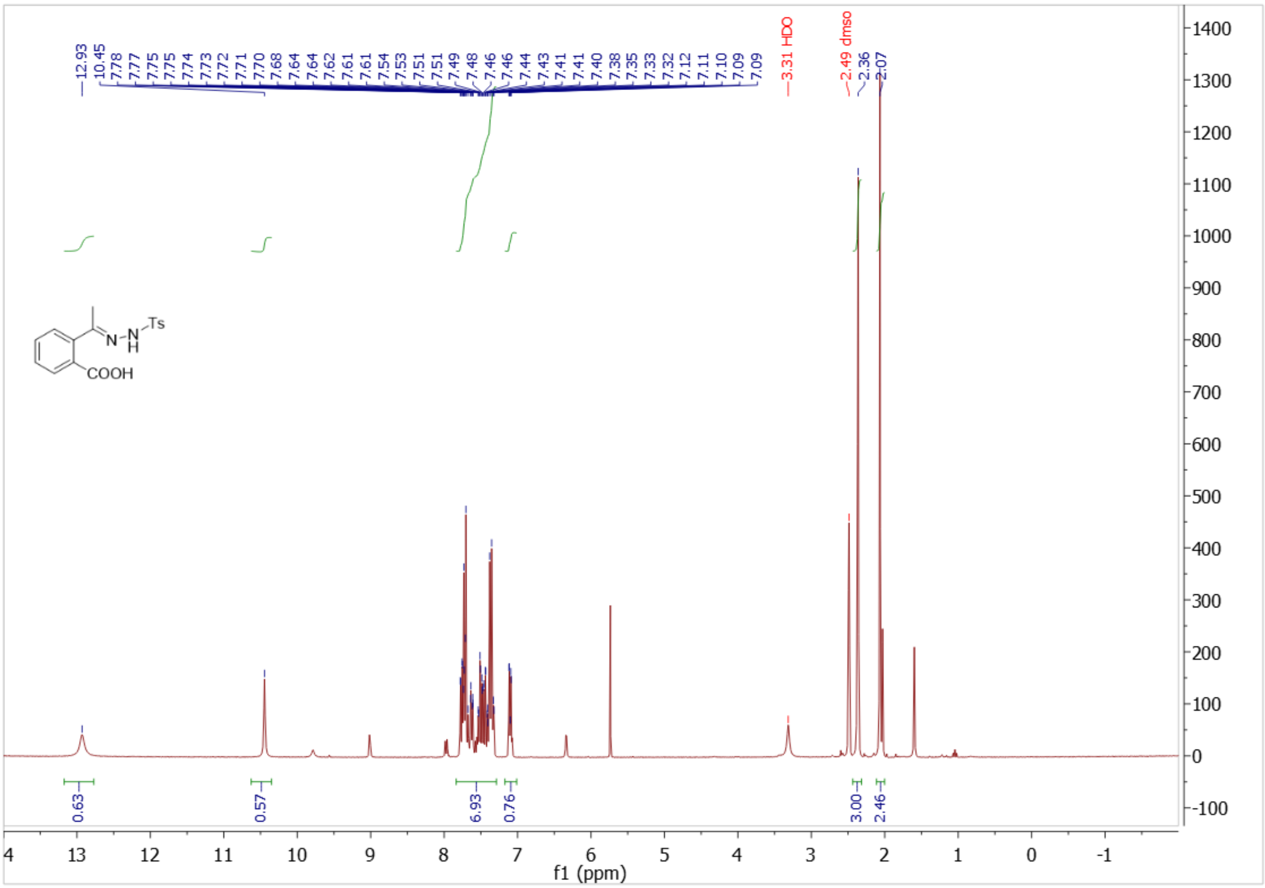

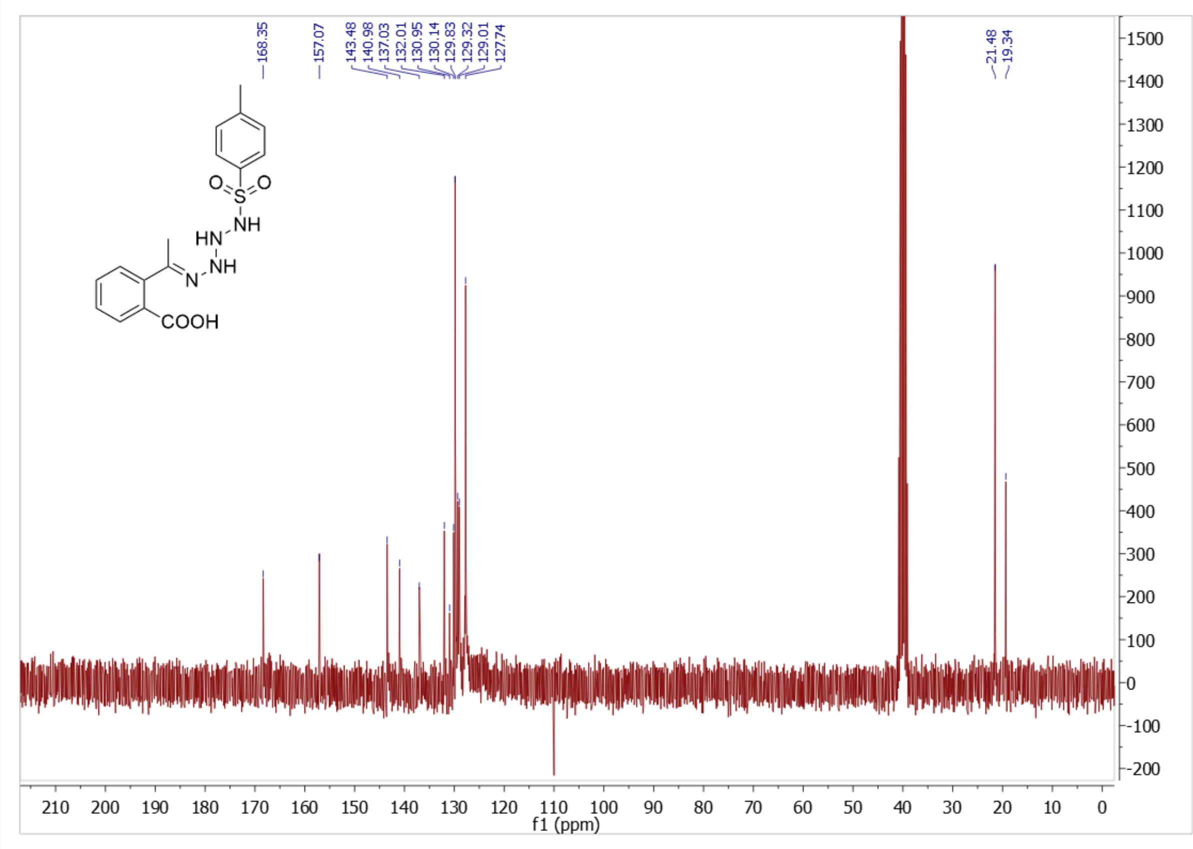

N'-(1-(3,5-bis(trifluoromethyl)phenyl)ethylidene)-4-methylbenzenesulfonohydrazide (**2g**)

In a round bottom flask 4-methylbenzenesulfonohydrazide (363 mg; 1.95 mmol) and 3',5'-bis(trifluoromethyl)acetophenone (352 µL; 1.95 mmol) were dissolved in 25 mL methanol and 5% 4-toluenesulfonic acid (18.5 mg; 0.097 mmol) was added. The resulting mixture was stirred at 60 °C for 2 hours. After completion of the reaction the mixture was cooled down, and the product was collected by filtration.

Yield: 670 mg (97%); white solid; m.p.: 143-147 °C; ^{1}H NMR: (500 MHz, $CDCl_3$): δ 8.03 (s, 2H), 7.92 (d, J = 8.3 Hz, 2H), 7.85 (s, 1H), 7.70 (s, 1H), 7.35 (d, J = 8.0 Hz, 2H), 2.43 (s, 3H), 2.20 (s, 3H) ppm.

4-Methyl-*N'*-(1-(thiophen-3-yl)ethylidene)benzenesulfonohydrazide (**2h**)

In a round bottom flask 4-methylbenzenesulfonohydrazide (1.64 g; 8.80 mmol) and 1-(thiophen-3-yl)ethan-1-one (1.11 g; 8.80 mmol) were dissolved in 10 mL ethanol. The resulting mixture was stirred at 60 °C for 4 hours. After completion of the reaction the mixture was cooled down, and the product was collected by filtration.

Yield: 2.33 g (90%); white solid; m.p.: 178 °C; ^{1}H NMR (500 MHz, DMSO-d_6) δ 10.32 (s; 1H), 7.84 – 7.71 (m; 3H), 7.50 (dd; J = 5.1; 2.9 Hz; 1H), 7.40 (d; J = 8.0 Hz; 2H), 7.30 (dd; J = 5.1;

1.2 Hz; 1H), 2.36 (s; 3H), 2.14 (s; 3H) ppm; ^{13}C NMR (75 MHz, DMSO-d_6) δ 150.59; 143.76; 140.92; 136.58; 129.85; 128.13; 127.25; 126.04; 125.66; 21.47; 15.27 ppm; HRMS (ESI/Q-TOF) m/z: $[M+H]^+$ Calcd. for $C_{13}H_{15}N_2O_2S_2$ 295.0574; found 295.0570.

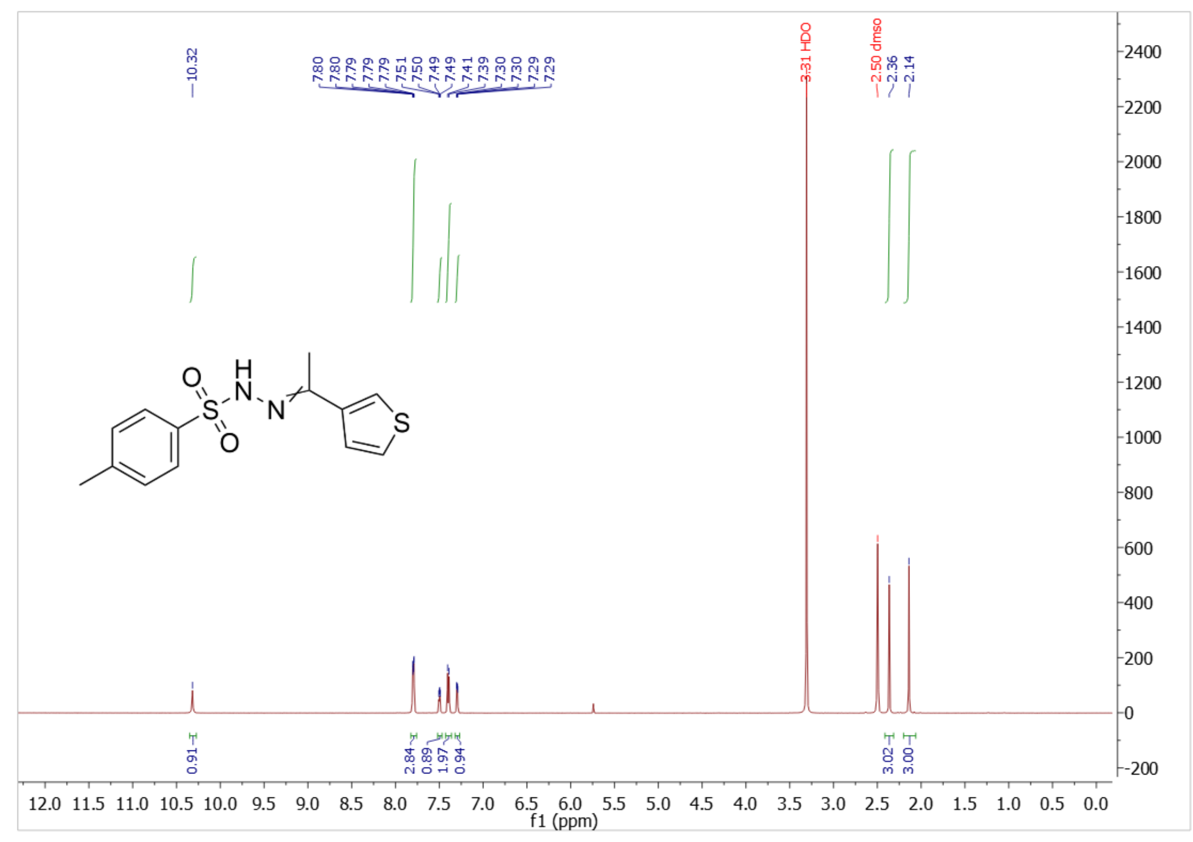

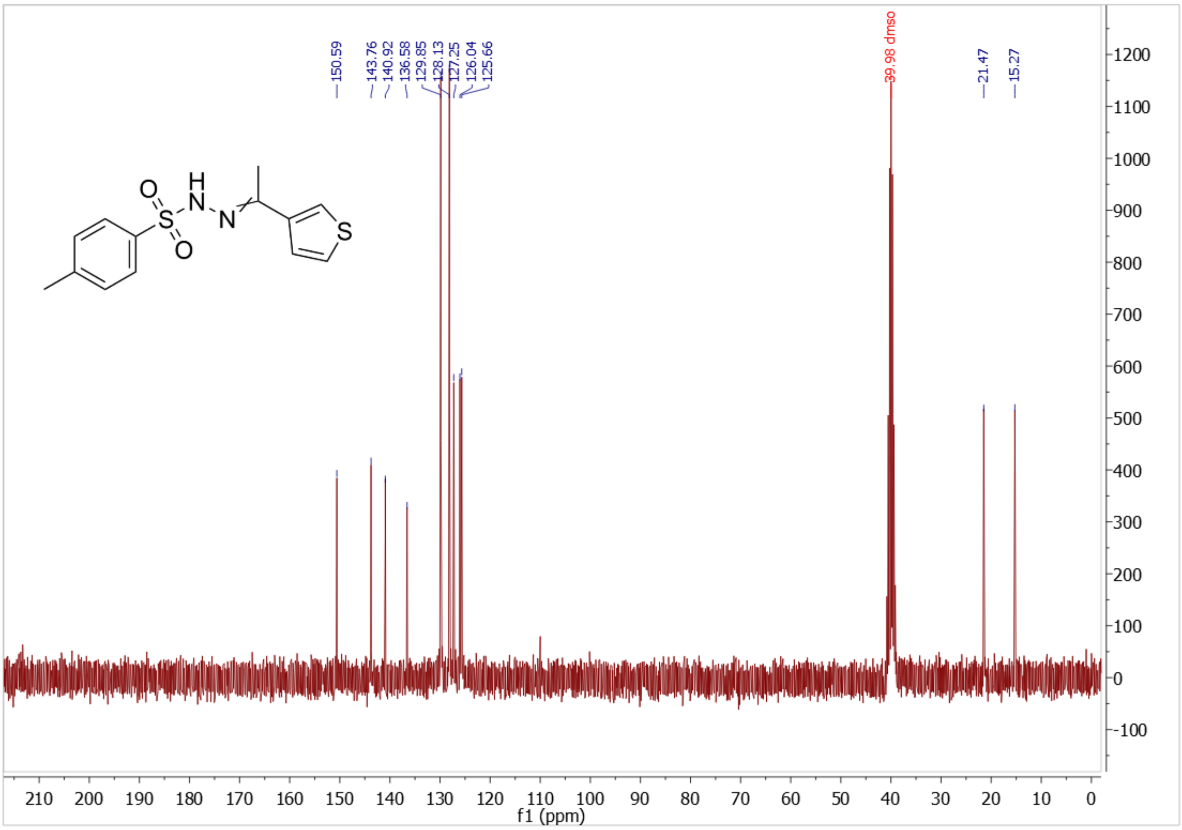

4-Methyl-*N'*-(1-(pyrazin-2-yl)ethylidene)benzenesulfonohydrazide (**2i**)

In a round bottom flask 4-methylbenzenesulfonohydrazide (1.53 g; 8.19 mmol) and 1-(pyrazin-2-yl)ethan-1-one (1.00 g; 8.19 mmol) were dissolved in 8 mL ethanol. The resulting mixture was stirred at room temperature for 2 days. After completion of the reaction the solvent was evaporated.

Yield: 1.95 g (82%); white solid; m.p.: 180 °C; ^{1}H NMR (300 MHz, DMSO-d_6) δ 11.05 (s, 1H), 8.96 (s, 1H), 8.58 (s, 2H), 7.87 (d, J = 8.0 Hz, 2H), 7.41 (d, J = 8.0 Hz, 2H), 2.34 (s, 3H), 2.24 (s, 3H) ppm; ^{13}C NMR (75 MHz, DMSO-d_6) δ 151.87; 150.18; 144.74; 144.11; 143.74; 142.16; 136.37; 130.07; 128.05; 21.45; 12.79 ppm; HRMS (ESI/Q-TOF) m/z: $[M+H]^+$ Calcd. for $C_{13}H_{15}N_4O_2S$ 291.0915; found 291.0913.

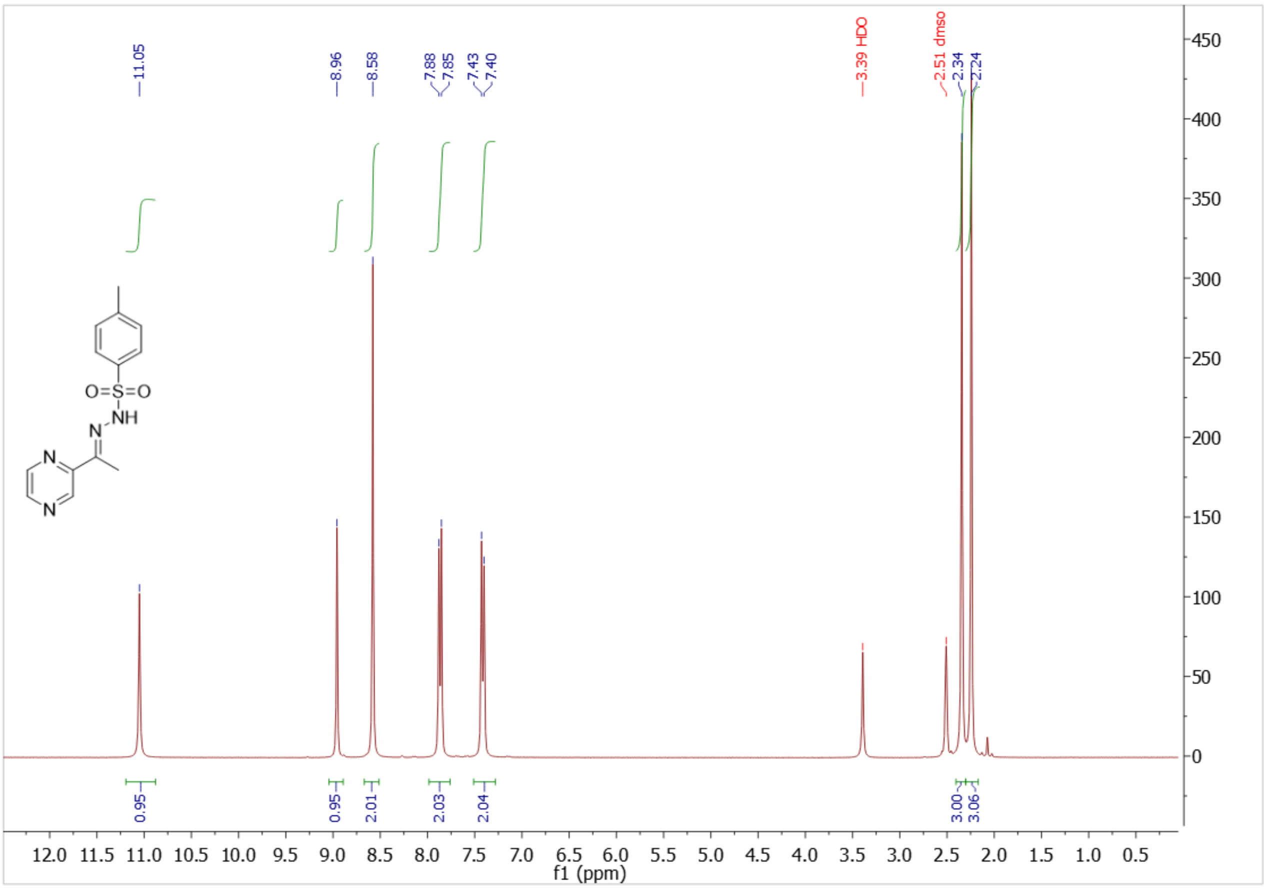
11.05
8.96
8.58
7.88
7.85
7.43
7.40
3.39 HDO
2.51 dmso
2.34
2.24
0.95
0.95
2.01
2.03
2.04
3.00
3.06
f1 (ppm)

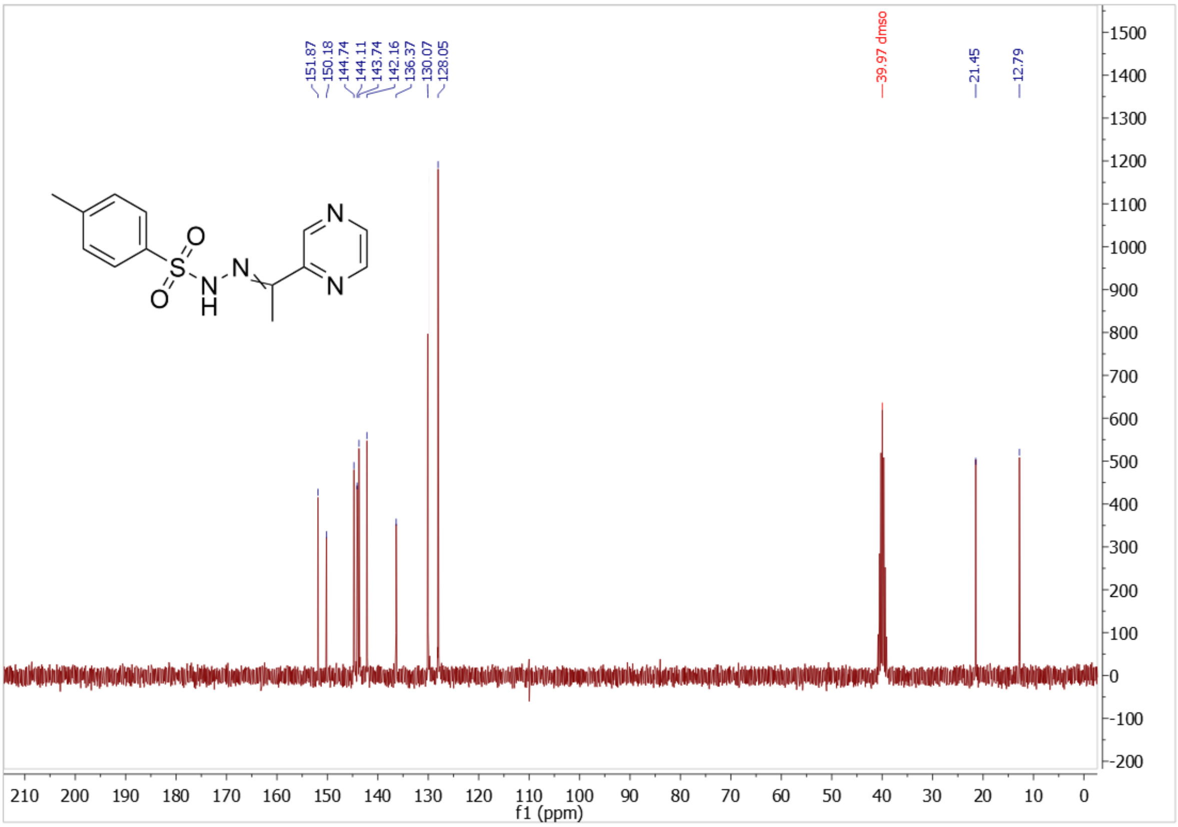
151.87
150.18
144.74
144.11
143.74
142.16
136.37
130.07
128.05
39.97 dmso
21.45
12.79
f1 (ppm)

N′-(1-(1H-benzo[d]imidazol-2-yl)ethylidene)-4-methylbenzenesulfonohydrazide (**2j**)

In a round bottom flask 4-methylbenzenesulfonohydrazide (0.12 g; 0.62 mmol) and 1-(1H-benzo[d]imidazol-2-yl)ehan-1-one (0.10 g; 0.62 mmol) were dissolved in 5 mL ethanol. The resulting mixture was stirred at 60 °C for 4 hours. After completion of the reaction the mixture was cooled down, and the product was collected by filtration. The crude was purified by normal-phase flash chromatography.

Yield: 0.11 g (52%); brown solid; m.p.: 167 °C; ^{1}H NMR (500 MHz, DMSO-d_6) δ 12.32 (s, 1H), 10.89 (s, 1H), 7.97 (d, J = 8.1 Hz, 2H), 7.62 (d, J = 8.0 Hz, 1H), 7.52 (d, J = 8.0 Hz, 1H), 7.39 (d, J = 8.1 Hz, 2H), 7.23 (t, J = 7.8 Hz, 1H), 7.17 (t, J = 7.8 Hz, 1H), 2.35 (s, 3H), 2.31 (s, 3H) ppm; ^{13}C NMR (126 MHz, DMSO-d_6) δ 149.78; 146.14; 144.01; 143.48; 136.57; 135.36; 129.95; 128.11; 124.12; 122.13; 119.81; 112.42; 21.44; 13.71 ppm; HRMS (ESI/Q-TOF) m/z: $[M+H]^+$ Calcd. for $C_{16}H_{17}N_4O_2S$ 329.1072; found 329.1070.

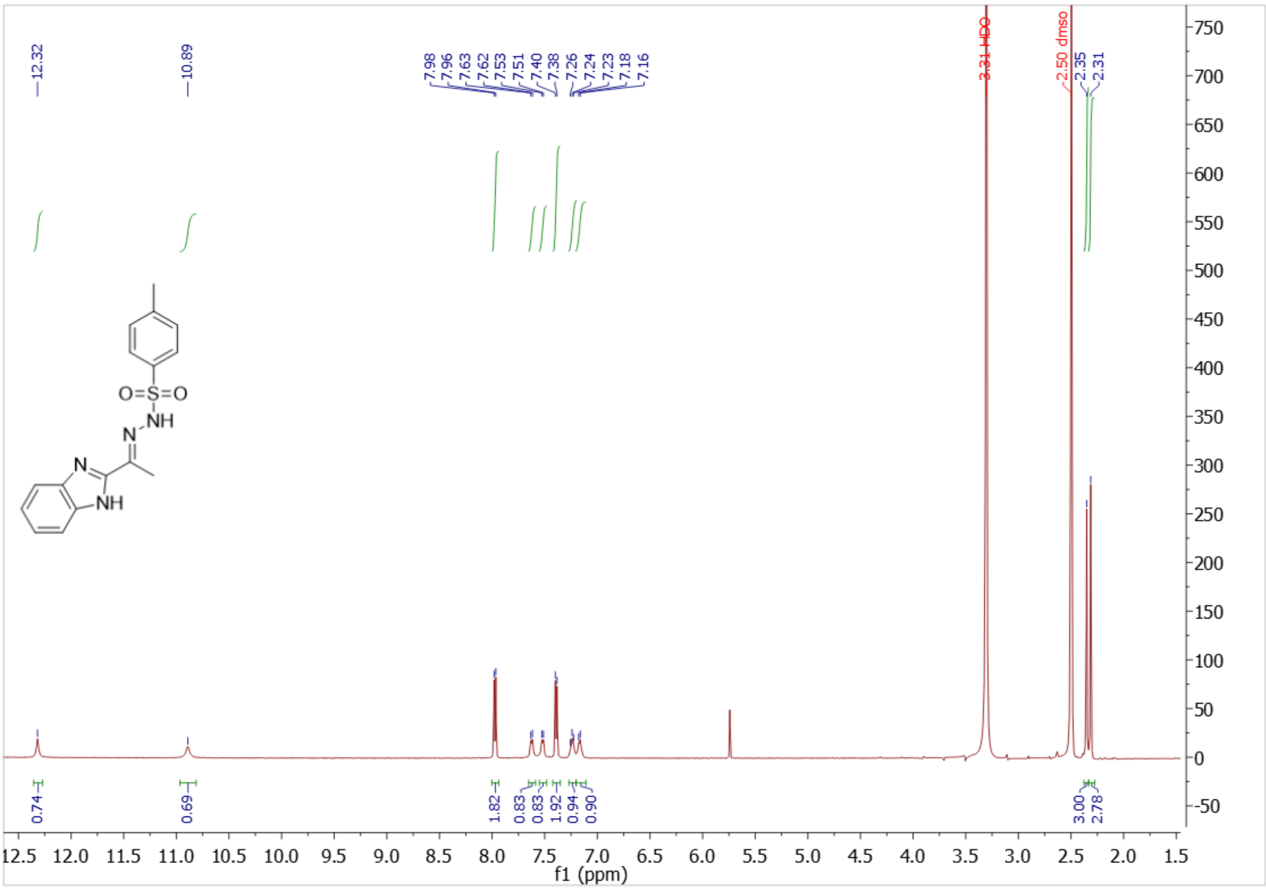

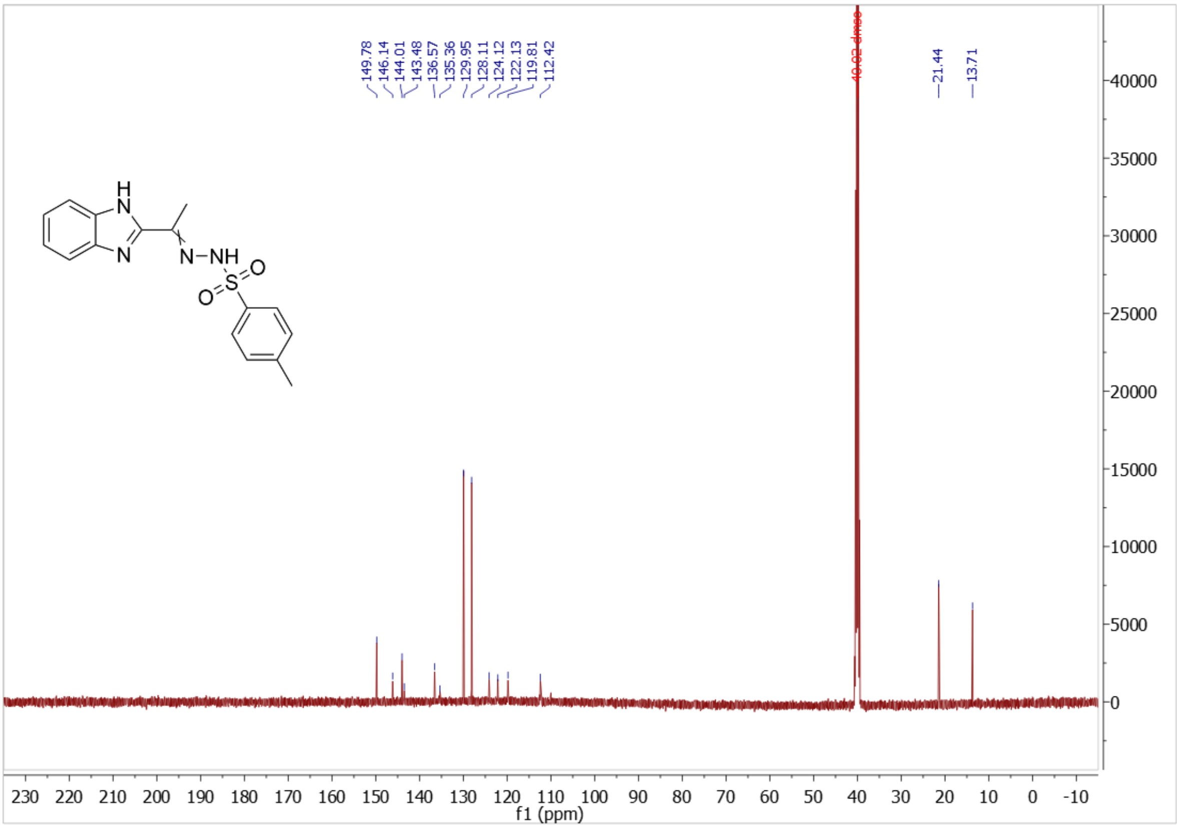

N′-(diphenylmethylene)-4-methylbenzenesulfonohydrazide (**2k**)

In a round bottom flask 4-methylbenzenesulfonohydrazide (1.02 g; 5.49 mmol) and benzophenone (1.00 g; 5.49 mmol) and catalytic amount of p-toluenesulfonic acid were dissolved in 30 mL ethanol. The resulting mixture was stirred at 70 °C for 3 hours. After completion of the reaction the mixture was cooled down, and the product was collected by filtration.

Yield: 1.21 g (63%); white solid; ^{1}H NMR (300 MHz, DMSO-d_6) δ 10.44 (s; 1H), 7.83 (d; J = 8.3 Hz, 2H), 7.58 – 7.50 (m; 3H), 7.43 (d; J = 8.1 Hz; 2H), 7.40 – 7.30 (m; 3H), 7.26 – 7.18 (m; 4H), 2.39 (s; 3H) ppm.

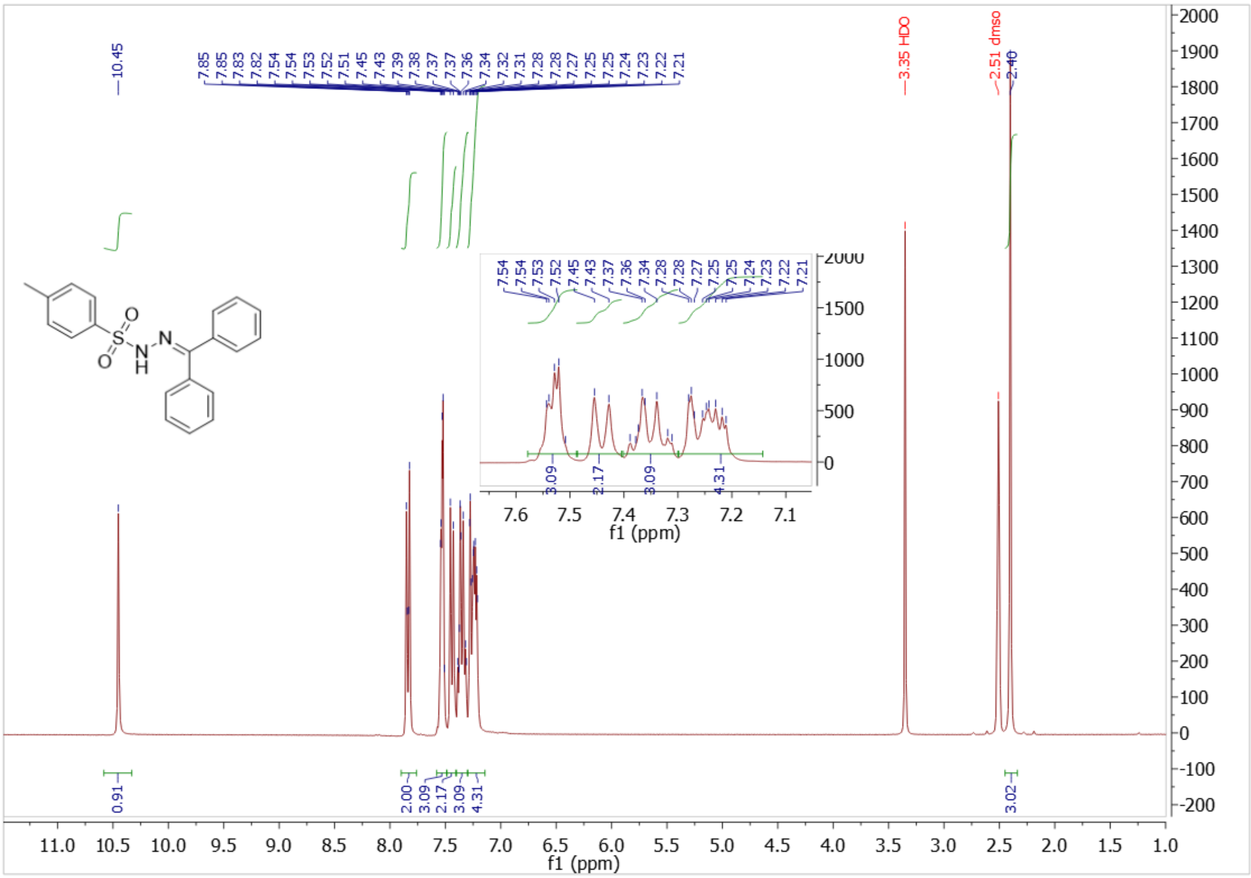

Methyl 2-phenyl-2-(2-tosylhydrazinylidene)acetate (**2l**)

In a round bottom flask 4-methylbenzenesulfonohydrazide (0.37 g; 2.00 mmol) and methyl 2-oxo-2-phenylacetate (0.33 g; 2.00 mmol; 0.29 mL) were dissolved in 2 mL ethanol. The resulting mixture was stirred at 70 °C for 2 hours. After completion of the reaction the mixture was cooled down, and the product was collected by filtration.

Yield: 0.48 g (71%); white solid; ^{1}H NMR (300 MHz, DMSO-d_6) δ 11.65 (s; 1H), 7.82 (d; J = 8.0 Hz; 2H), 7.63 – 7.22 (m; 7H), 3.88 (s; 3H), 2.39 (s; 3H) ppm.

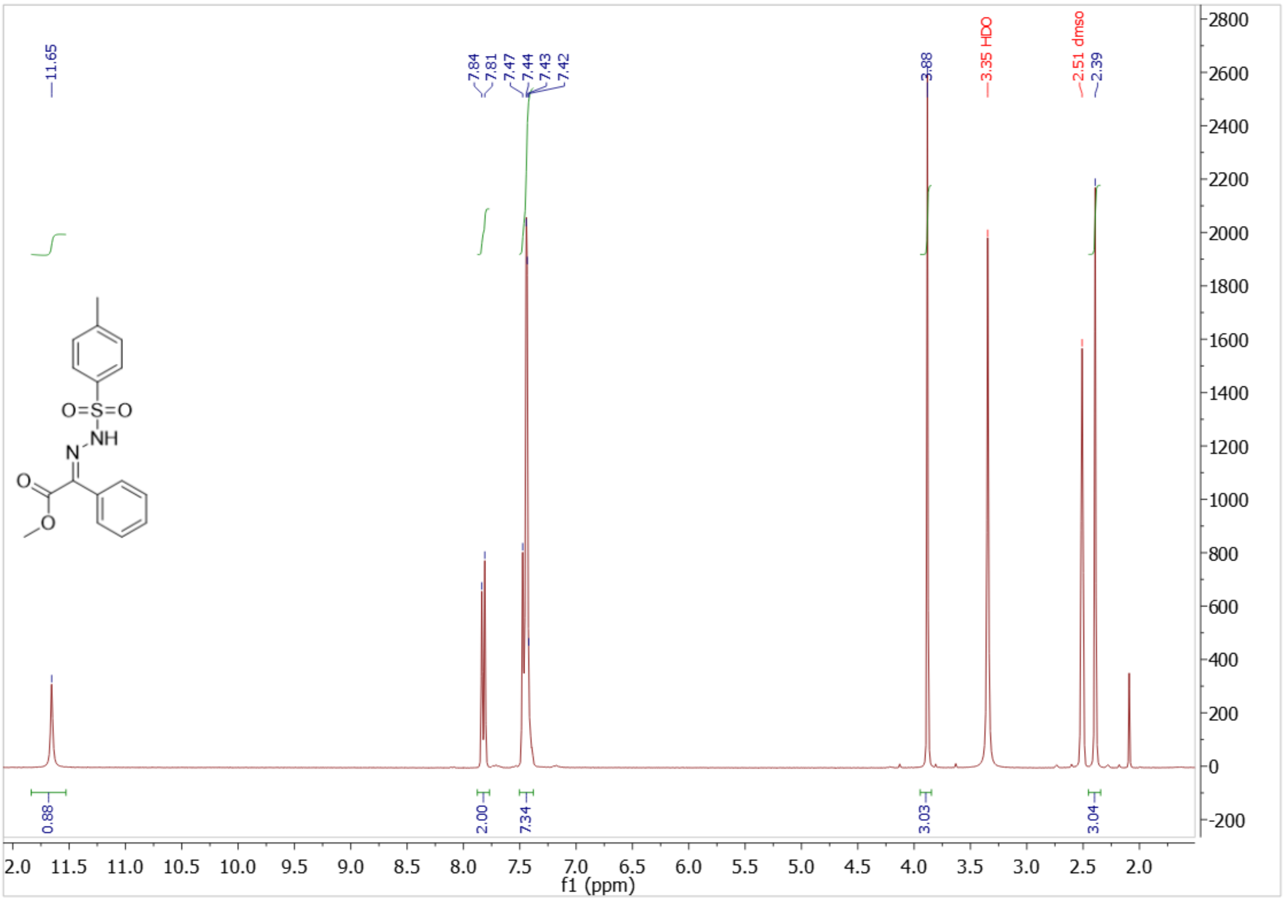

N'-((4-fluorophenyl)(thiophen-3-yl)methylene)-4-methylbenzenesulfonohydrazide (**2m**)

In a round bottom flask 4-methylbenzenesulfonohydrazide (1.81 g; 9.70 mmol) and (4-fluorophenyl)(thiophen-3-yl)methanone (2.00 g; 9.70 mmol) were dissolved in 10 mL ethanol. The resulting mixture was stirred at 80 °C for 6 hours. After completion of the reaction the mixture was cooled down, and the product was collected by filtration. The filtrate was purified by normal phase flash chromatography.

Yield: 3.10 g (85%); pale yellow solid; m.p.: 110-112 °C; ^{1}H NMR (300 MHz, DMSO-d_6) δ 10.47 (s, 1H), 7.99 – 7.67 (m, 2H), 7.57 (d, J = 5.1 Hz, 1H), 7.37 (dd, J = 14.4, 7.8 Hz, 5H), 7.21 (d, J = 8.1 Hz, 1H), 6.97 (d, J = 4.3 Hz, 1H), 6.69 (d, J = 3.7 Hz, 1H), 2.37 (s, 3H) ppm; ^{13}C NMR (75 MHz, DMSO-d_6) δ 163.15 (d, J = 246.5 Hz), 149.92 , 143.83 , 142.70 , 136.34 , 131.60 (d, J = 8.8 Hz), 129.80 , 129.61 , 128.66 (d, J = 3.3 Hz), 128.33 , 128.18 , 128.01 , 116.27 (d, J = 21.9 Hz), 62.47 , 21.48 ppm; HRMS (ESI/Q-TOF) m/z: [M+H]$^+$ Calcd. for $C_{18}H_{16}FN_2O_2S_2$ 375.0637; found 375.0641.

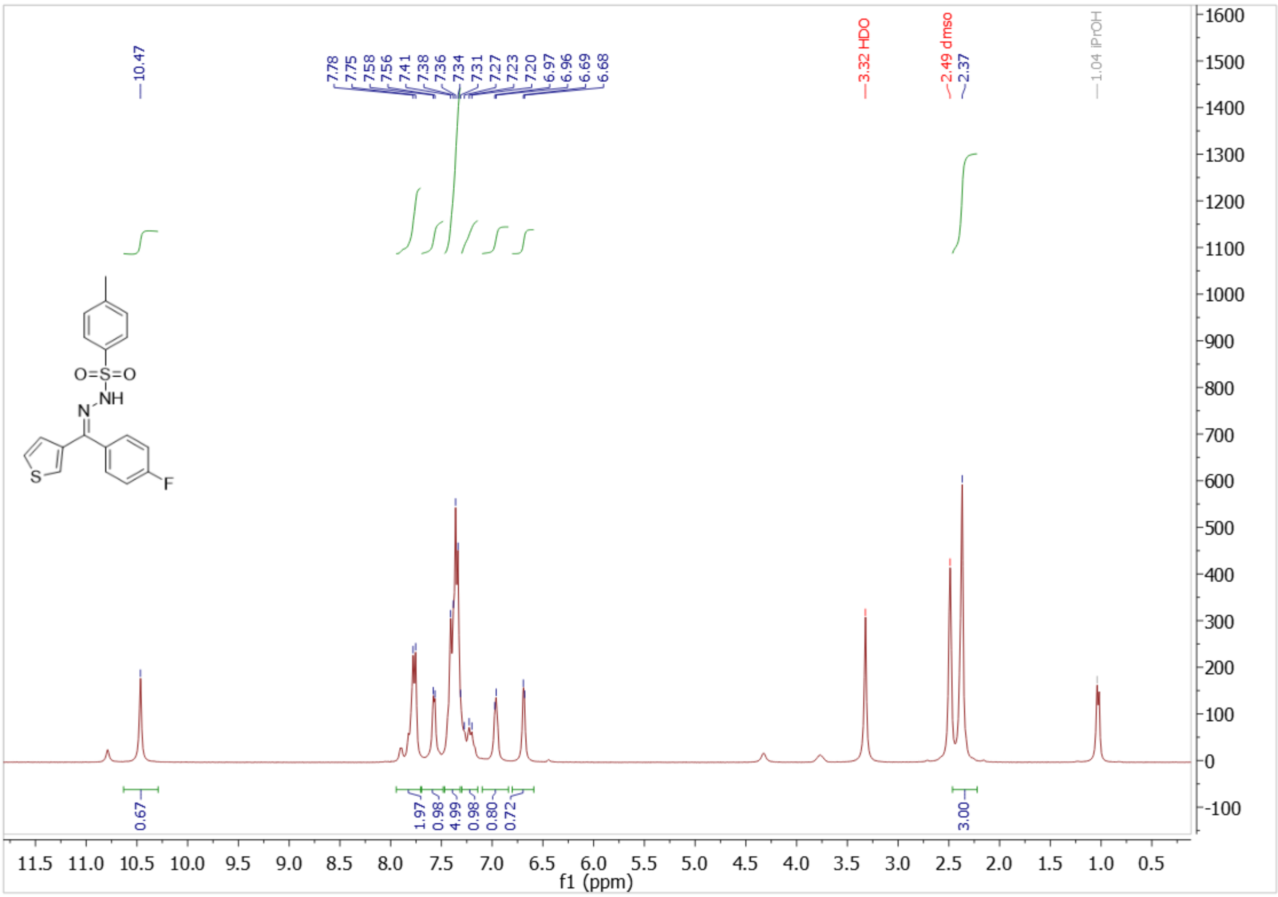

10.47
7.78 7.75 7.58 7.56 7.41 7.38 7.36 7.34 7.31 7.27 7.23 7.20 6.97 6.96 6.69 6.68
3.32 HDO
2.49 dmso
2.37
1.04 iPrOH
0.67
1.97 0.98 4.99 0.98 0.80 0.72
3.00
f1 (ppm)

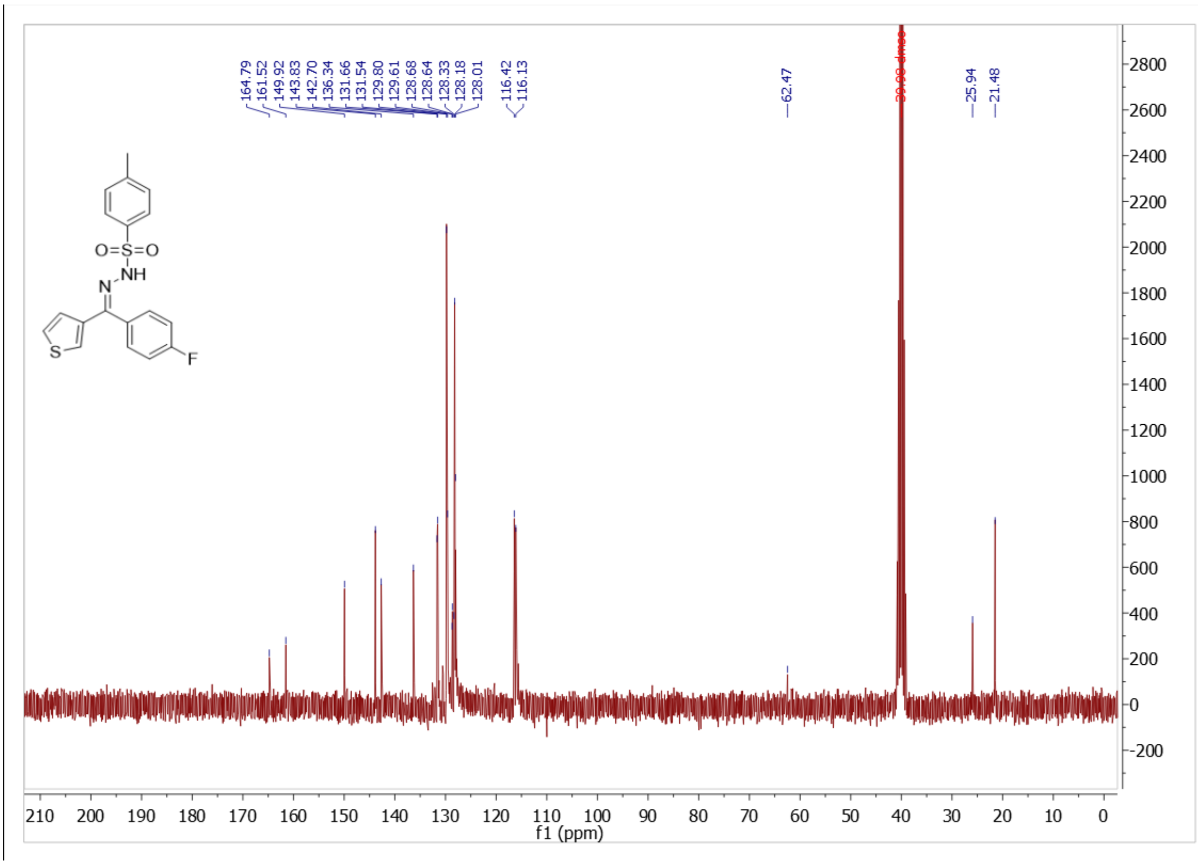

164.79 161.52 149.92 143.83 142.70 136.34 131.66 131.54 129.80 129.61 128.68 128.64 128.33 128.18 128.01 116.42 116.13
62.47
25.94
21.48
f1 (ppm)

N'-((1H-benzo[d]imidazol-2-yl)(phenyl)methylene)-4-methylbenzenesulfonohydrazide (**2n**)

In a round bottom flask 4-methylbenzenesulfonohydrazide (0.42 g; 2.25 mmol) and 1H-benzo[d]imidazol-2-yl)(phenyl)methanone (0.50 g; 2.25 mmol), and catalytic amount of p-toluenesulfonic acid were dissolved in 10 mL ethanol. The resulting mixture was stirred at room temperature for 1 week. After completion of the reaction the product was collected by filtration.

Yield: 0.10 g (12%); yellow solid; m.p.: 165 °C; ^{1}H NMR (300 MHz, DMSO-d_6) δ 13.19 (s; 1H), 7.84 (d; J = 8.2 Hz; 2H), 7.74 (d; J = 3.1 Hz; 1H), 7.72 (d; J = 3.1 Hz; 1H), 7.56 – 7.48 (m; 5H), 7.43 (d; J = 8.2 Hz; 2H), 7.38 (d; J = 3.2 Hz; 1H), 7.36 (d; J = 3.3 Hz; 1H), 2.37 (s; 3H) ppm; ^{13}C NMR (75 MHz, DMSO-d_6) δ 144.76; 144.37; 140.13; 137.63; 136.39; 135.53; 131.35; 130.34; 130.24; 129.23; 128.79; 128.52; 127.72; 126.00; 124.36; 116.53; 21.50 ppm; HRMS (ESI/Q-TOF) m/z: $[M+H]^+$ Calcd. for $C_{21}H_{19}N_4O_2S$ 391.1228; found 391.1224.

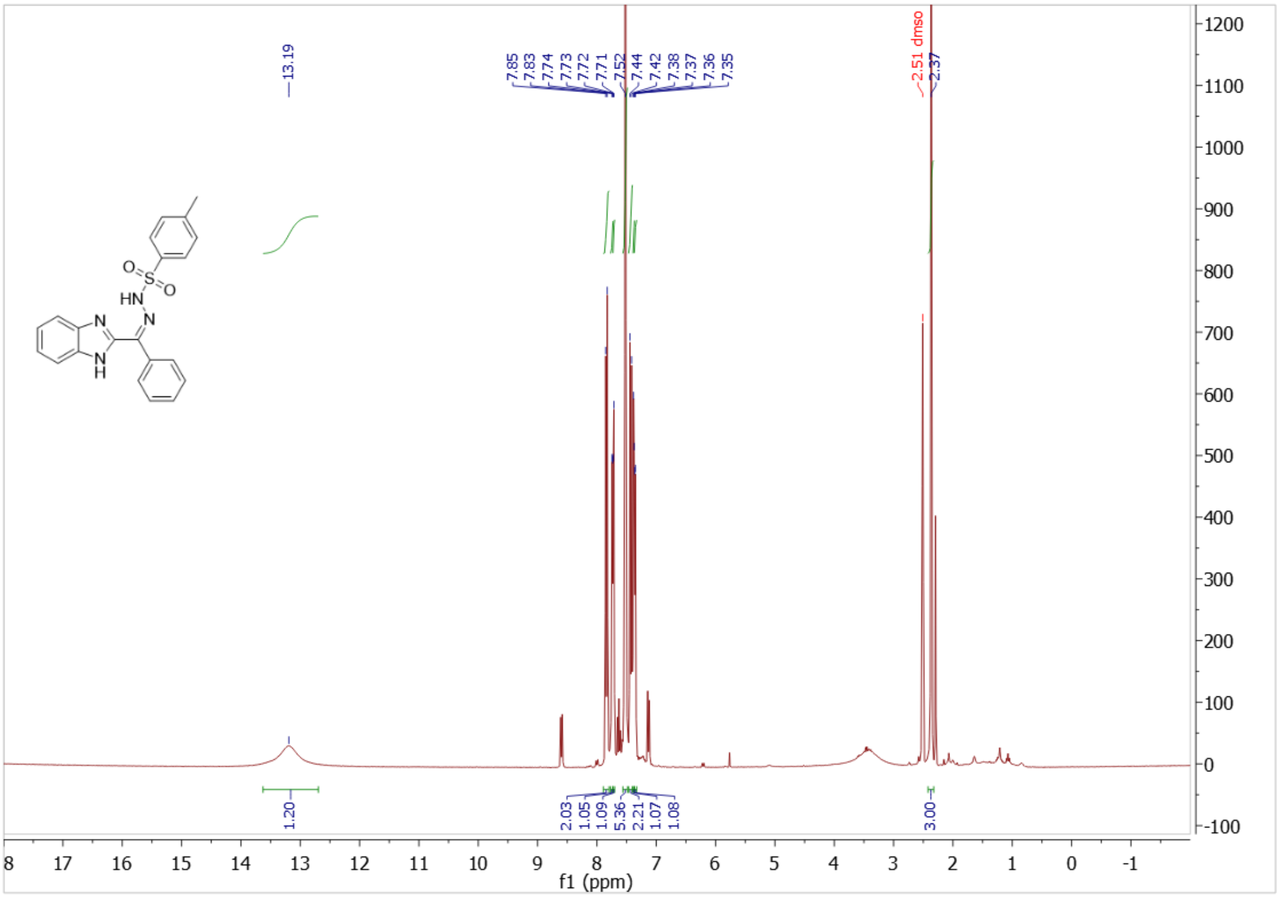

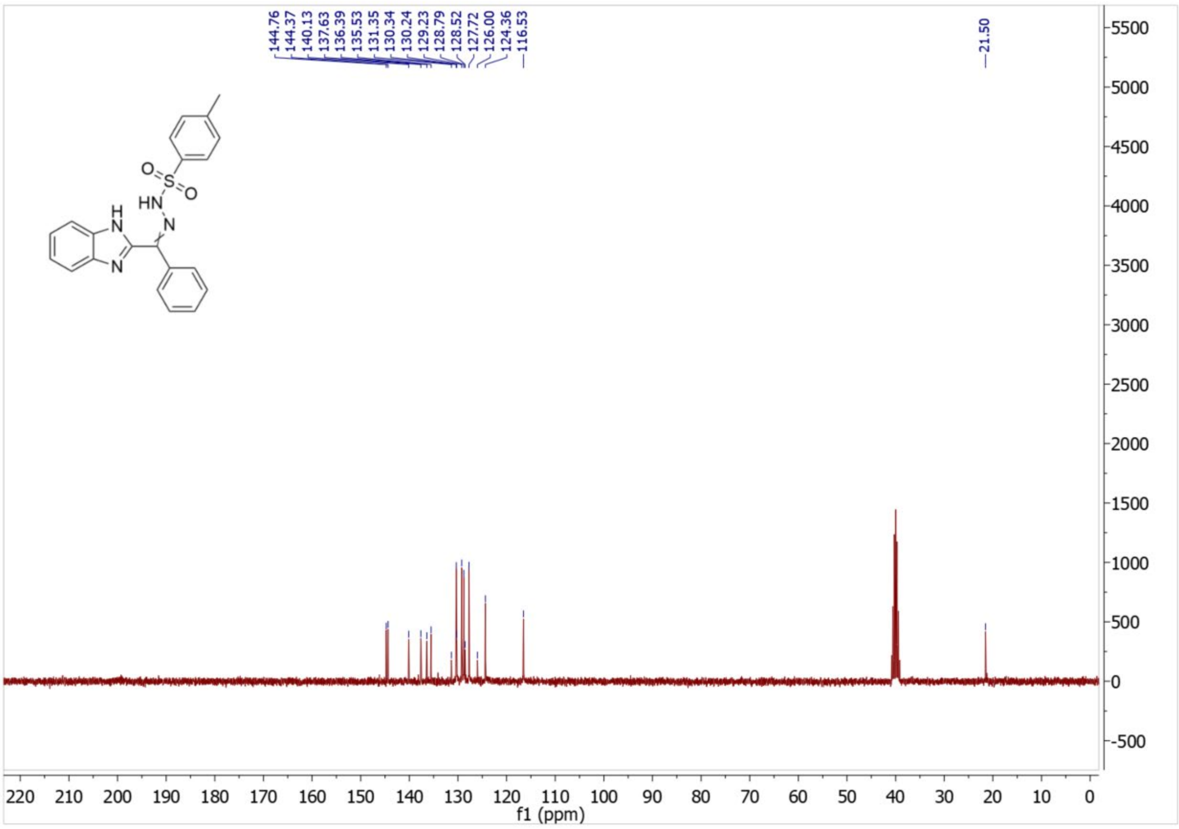

4-Methyl-*N′*-(2-oxoindolin-3-ylidene)benzenesulfonohydrazide (**2o**)

In a round bottom flask 4-methylbenzenesulfonohydrazide (1.27 g; 6.80 mmol) and indoline-2,3-dione (1.00 g; 6.80 mmol) were dissolved in 10 mL ethanol. The resulting mixture was stirred at room temperature for 1 day. After completion of the reaction the product was collected by filtration.

Yield: 1.93 g (90%); yellow solid; ^{1}H NMR (300 MHz, DMSO-d_6) δ 12.52 (s, 1H), 10.75 (s, 1H), 8.01 – 7.69 (m, 3H), 7.52 – 7.39 (m, 2H), 7.37 – 7.27 (m, 1H), 7.11 – 6.97 (m, 1H), 6.92 – 6.71 (m, 1H), 2.38 (s, 3H) ppm.

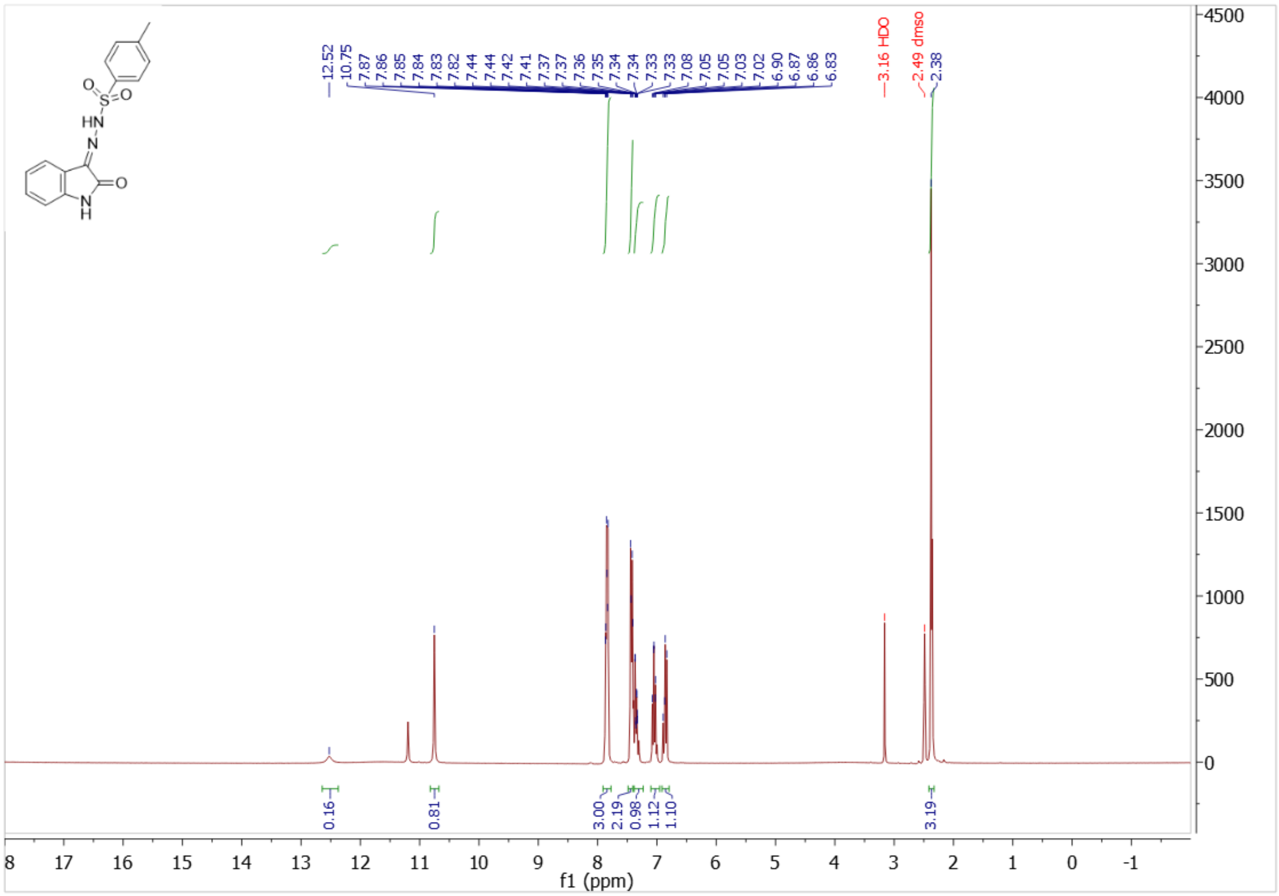

N'-(4,6-dichloro-2-oxoindolin-3-ylidene)-4-methylbenzenesulfonohydrazide (**2p**)

In a round bottom flask 4-methylbenzenesulfonohydrazide (0.09 g; 0.50 mmol) and 14-bromo-6-chloroindoline-2,3-dione (0.108 g; 0.50 mmol) were dissolved in 5 mL methanol. The resulting mixture was stirred at room temperature for 4 hours. After completion of the reaction the mixture was filtered.

Yield: 0.06 g (29%); yellow solid; m.p.: ^{1}H NMR (300 MHz, DMSO-d_6) δ 12.51 (s, 1H), 11.49 (s, 1H), 7.85 (d, J = 8.3 Hz, 2H), 7.42 (d, J = 8.1 Hz, 2H), 7.17 (d, J = 1.7 Hz, 1H), 6.86 (d, J = 1.7 Hz, 1H), 2.36 (s, 3H) ppm; ^{13}C NMR (75 MHz, DMSO-d_6) δ 161.57 , 145.02 , 144.96 , 136.19 , 135.37 , 135.08 , 130.19 , 128.75 , 128.33 , 123.31 , 115.90 , 110.38 , 21.51 ppm.

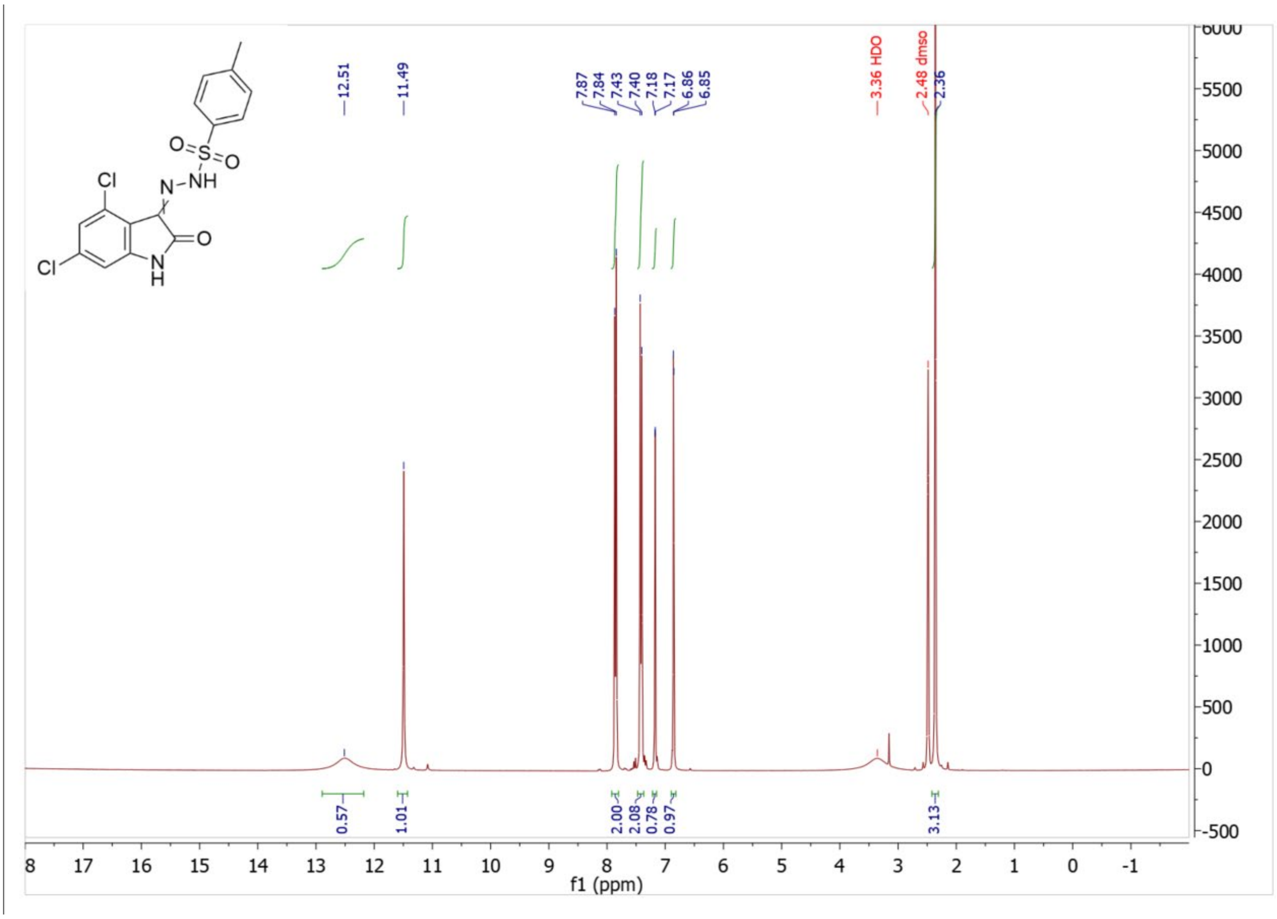
12.51
11.49
7.87
7.84
7.43
7.40
7.18
7.17
6.86
6.85
3.36 HDO
2.48 dmso
2.36
0.57
1.01
2.00
2.08
0.78
0.97
3.13
f1 (ppm)

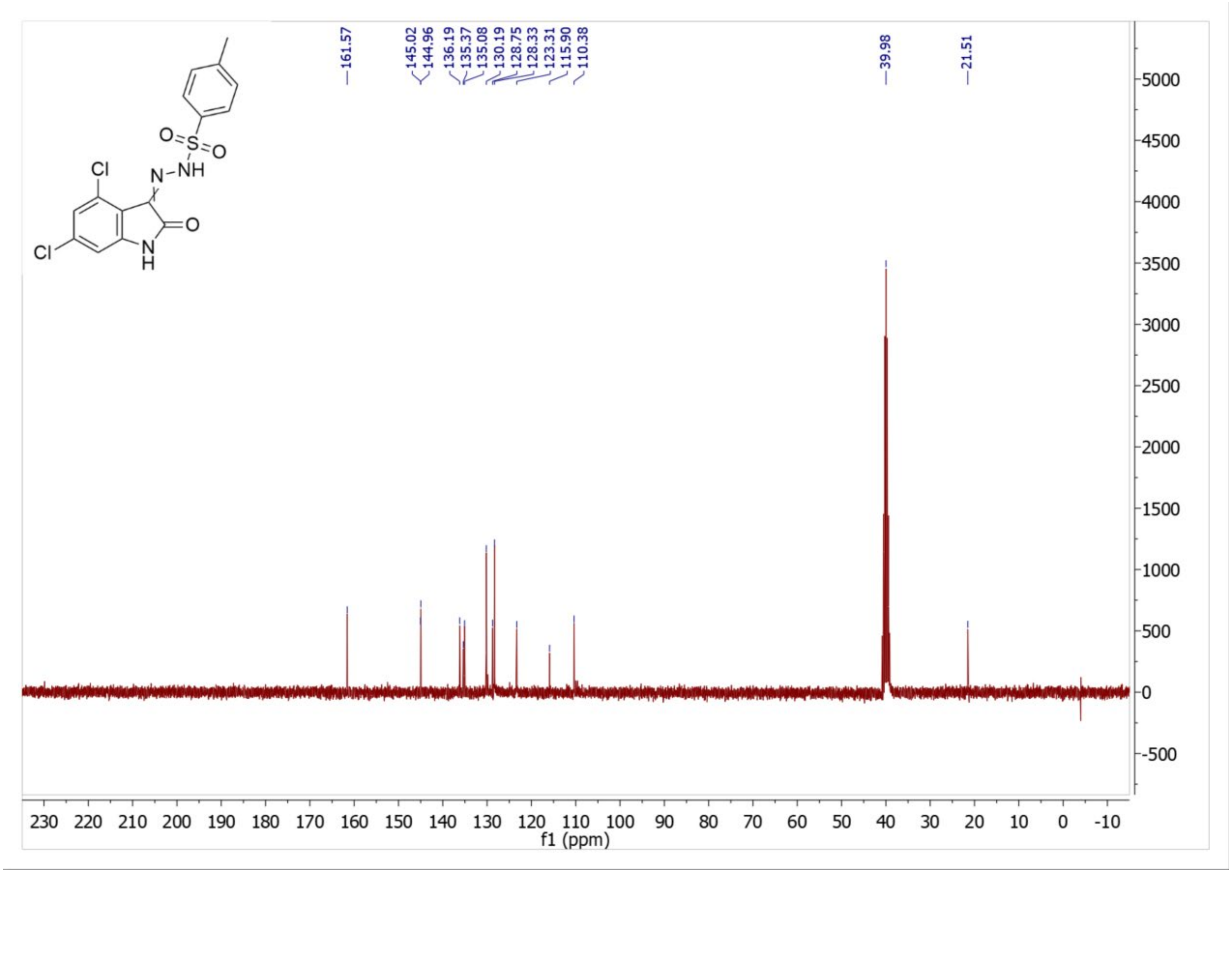
161.57
145.02
144.96
136.19
135.37
135.08
130.19
128.75
128.33
123.31
115.90
110.38
39.98
21.51
f1 (ppm)

4-Methyl-*N'*-(5-methyl-2-(propan-2-ylidene)cyclohexylidene)benzenesulfonohydrazide (**2q**)

In a round bottom flask 4-methylbenzenesulfonohydrazide (0.28 g; 1.50 mmol) and 5-methyl-2-(propan-2-ylidene)cyclohexane-1-one (0.23 g; 1.50 mmol; 247 μL) were dissolved in 10 mL methanol. The resulting mixture was stirred at 80 °C for 5 hours. After completion of the reaction the solvent was evaporated. The crude was purified by normal-phase flash chromatography.

Yield: 0.16 g (33%); brown gum; ^{1}H NMR (300 MHz, $CDCl_3$) δ 7.92 – 7.73 (m, 2H), 7.29 (d, *J* = 8.0 Hz, 2H), 2.58 – 2.45 (m, 1H), 2.42 (d, *J* = 3.0 Hz, 3H), 2.26 – 2.04 (m, 1H), 1.87 – 1.79 (m, 1H), 1.77 – 1.68 (m, 3H), 1.66 (s, 1H), 1.59 – 1.49 (m, 2H), 1.52 – 1.37 (m, 3H), 1.39 – 1.21 (m, 1H), 1.19 – 1.02 (m, 1H), 0.92 (d, *J* = 6.4 Hz, 2H), 0.89 – 0.72 (m, 1H).

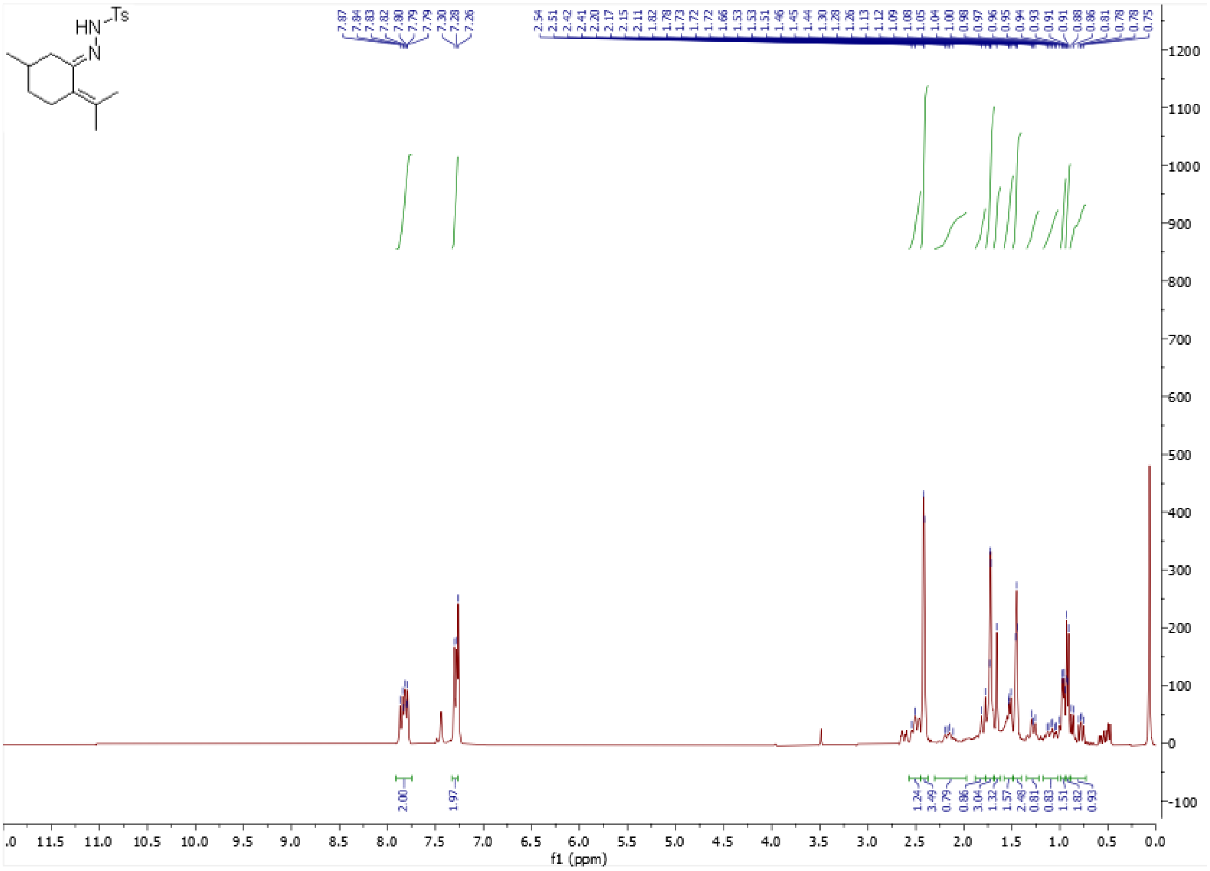

4-Methyl-2-tosylphthalazin-1(2H)-one (**2r**)

In a round bottom flask 4-methylbenzenesulfonohydrazide (0.57 g; 3.05 mmol) and 2-acetylbenzoic acid (0.50 g; 3.05 mmol) were dissolved in 3 mL ethanol. The resulting mixture was stirred at room temperature for 1 week. After completion of the reaction the solvent was evaporated from the mixture, and the crude was purified by normal-phase flash chromatography.

Yield: 0.34 g (35%); white solid; m.p.: 189 °C; ^{1}H NMR (300 MHz, DMSO-d_6) δ 8.17 (m, J = 8.0, 1.0 Hz, 1H), 8.06 – 7.97 (m, 3H), 7.94 (d, J = 8.4 Hz, 1H), 7.86 (m, J = 8.3, 6.3, 2.2 Hz, 1H), 7.56 – 7.27 (m, 2H), 2.60 (s, 3H), 2.38 (s, 3H) ppm; ^{13}C NMR (75 MHz, DMSO-d_6) δ 157.91 , 146.34 , 146.13 , 135.64 , 134.57 , 133.19 , 130.20 , 129.83 , 129.17 , 127.53 , 127.03 , 126.97 , 21.62 , 19.53 ppm; HRMS (ESI/Q-TOF) m/z: $[M+H]^+$ Calcd. for $C_{16}H_{15}N_2O_3S$ 315.0803; found 315.0804.

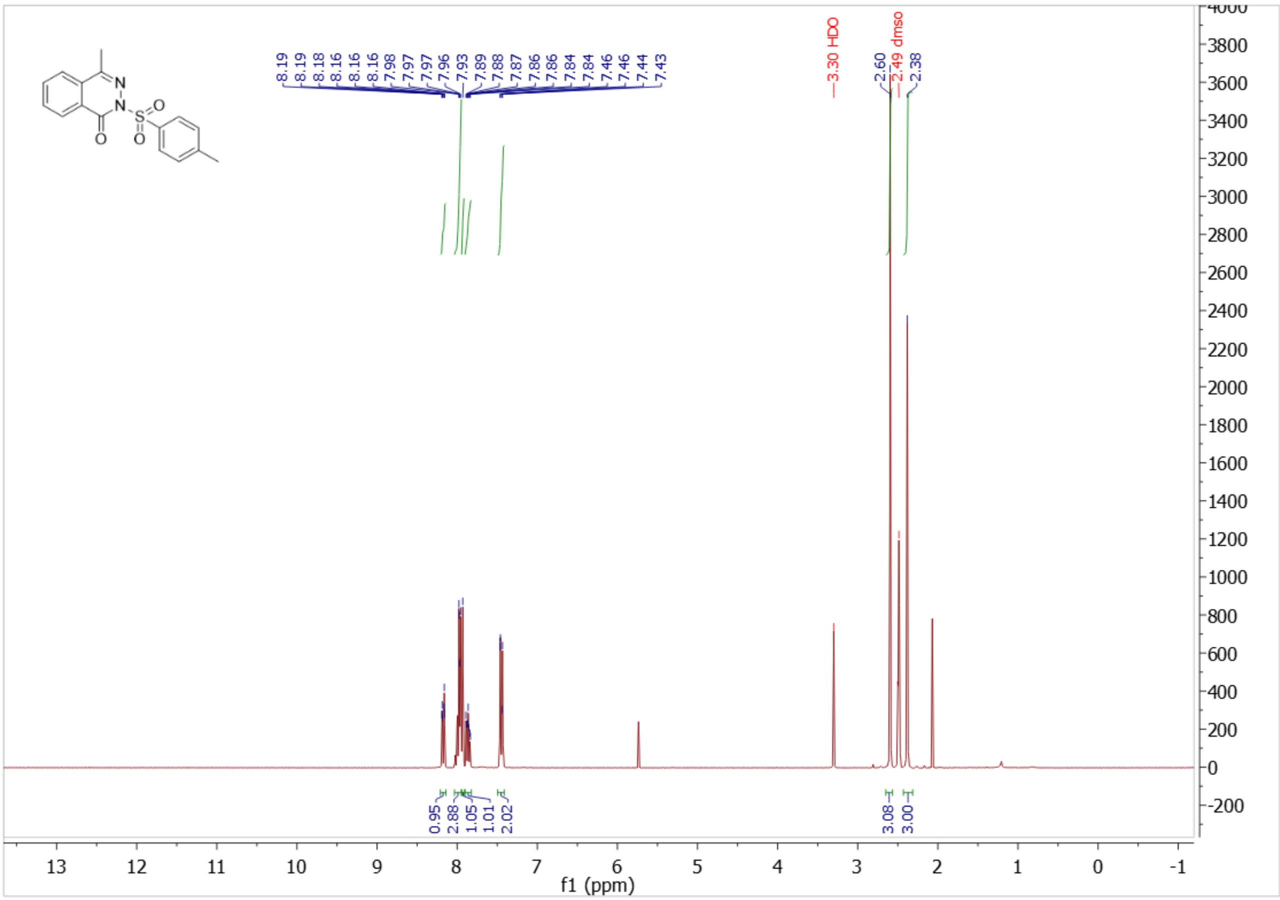

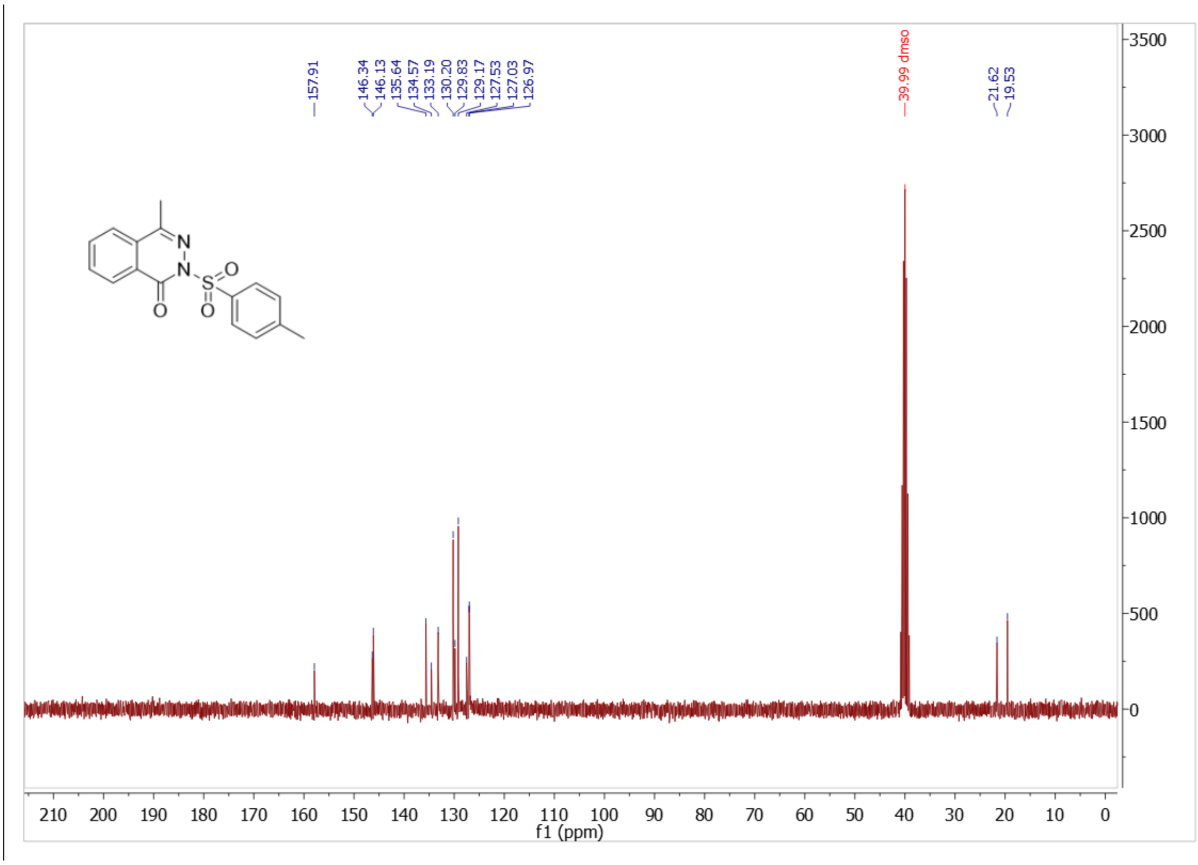

N'-(cyclohex-2-en-1-ylidene)-4-methylbenzenesulfonohydrazide (**2s**)

In a round bottom flask 4-methylbenzenesulfonohydrazide (0.28 g; 1.50 mmol) and cyclohex-2-en-1-one (0.14 g; 1.50 mmol; 145 µl) were dissolved in 50 mL ethanol. The resulting mixture was stirred at 80 °C for 2 hours. After completion of the reaction the solvent was evaporated. The crude was purified by normal-phase flash chromatography.

Yield: 0.06 g (15%); pale yellow solid; ^{1}H NMR (300 MHz, Chloroform-*d*) δ 7.85 (d, J = 8.3 Hz, 2H), 7.31 (d, 2H), 6.31 – 6.22 (m, 1H), 6.19 – 6.05 (m, 1H), 2.42 (s, 3H), 2.34 – 2.24 (m, 2H), 2.20 – 2.10 (m, 2H), 1.84 – 1.69 (m, 2H) ppm.

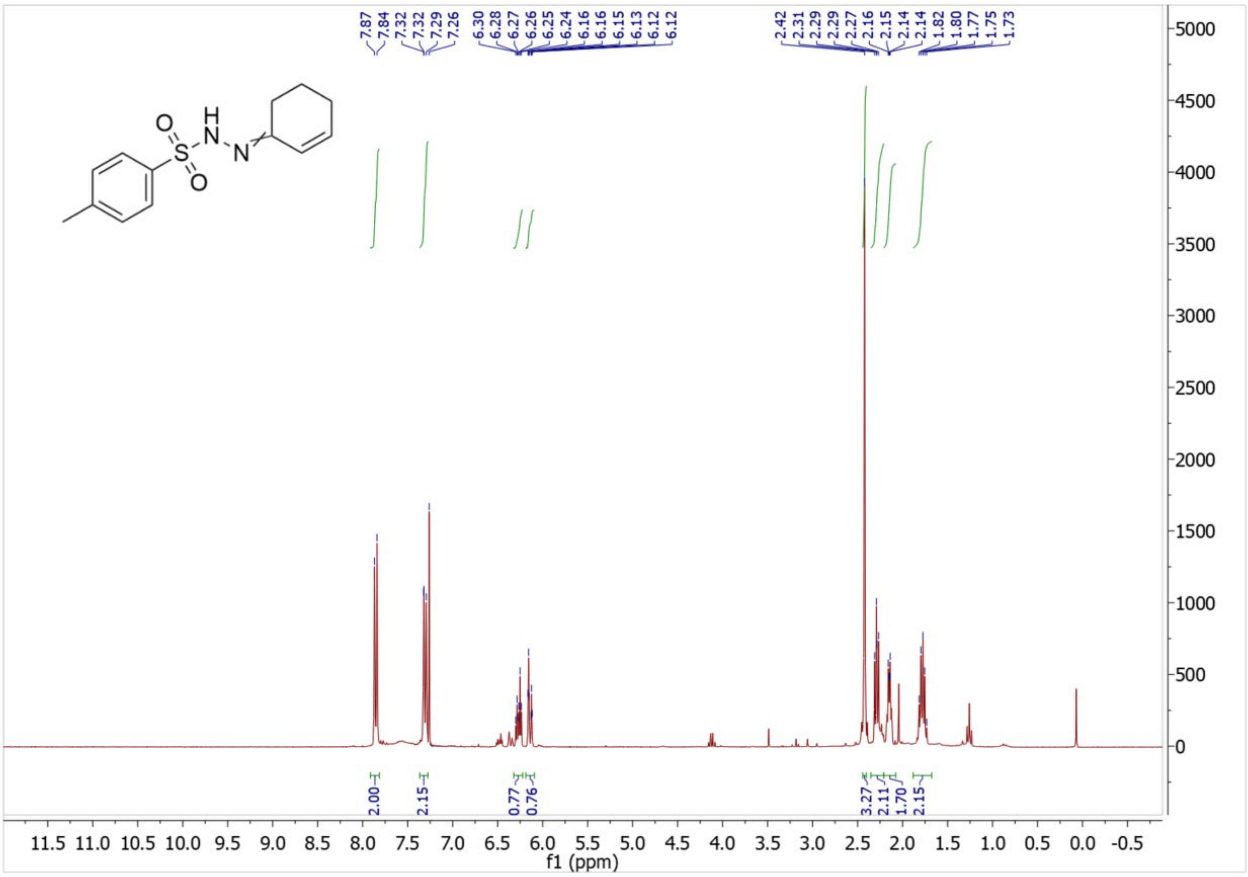

N'-(1-benzylpiperidin-4-ylidene)-4-methylbenzenesulfonohydrazide (**2t**)

In a round bottom flask 4-methylbenzenesulfonohydrazide (0.37 g; 2.00 mmol) and 1-benzylpiperidin-4-one (0.38 g; 2.00 mmol; 0.37 mL) were dissolved in 5 mL ethanol. The resulting mixture was stirred at room temperature for 5 hours. After completion of the reaction the product was collected by filtration.

Yield: 0.26 g (36%); pale brown solid; ^{1}H NMR (300 MHz, DMSO-d_6) δ 10.20 (s; 1H), 7.72 (d; J = 7.9 Hz; 2H), 7.39 (d; J = 8.0 Hz; 2H), 7.34 – 7.22 (m; 5H), 3.49 (s; 2H), 2.49 – 2.30 (m; 9H), 2.18 (t; J = 5.7 Hz; 2H) ppm.

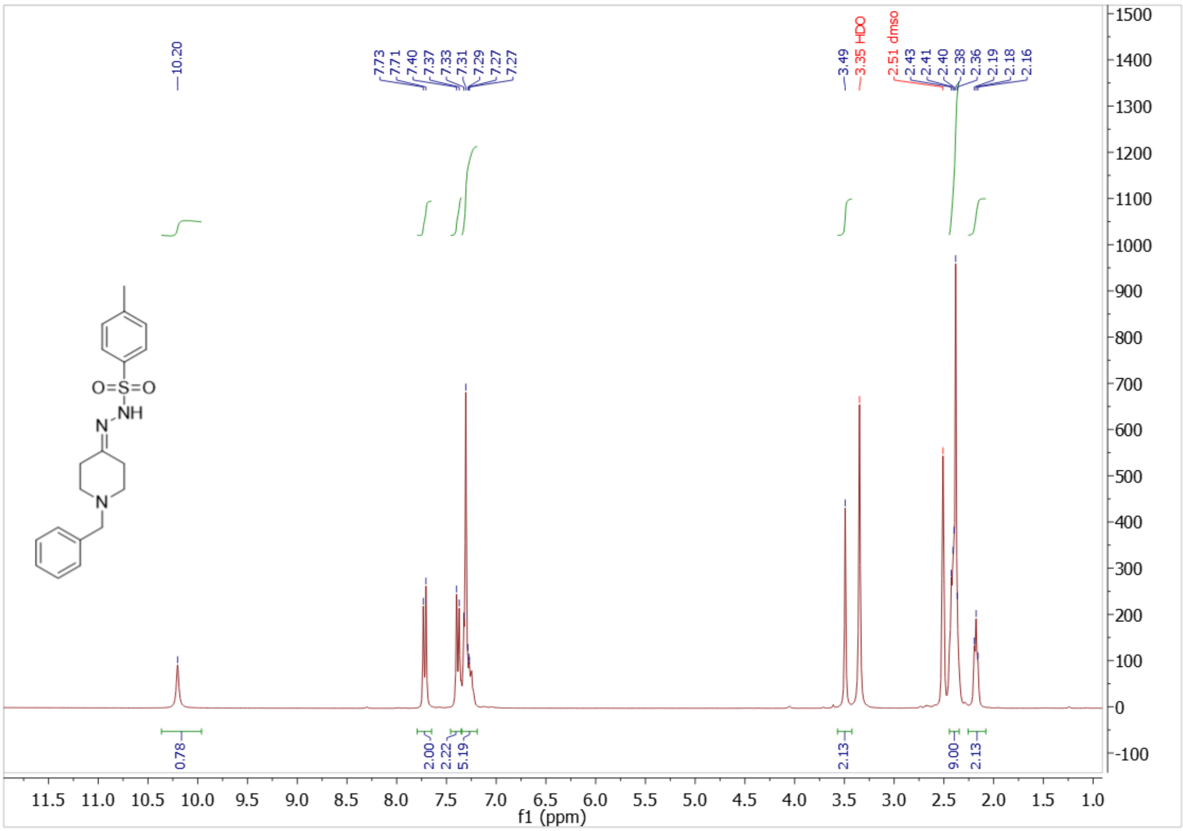

N′-benzylidene-4-methylbenzenesulfonohydrazide (**3a**)

In a round bottom flask 4-methylbenzenesulfonohydrazide (0.37 g; 2.00 mmol) and benzaldehyde (0.21 g; 2.00 mmol; 0.20 mL) were dissolved in 16 mL ethanol. The resulting mixture was stirred at room temperature for 6 hours. After completion of the reaction the solvent was evaporated.

Yield: 0.51 g (92%); white solid; ^{1}H NMR (300 MHz, DMSO-d_6) δ 11.45 (s, 1H), 7.93 (s, 1H), 7.79 (d, J = 7.9 Hz, 2H), 7.62 – 7.48 (m, 2H), 7.48 – 7.31 (m, 5H), 2.35 (s, 3H) ppm.

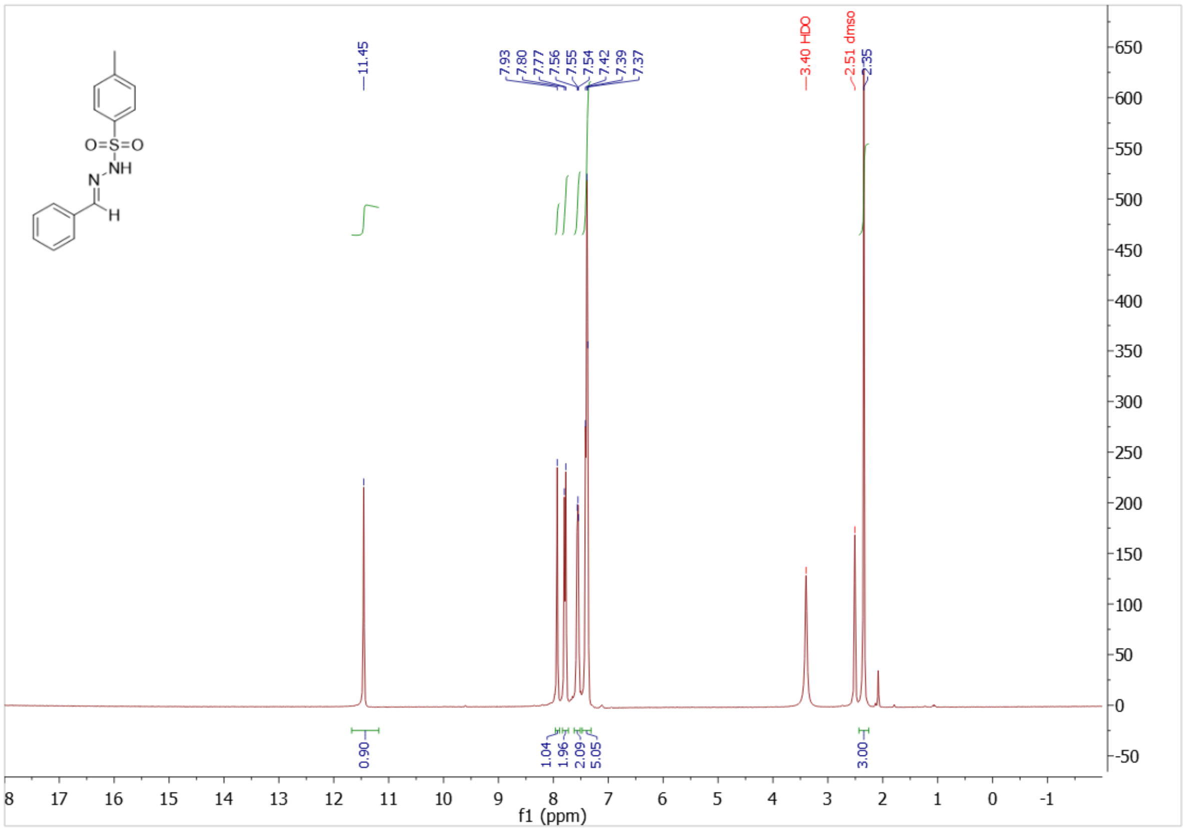

4-((2-Tosylhydrazinylidene)methyl)benzoic acid (**3b**)

In a round bottom flask 4-methylbenzenesulfonohydrazide (0.19 g; 1.00 mmol) and 4-formylbenzoic acid (0.15 g; 1.00 mmol) were dissolved in 15 mL ethanol. The resulting mixture was stirred at room temperature for 6 hours. After completion of the reaction the solvent was evaporated.

Yield: 0.32 g (100%); white solid; ^{1}H NMR (300 MHz, DMSO-d_6) δ 13.06 (s, 1H), 11.68 (s, 1H), 8.02 – 7.88 (m, 3H), 7.79 (d, J = 8.1 Hz, 2H), 7.68 (d, J = 8.1 Hz, 2H), 7.41 (d, J = 8.1 Hz, 2H), 2.35 (s, 3H).

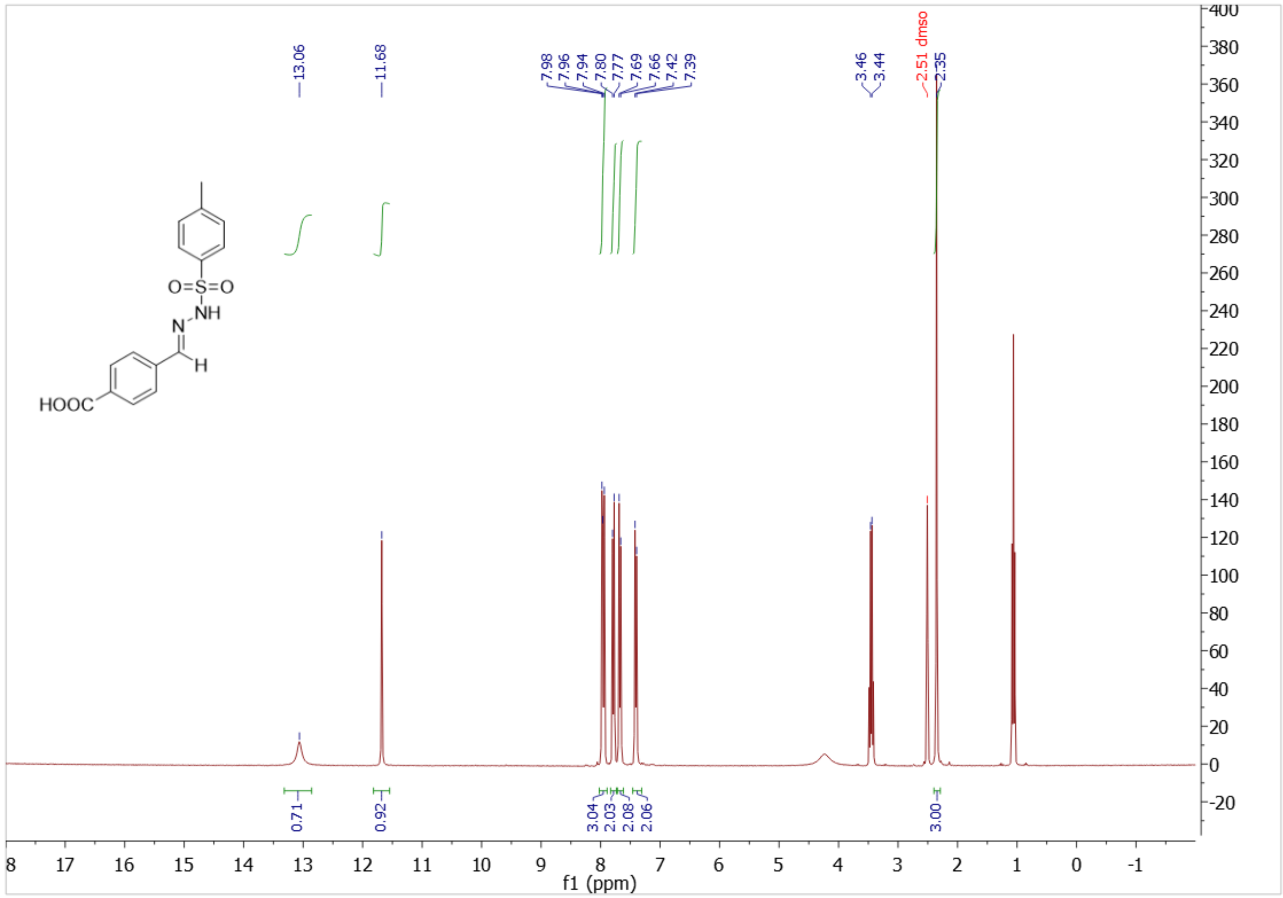

4-Methyl-*N′*-(2-nitrobenzylidene)benzenesulfonohydrazide (**3c**)

In a round bottom flask 4-methylbenzenesulfonohydrazide (0.19 g; 1.00 mmol) and 2-nitrobenzaldehyde (0.15 g; 1.00 mmol) were dissolved in 15 mL ethanol. The resulting mixture was stirred at 70 °C for 2 hours. After completion of the reaction the solvent was evaporated.

Yield: 0.32 g (100%); yellow solid; m.p.: 145 °C; ^{1}H NMR (300 MHz, DMSO-d_6) δ 11.91 (s; 1H), 8.30 (s; 1H), 8.02 (d; J = 8.1 Hz; 1H), 7.89 – 7.72 (m; 4H), 7.63 (t; J = 7.8 Hz; 1H), 7.42 (d; J = 7.9 Hz; 2H), 2.37 (s; 3H) ppm; ^{13}C NMR (75 MHz, DMSO-d_6) δ 148.30; 144.12; 142.77; 136.55; 134.19; 131.13; 130.24; 128.46; 128.28; 127.63; 125.09; 21.47 ppm; HRMS (ESI/Q-TOF) m/z: $[M+H]^+$ Calcd. for $C_{14}H_{14}N_3O_4S$ 320.0705; found 320.0708.

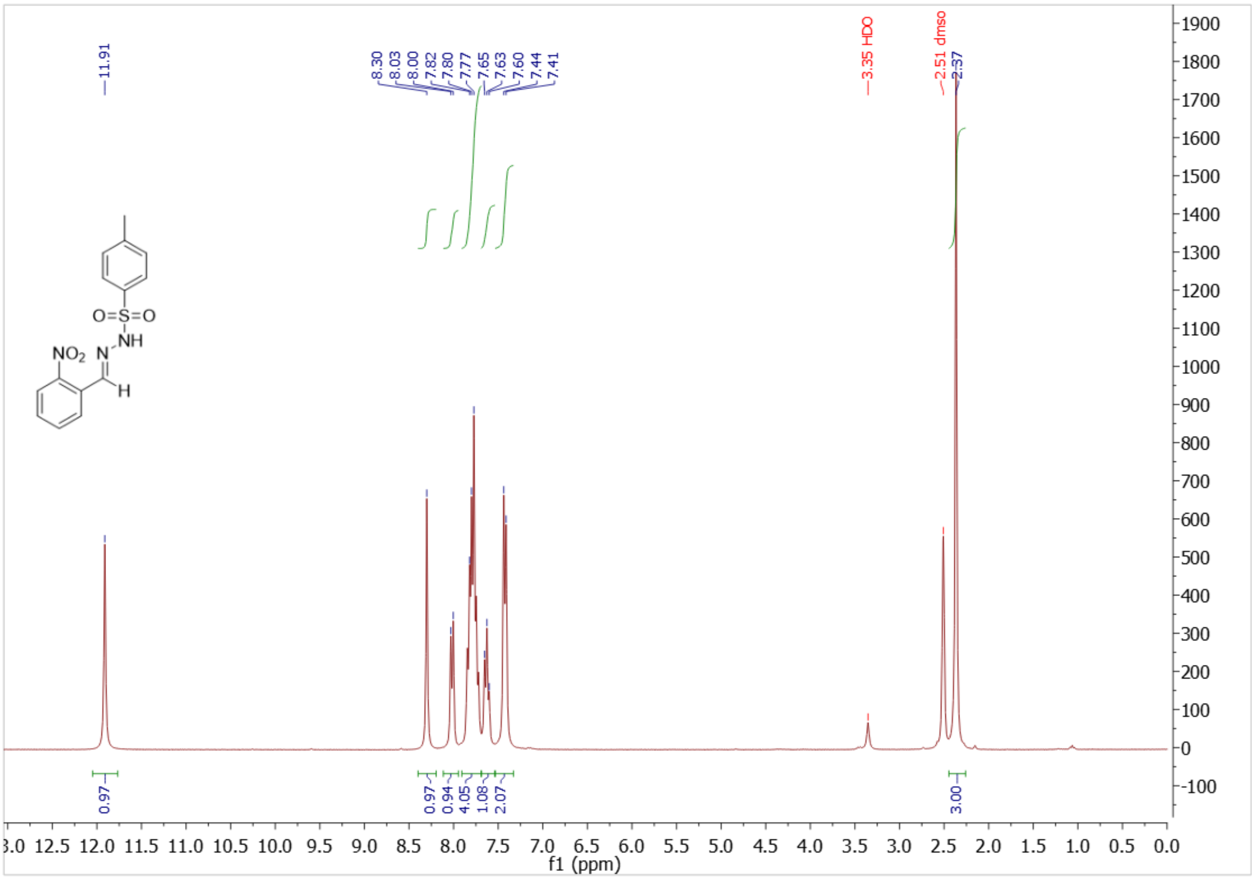

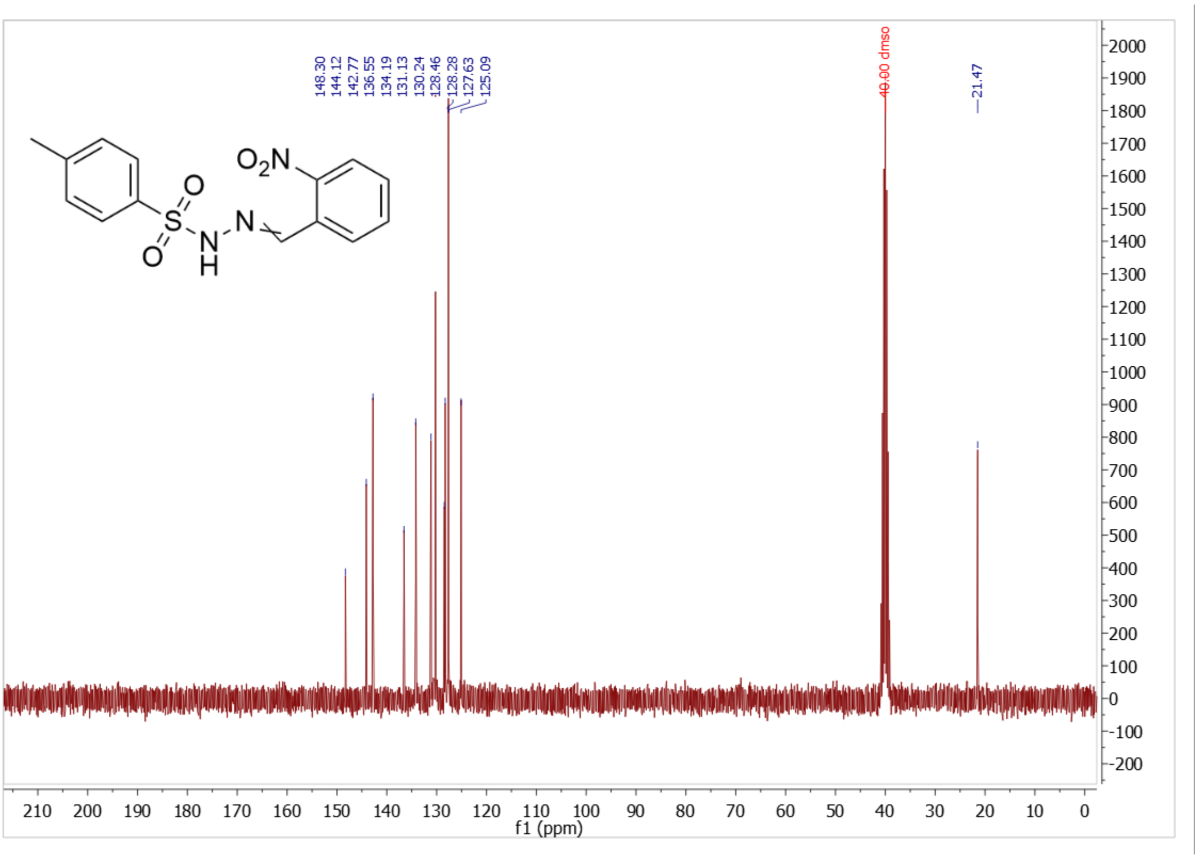

N-(4-((2-tosylhydrazinylidene)methyl)phenyl)acetamide (**3d**)

In a round bottom flask 4-methylbenzenesulfonohydrazide (0.19 g; 1.00 mmol) and 4-acetamidobenzaldehyde (0.16 g; 1.00 mmol) were dissolved in 15 mL ethanol. The resulting mixture was stirred at room temperature for 1 day. After completion of the reaction the solvent was evaporated.

Yield: 0.33 g (100%); white solid; m.p.: 210 °C; ^{1}H NMR (300 MHz, DMSO-d_6) δ 11.30 (s, 1H), 10.09 (s, 1H), 7.85 (s, 1H), 7.77 (d, J = 8.0 Hz, 2H), 7.62 (d, J = 8.3 Hz, 2H), 7.49 (d, J = 8.3 Hz, 2H), 7.40 (d, J = 7.9 Hz, 2H), 2.35 (s, 3H), 2.06 (s, 3H) ppm; ^{13}C NMR (75 MHz, DMSO-d_6) δ 168.99; 147.32; 143.81; 141.43; 136.68; 130.08; 128.73; 127.92; 127.69; 119.32; 24.53; 21.45 ppm; HRMS (ESI/Q-TOF) m/z: $[M+H]^+$ Calcd. for $C_{16}H_{18}N_3O_3S$ 332.1068; found 332.1067.

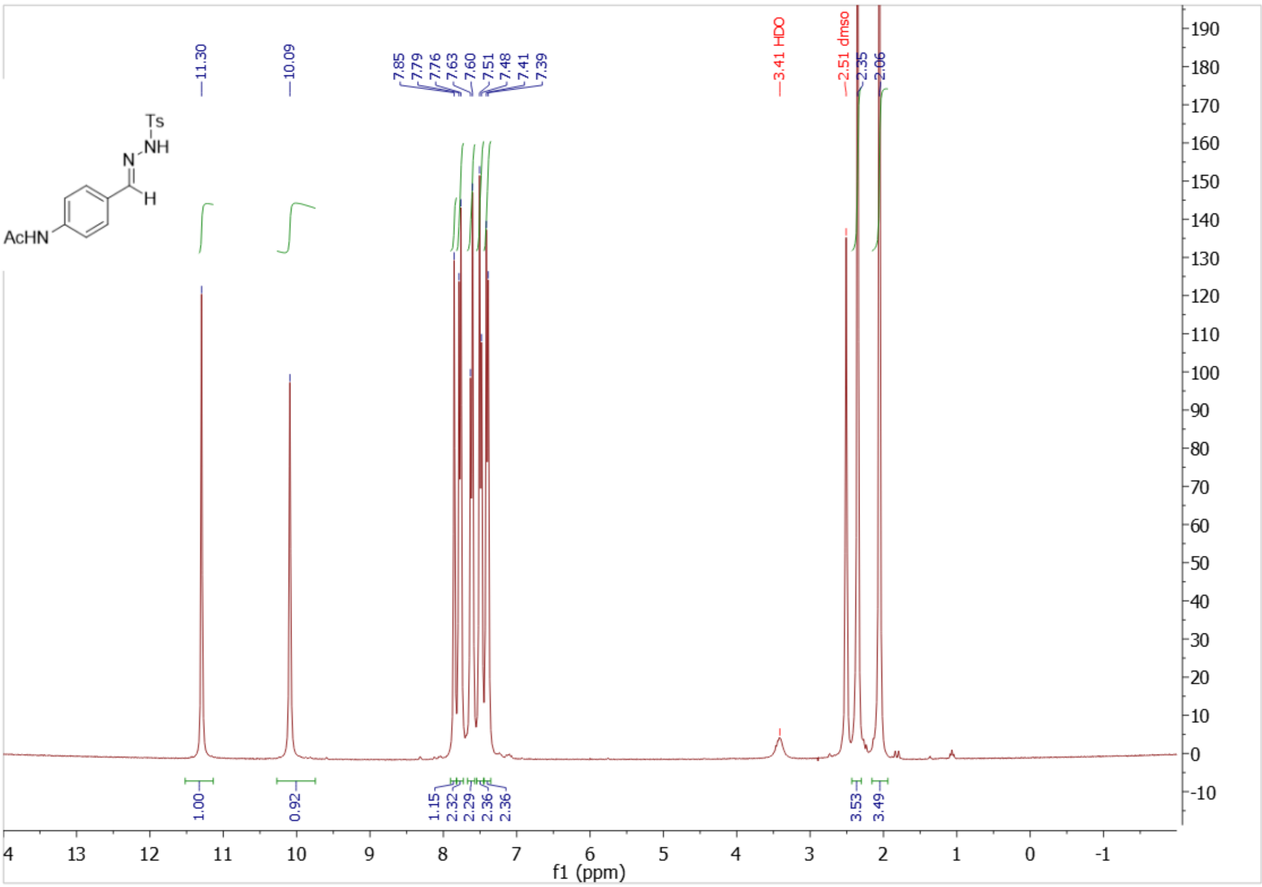

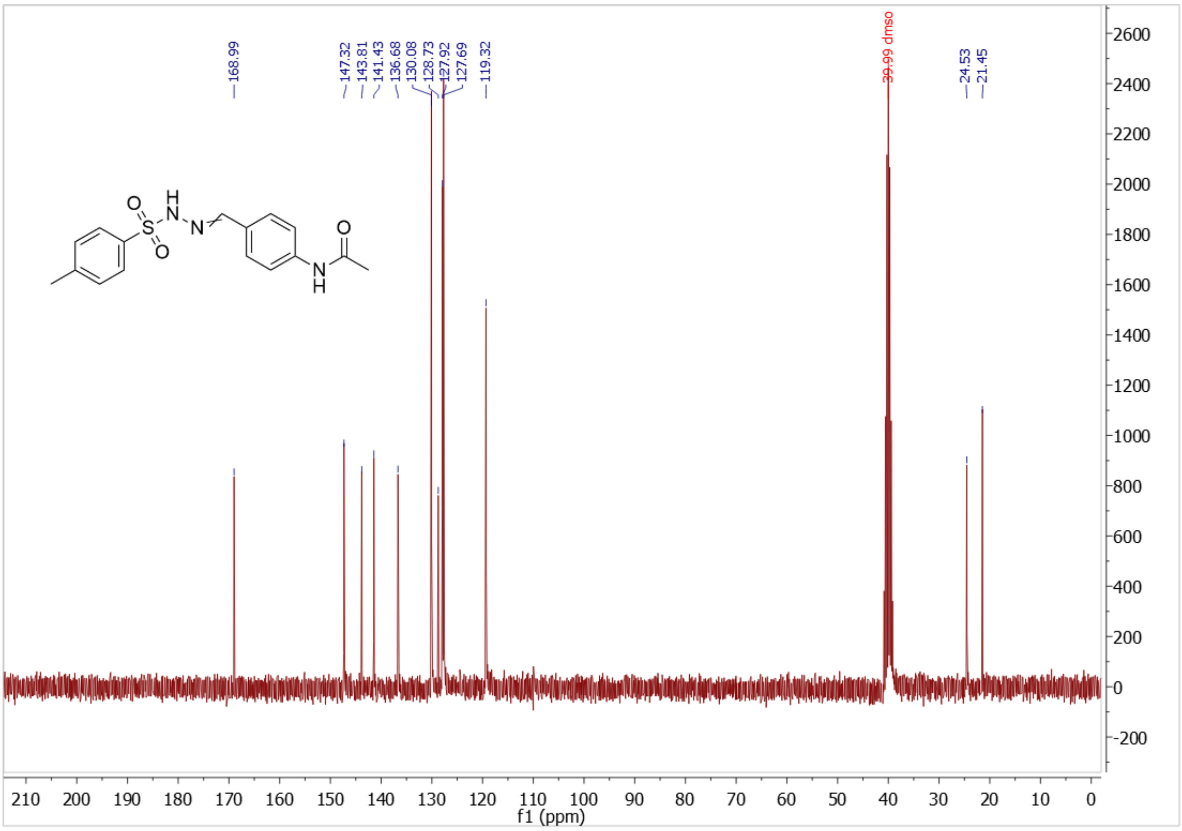

N'-(3,5-bis(trifluoromethyl)benzylidene)-4-methylbenzenesulfonohydrazide (**3e**)

In a round bottom flask 4-methylbenzenesulfonohydrazide (0.19 g; 1.00 mmol) and 3,5-bis(trifluoromethyl)benzaldehyde (0.24 g; 1.00 mmol; 0.17 mL) were dissolved in 15 mL ethanol. The resulting mixture was stirred at room temperature for 4 days. After completion of the reaction the solvent was evaporated.

Yield: 0.40 g (96%); white solid; m.p.: 120-122 °C; ^{1}H NMR (300 MHz, DMSO-d_6) δ 11.99 (s; 1H), 8.19 (s; 2H), 8.11 (s; 1H), 8.07 (s; 1H), 7.78 (d; J = 7.9 Hz; 2H), 7.40 (d; J = 8.0 Hz; 2H), 2.35 (s; 3H) ppm; ^{13}C NMR (75 MHz, DMSO-d_6) δ 144.18; 143.92; 136.94; 136.46; 131.46; 131.02; 130.17; 127.62; 127.62; 127.26; 125.32; 123.33; 121.71; 21.40 ppm; HRMS (ESI/Q-TOF) m/z: [M+H]$^+$ Calcd. for $C_{16}H_{13}F_6N_2O_2S$ 411.0601; found 411.0608.

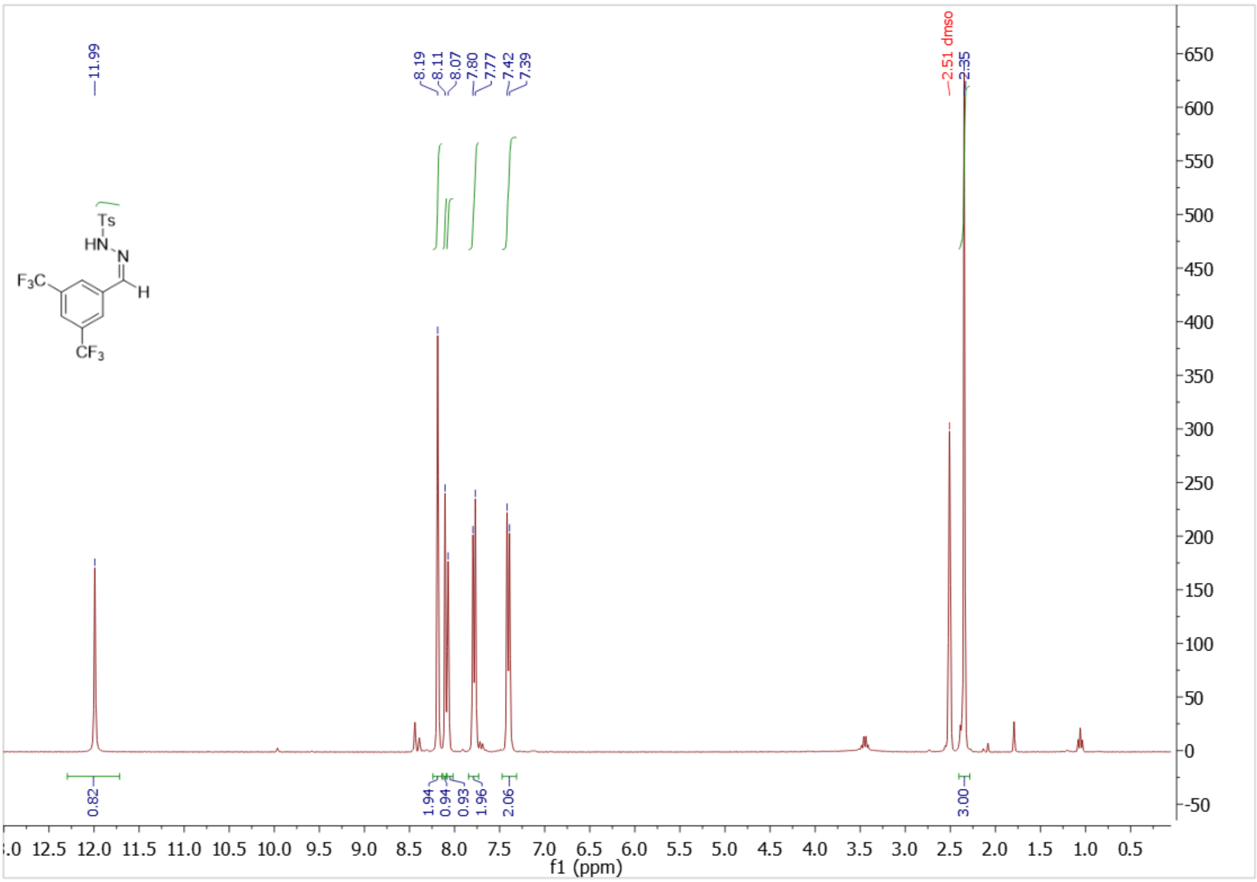

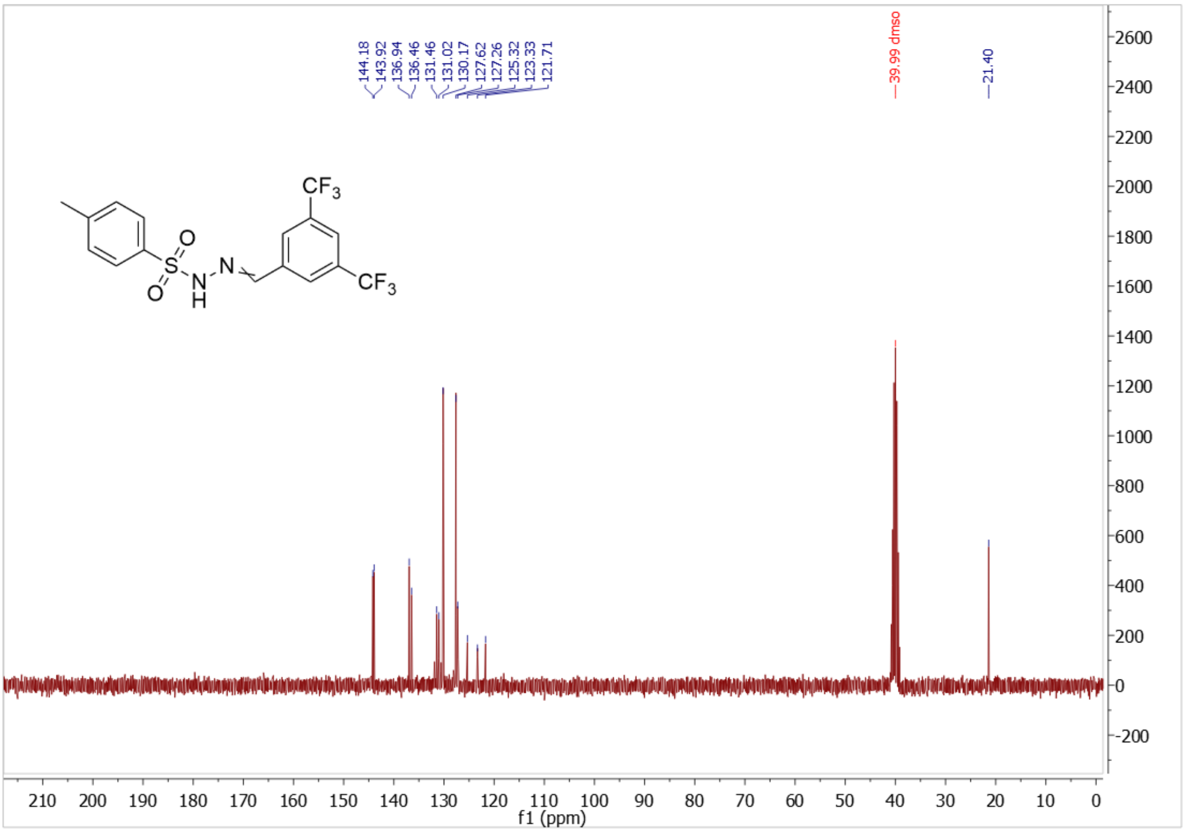

4-Methyl-*N′*-(pyridin-3-ylmethylene)benzenesulfonohydrazide (**3f**)

In a round bottom flask 4-methylbenzenesulfonohydrazide (0.19 g; 1.00 mmol) and 3-pyiridinecarboxaldehyde (0.11 g; 1.00 mmol) were dissolved in 15 mL ethanol. The resulting mixture was stirred at room temperature for 3 hours. After completion of the reaction the product was collected by filtration.

Yield: 0.14 g (50%); white solid; ^{1}H NMR (300 MHz, DMSO-d_6) δ 11.66 (s; 1H), 8.71 (s; 1H), 8.57 (dd; J = 4.8; 1.7 Hz; 1H), 8.03 – 7.87 (m; 2H), 7.78 (d; J = 7.9 Hz; 2H), 7.42 (d; J = 7.7 Hz; 3H), 2.37 (s; 3H) ppm.

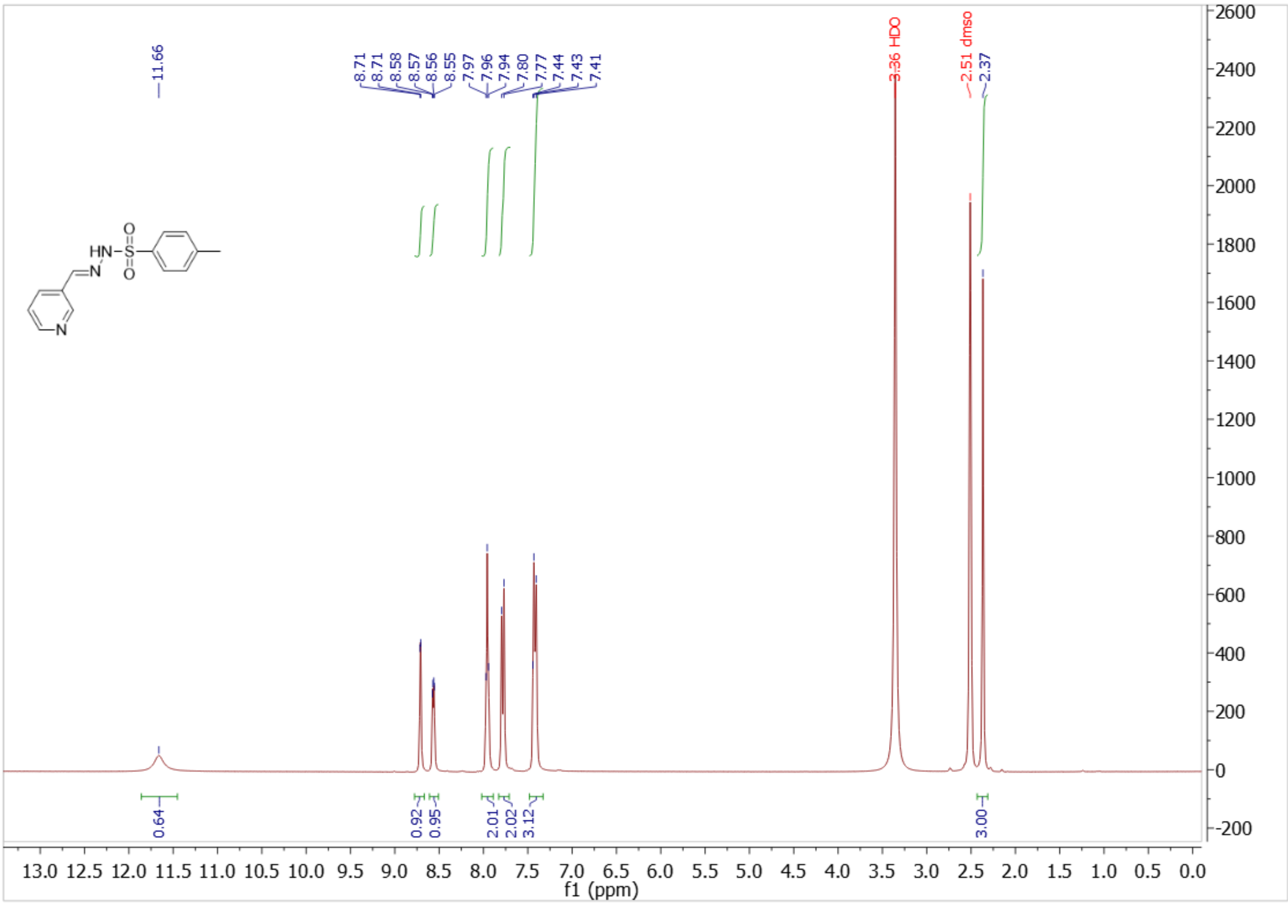

N'-((1H-imidazol-4-yl)methylene)-4-methylbenzenesulfonohydrazide (**3g**)

In a round bottom flask 4-methylbenzenesulfonohydrazide (0.19 g; 1.00 mmol) and 1H-imidazole-4-carbaldehyde (0.10 g; 1.00 mmol) were dissolved in 15 mL ethanol. The resulting mixture was stirred at room temperature for 3 hours. After completion of the reaction the solvent was evaporated.

Yield: 0.25 g (95%); pale brown solid; m.p.: 151 °C; ^{1}H NMR (300 MHz, DMSO-d_6) δ 13.01 (s; 1H), 8.03 (s; 1H), 7.71 (d; J = 8.0 Hz; 2H), 7.64 (s; 1H), 7.44 – 7.24 (m; 3H), 2.35 (s; 3H) ppm; ^{13}C NMR (75 MHz, DMSO-d_6) δ 143.89; 136.70; 136.59; 136.01; 135.63; 130.19; 127.46;

121.68; 21.44 ppm; HRMS (ESI/Q-TOF) m/z: $[M+H]^+$ Calcd. for $C_{11}H_{13}N_4O_2S$ 265.0759; found 265.0755.

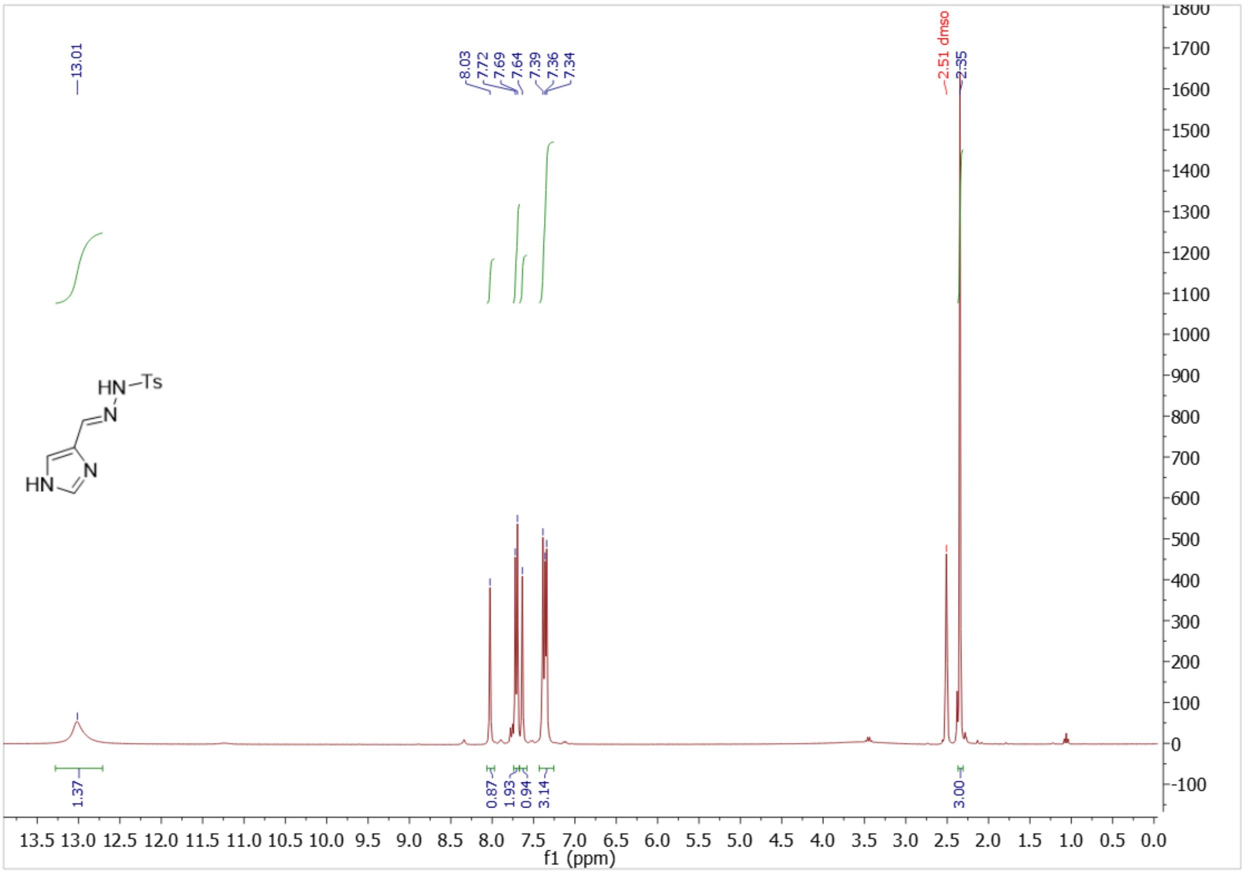

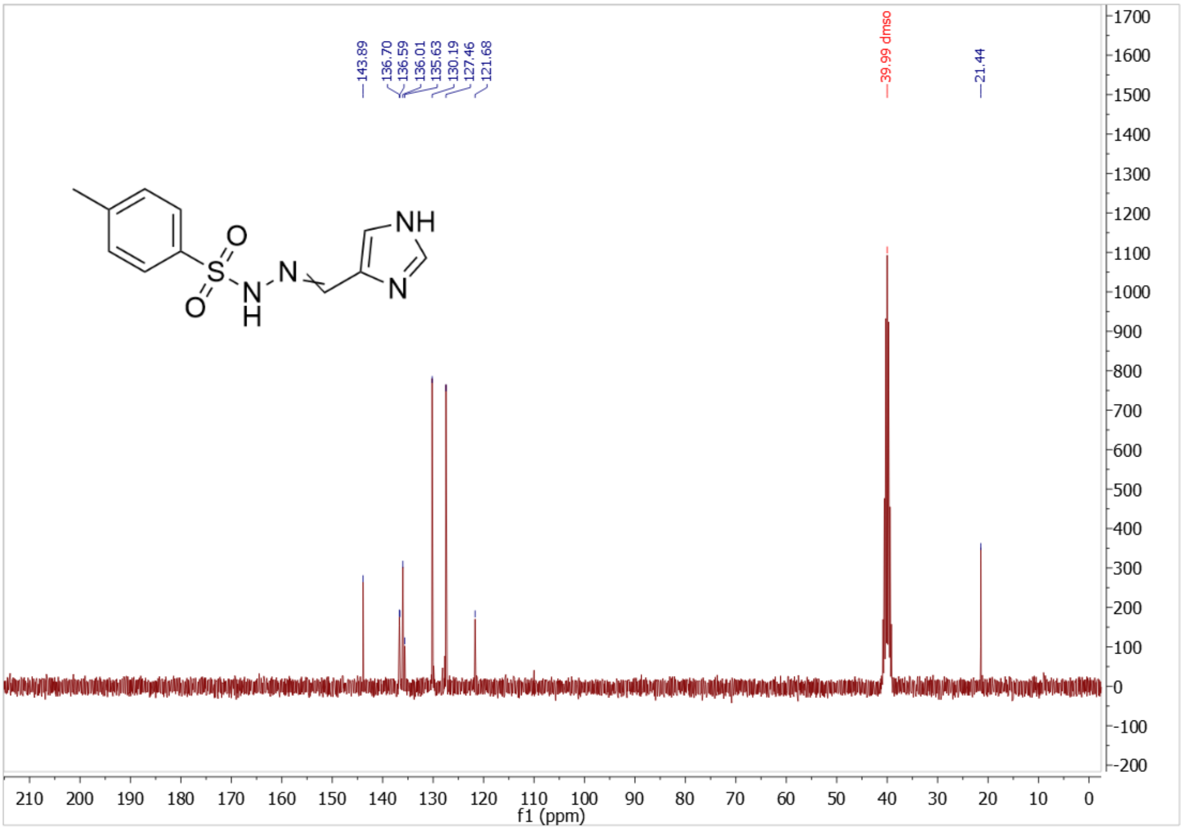

N'-((2-bromothiophen-3-yl)methylene)-4-methylbenzenesulfonohydrazide (**3h**)

In a round bottom flask 4-methylbenzenesulfonohydrazide (0.19 g; 1.00 mmol) and 5-bromothiophene-2-carbaldehyde (0.19 g; 1.00 mmol; 0.12 mL) were dissolved in 15 mL ethanol. The resulting mixture was stirred at room temperature for 1 day. After completion of the reaction the solvent was evaporated.

Yield: 0.34 g (95%); pale brown solid; m.p.: 99-100 °C; ^{1}H NMR (300 MHz, DMSO-d_6) δ 11.47 (s; 1H), 8.00 (s; 1H), 7.72 (d; J = 8.0 Hz; 2H), 7.42 (d; J = 8.0 Hz; 2H), 7.20 (s; 2H), 2.37 (s; 3H) ppm; ^{13}C NMR (75 MHz, DMSO-d_6) δ 144.02 , 141.72 , 140.61 , 136.32 , 131.67 , 131.63 , 130.11 , 127.64 , 115.00 , 21.45 ppm; HRMS (ESI/Q-TOF) m/z: $[M+H]^+$ Calcd. for $C_{12}H_{12}BrN_2O_2S_2$ 358.9523; found 358.9528, 360.9504.

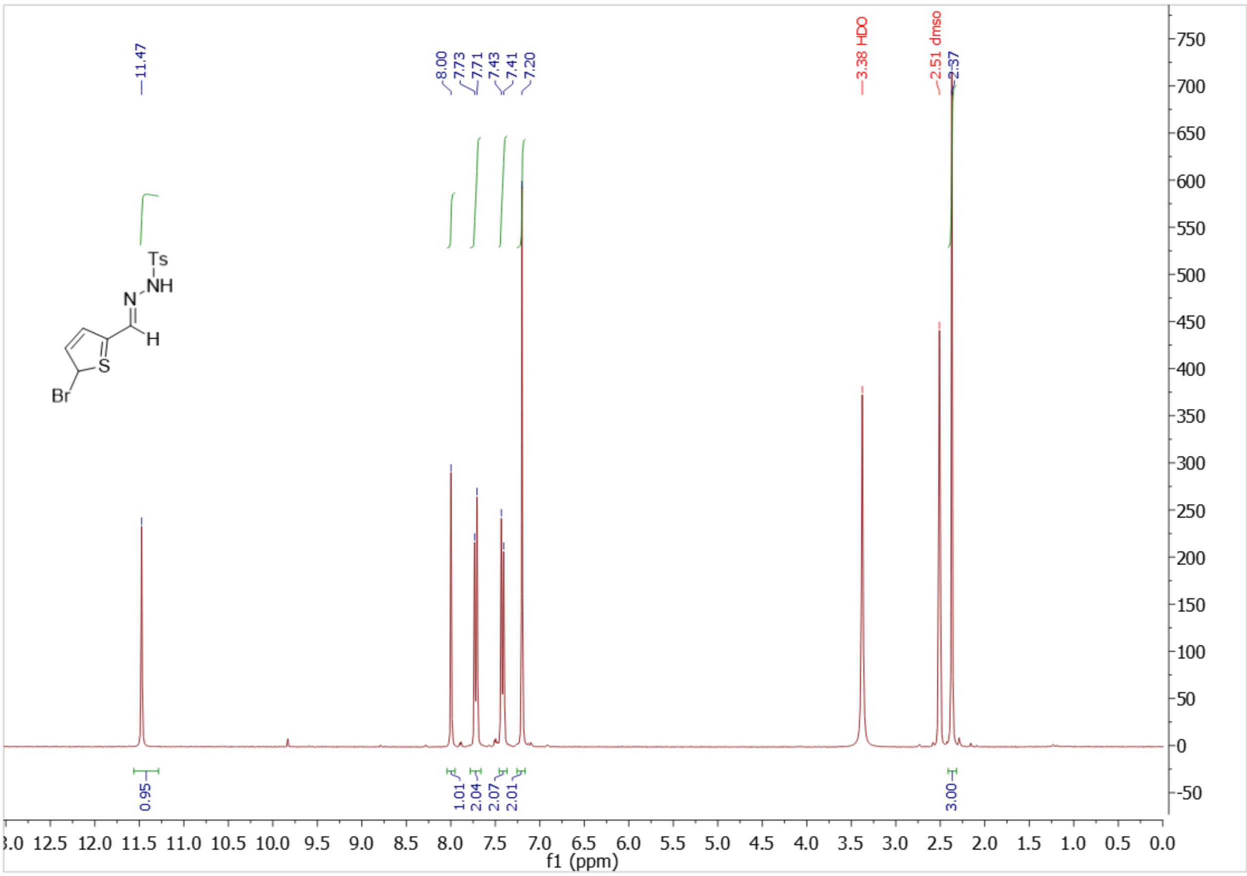

11.47
8.00
7.73
7.71
7.43
7.41
7.20
3.38 HDO
2.51 dmso
2.37
Ts
NH
N
H
S
Br
0.95
1.01
2.04
2.07
2.01
3.00
f1 (ppm)

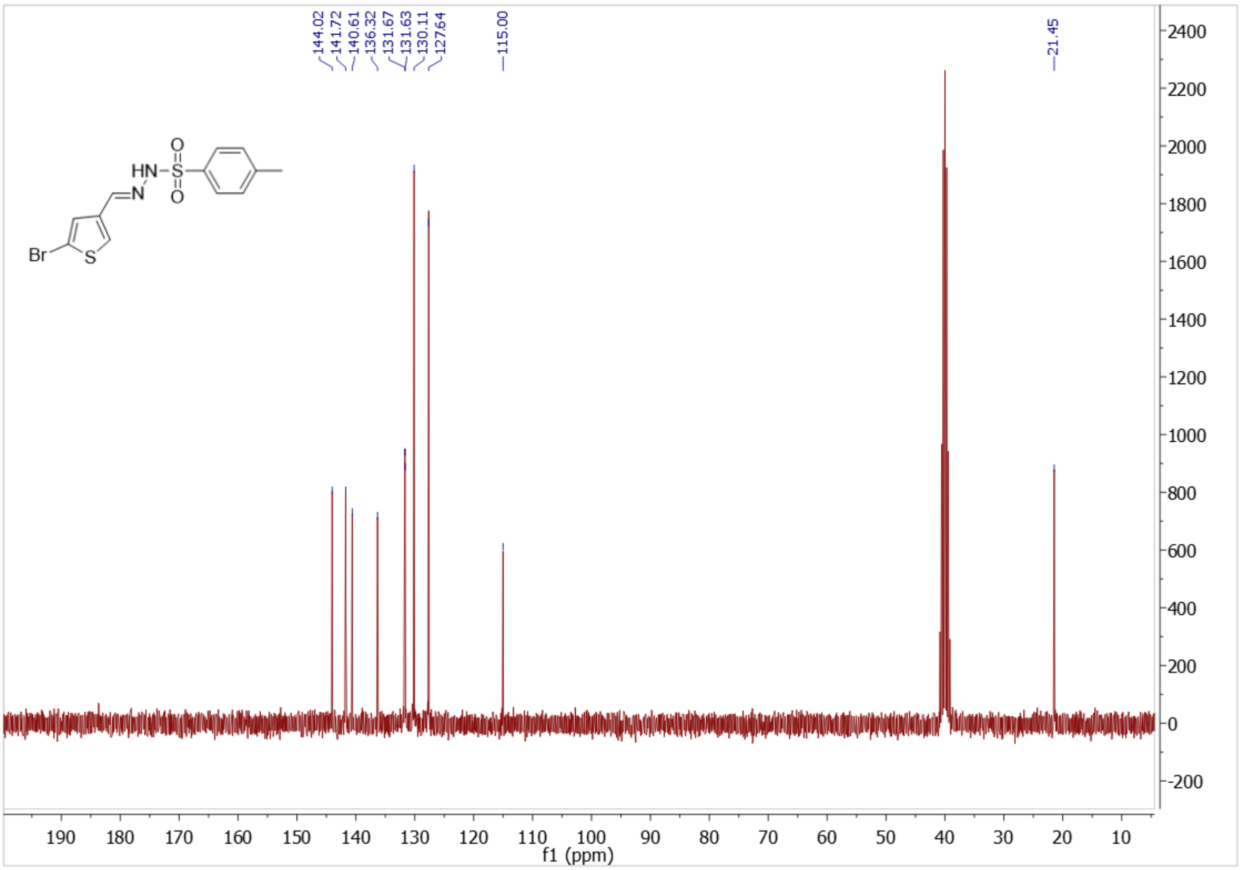

144.02
141.72
140.61
136.32
131.67
131.63
130.11
127.64
115.00
21.45
O
HN
S
N
O
Br
S
f1 (ppm)

N'-((1H-indol-3-yl)methylene)-4-methylbenzenesulfonohydrazide (**3i**)

In a round bottom flask 4-methylbenzenesulfonohydrazide (0.19 g; 1.00 mmol) and 1H-indole-3-carbaldehyde (0.15 g; 1.00 mmol) were dissolved in 15 mL ethanol. The resulting mixture was stirred at room temperature for 2 days. After completion of the reaction the solvent was evaporated.

Yield: 0.31 g (100%); brown solid; ^{1}H NMR (300 MHz, DMSO-d_6) δ 11.51 (s; 1H), 10.87 (s; 1H), 8.11 (s; 1H), 8.00 (d; J = 7.5 Hz; 1H), 7.83 (d; J = 8.0 Hz; 2H), 7.72 (d; J = 2.7 Hz; 1H), 7.51 – 7.31 (m; 3H), 7.27 – 7.03 (m; 2H), 2.33 (s; 3H) ppm.

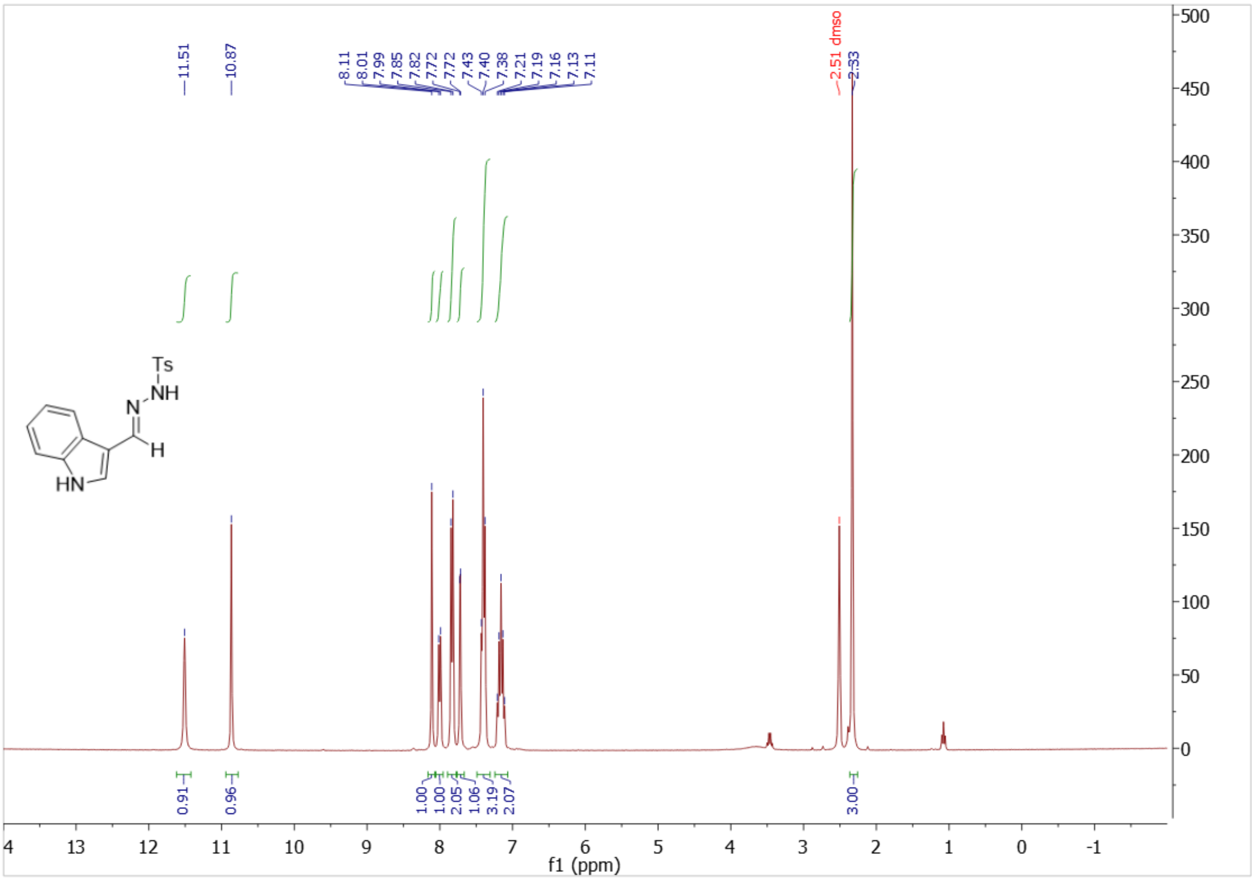

4-Methyl-*N'*-(2-phenylethylidene)benzenesulfonohydrazide (**3j**)

In a round bottom flask 4-methylbenzenesulfonohydrazide (0.19 g; 1.00 mmol) and 2-phenylacetaldehyde (0.12 g; 1.00 mmol; 0.12 mL) were dissolved in 15 mL ethanol. The resulting mixture was stirred at room temperature for 1 day. After completion of the reaction the solvent was evaporated, and the crude was purified by normal-phase flash chromatography.

Yield: 0.11 g (38%); white solid; ^{1}H NMR (300 MHz, DMSO-d_6) δ 10.98 (s; 1H), 7.71 (d; J = 8.2 Hz; 2H), 7.42 (d; J = 8.0 Hz; 2H), 7.31 (t; J = 5.8 Hz; 1H), 7.27 – 7.15 (m; 3H), 7.05 – 6.96 (m; 2H), 3.42 (d; J = 5.8 Hz; 2H), 2.41 (s; 3H) ppm.

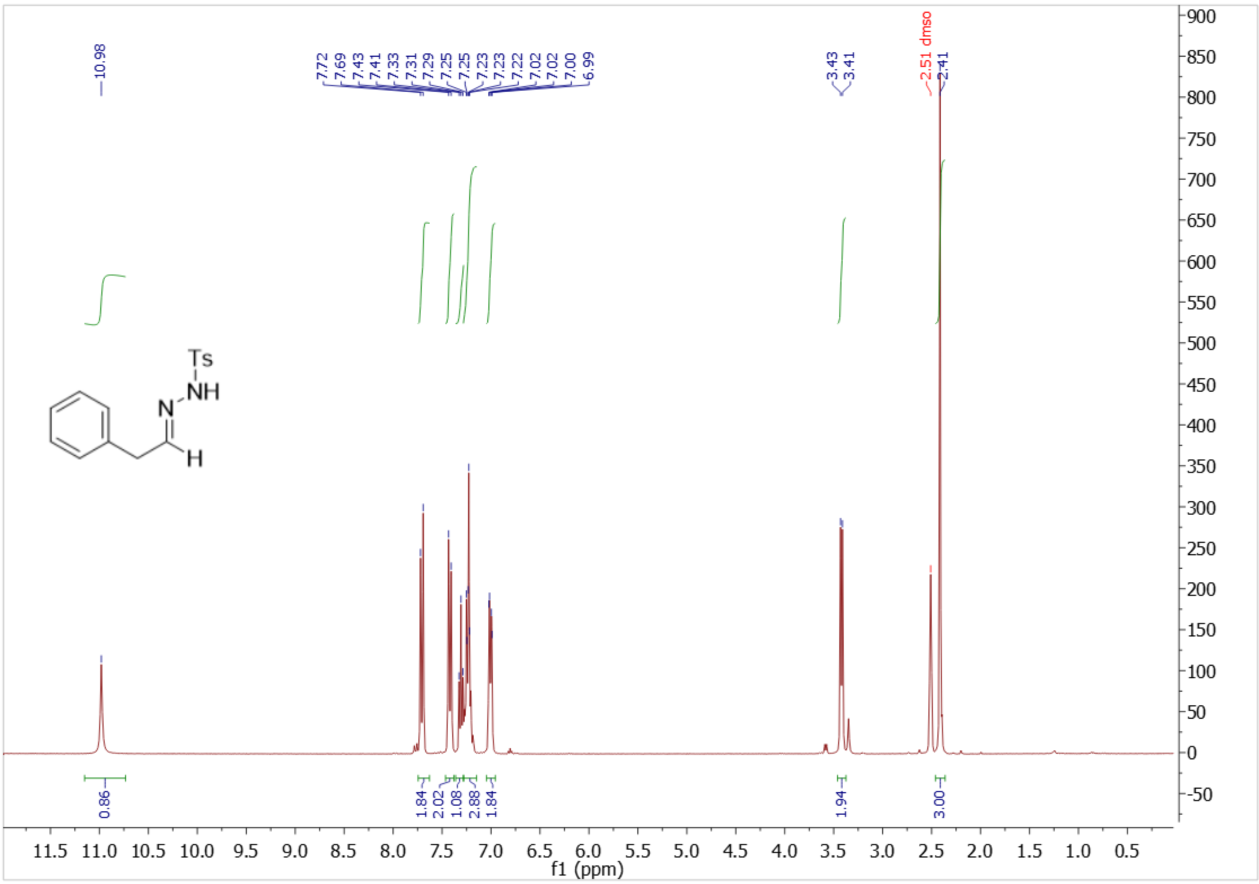

N'-((1-benzylpiperidin-4-yl)methylene)-4-methylbenzenesulfonohydrazide (**3k**)

In a round bottom flask 4-methylbenzenesulfonohydrazide (0.09 g; 0.50 mmol) and N-benzylpiperidine-4-carbaldehyde (0.10 g; 0.50 mmol) were dissolved in a mixture of 15 mL ethanol and 10 mL methanol. The resulting mixture was stirred at room temperature for 4 days. After completion of the reaction the solvent was evaporated and the crude was purified by normal-phase flash chromatography.

Yield: 0.04 g (19%); yellow gum; m.p.: 45 °C; ^{1}H NMR (300 MHz, Chloroform-*d*) δ 7.81 (m, *J* = 8.3 Hz, 2H), 7.56 – 7.20 (m, 7H), 7.09 (d, *J* = 5.2 Hz, 1H), 3.48 (s, 2H), 3.00 – 2.73 (m, 2H), 2.67 (s, 1H), 2.44 (s, 3H), 2.30 – 2.12 (m, 1H), 2.01 (m, *J* = 11.5, 11.1, 2.6 Hz, 2H), 1.68 (dd, *J* = 13.5, 3.8 Hz, 2H), 1.58 – 1.39 (m, 2H) ppm; ^{13}C NMR (75 MHz, Chloroform-*d*) δ 154.98 , 143.97 , 129.57 , 129.21 , 128.21 , 127.89 , 127.08 , 63.23 , 52.72 , 40.72 , 38.69 , 28.95 , 21.62 ppm; HRMS (ESI/Q-TOF) m/z: [M+H]$^{+}$ Calcd. for $C_{21}H_{27}N_{2}O_{2}S$ 372.1745; found 372.1750.

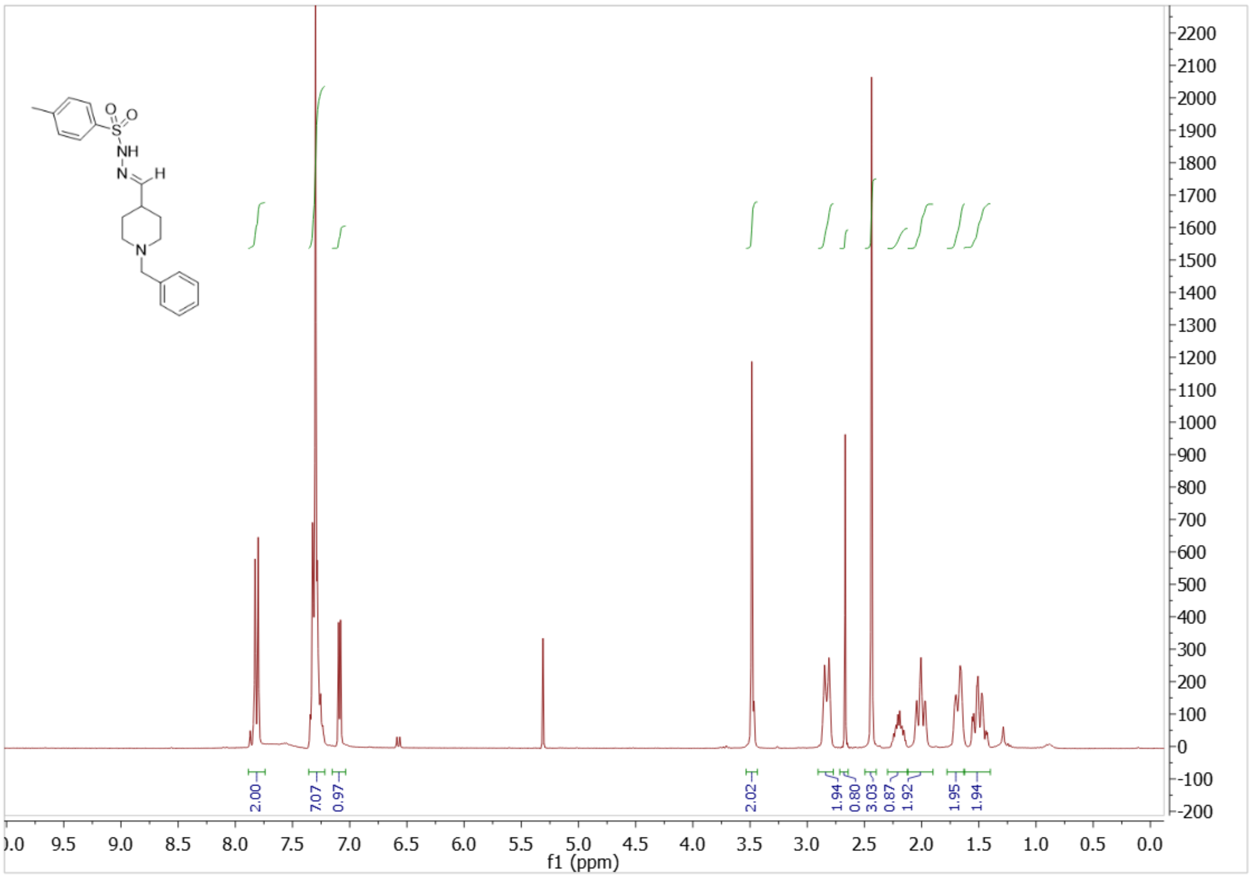

2.00
7.07
0.97
2.02
1.94
0.80
3.03
0.87
1.92
1.95
1.94
f1 (ppm)

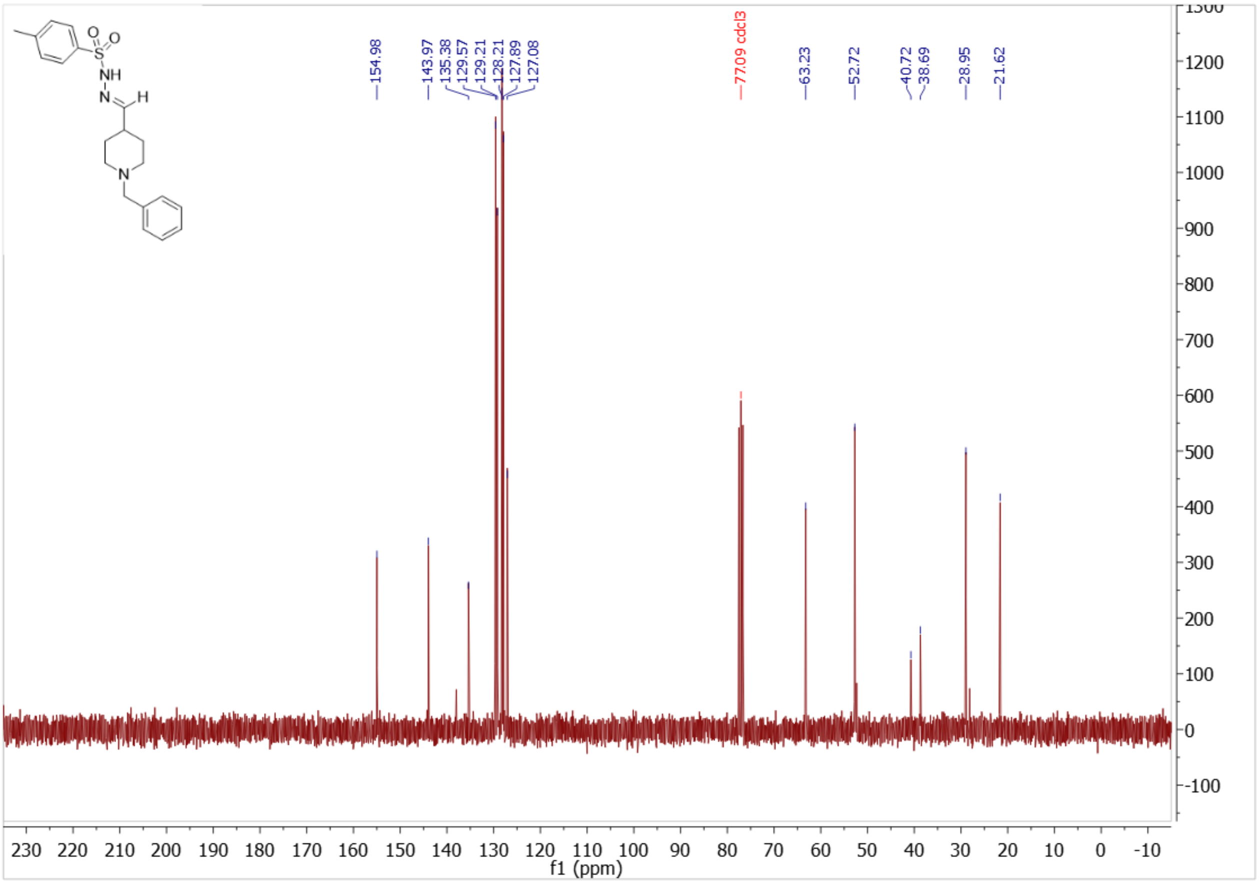

154.98
143.97
135.38
129.57
129.21
128.21
127.89
127.08
77.09 cdcl3
63.23
52.72
40.72
38.69
28.95
21.62
f1 (ppm)

4-Methyl-*N'*-((E)-3-phenylallylidene)benzenesulfonohydrazide (**3m**)

In a round bottom flask 4-methylbenzenesulfonohydrazide (0.28 g; 1.50 mmol) was dissolved in 10 mL methanol. To the mixture cinnamaldehyde (0.20 g; 1.50 mmol) was added dropwise. The resulting mixture was stirred at room temperature for 2 hours. After completion of the reaction After completion of the reaction the product was collected by filtration.

Yield: 0.45 g (100%); white solid; ^{1}H NMR (300 MHz, DMSO-d_6) δ 11.36 (s, 1H), 7.70 (d, J = 8.3 Hz, 3H), 7.57 – 7.49 (m, 2H), 7.44 – 7.27 (m, 5H), 6.96 – 6.72 (m, 2H), 2.35 (s, 3H) ppm.

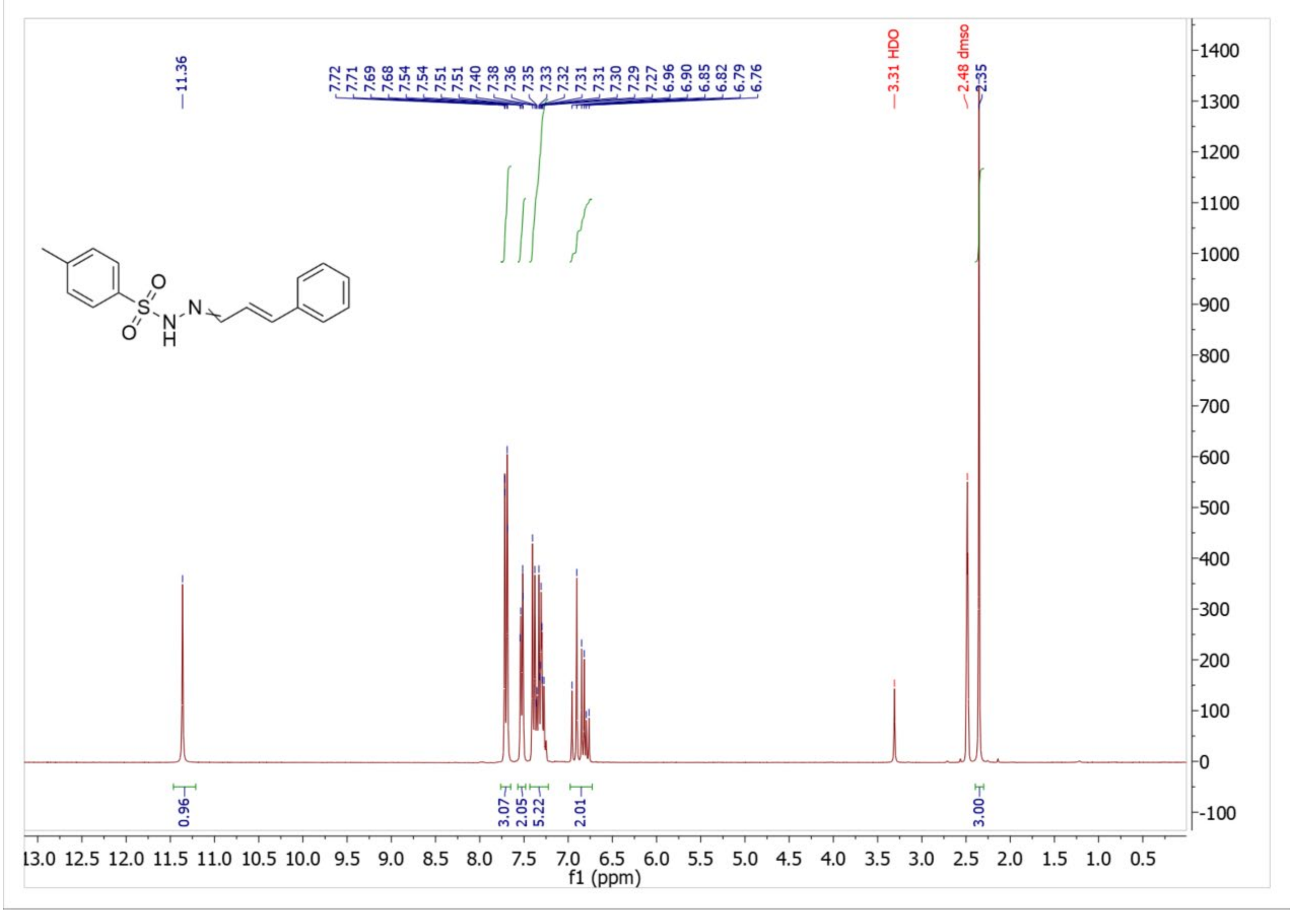

N'-(but-3-yn-2-ylidene)-4-methylbenzenesulfonohydrazide (**4a**)

In a round bottom flask 4-methylbenzenesulfonohydrazide (0.19 g; 1.00 mmol) and but-3-yn-2-one (0.07 g; 1.00 mmol; 0.08 mL) were dissolved in 5 mL ethanol. The resulting mixture was stirred at room temperature for 2 days. After completion of the reaction the solvent was evaporated and the crude was purified by normal-phase flash chromatography.

Yield: 0.09 g (39%); white solid; ^{1}H NMR (300 MHz, DMSO-d_6) δ 8.32 (d; J = 2.7 Hz; 1H), 7.83 (d; J = 8.4 Hz; 2H), 7.46 (d; 2H), 6.42 (d, J = 2.7 Hz; 1H), 2.39 (s; 3H), 2.17 (s; 3H) ppm.

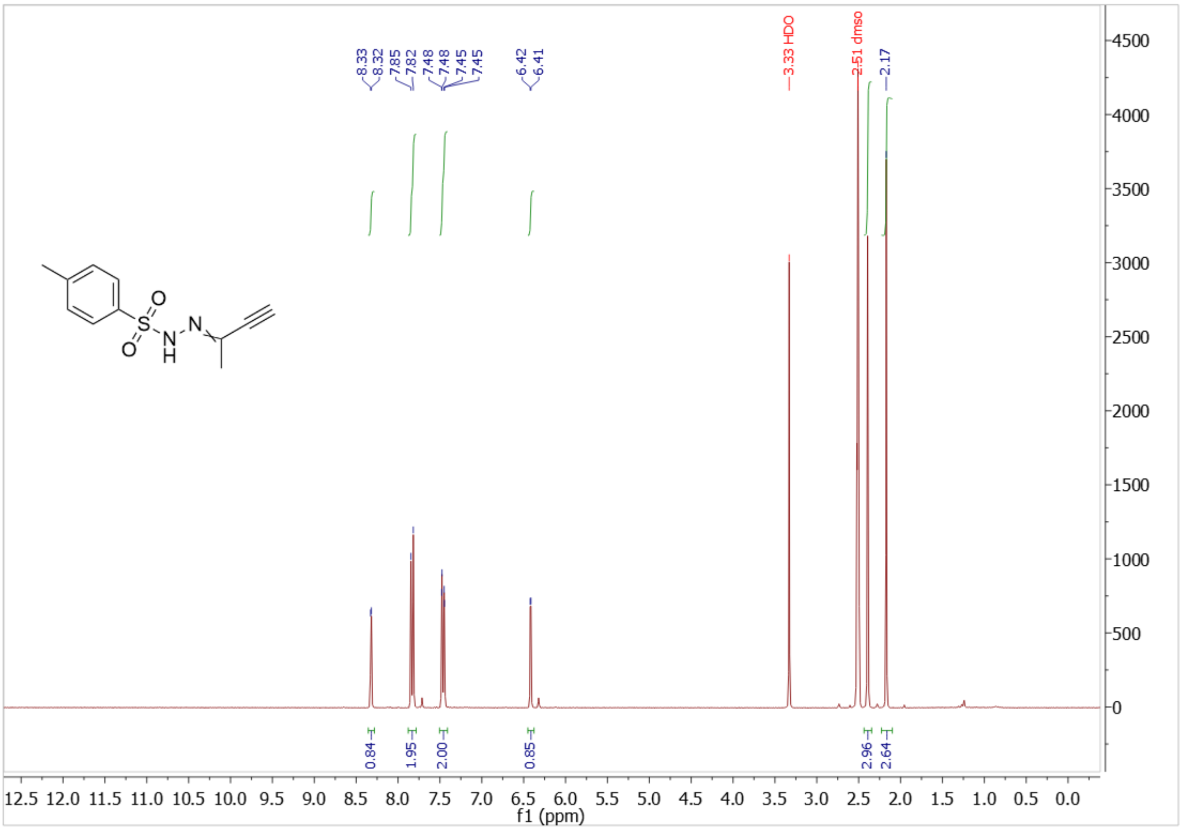

4-Methyl-*N'*-(1-(4-(prop-2-yn-1-ylamino)phenyl)ethylidene)benzenesulfonohydrazide (**4b**):

1-(4-(Prop-2-yn-1-ylamino)phenyl)ethan-1-one

In a round bottom flask 4-aminoacetophenone (0.81 g; 6,00 mmol), propargyl bromide (0.18 g; 1.5 mmol; 0.13 mL) and sodium carbonate (0.42 g; 1.5 mmol) were dissolved in 10 mL acetonitrile. The resulting mixture was stirred at room temperature for 1 days. The following day additional amount of propargyl bromide (0.18 g; 1.5 mmol; 0.13 mL) and sodium carbonate (0.42 g; 1.5 mmol) were dissolved in the mixture. The mixture was stirred at room temperature for 1 day. After that additional amount of propargyl bromide (0.18 g; 1.5 mmol; 0.13 mL) caesium(II)-carbonate (0.49 g; 1.5 mmol), potassium-iodide (0.25 g; 1.5 mmol) and 10 mL DMF were added to the mixture. The resulting mixture was stirred at 110 °C for 2 days. After completion of the reaction the solvent was evaporated and the crude was purified by normal-phase flash chromatography.

Yield: 0.05 g (5%); white solid; ^{1}H NMR (300 MHz, $CDCl_3$) δ 7.89 (d; *J* = 8.4 Hz; 2H), 6.68 (d; *J* = 8.5 Hz; 2H), 4.45 (t; *J* = 6.0 Hz; 1H), 4.03 (dd; *J* = 6.2, 2.5 Hz; 2H), 2.54 (s; 3H), 2.28 (t, *J* = 2.5 Hz; 1H) ppm.

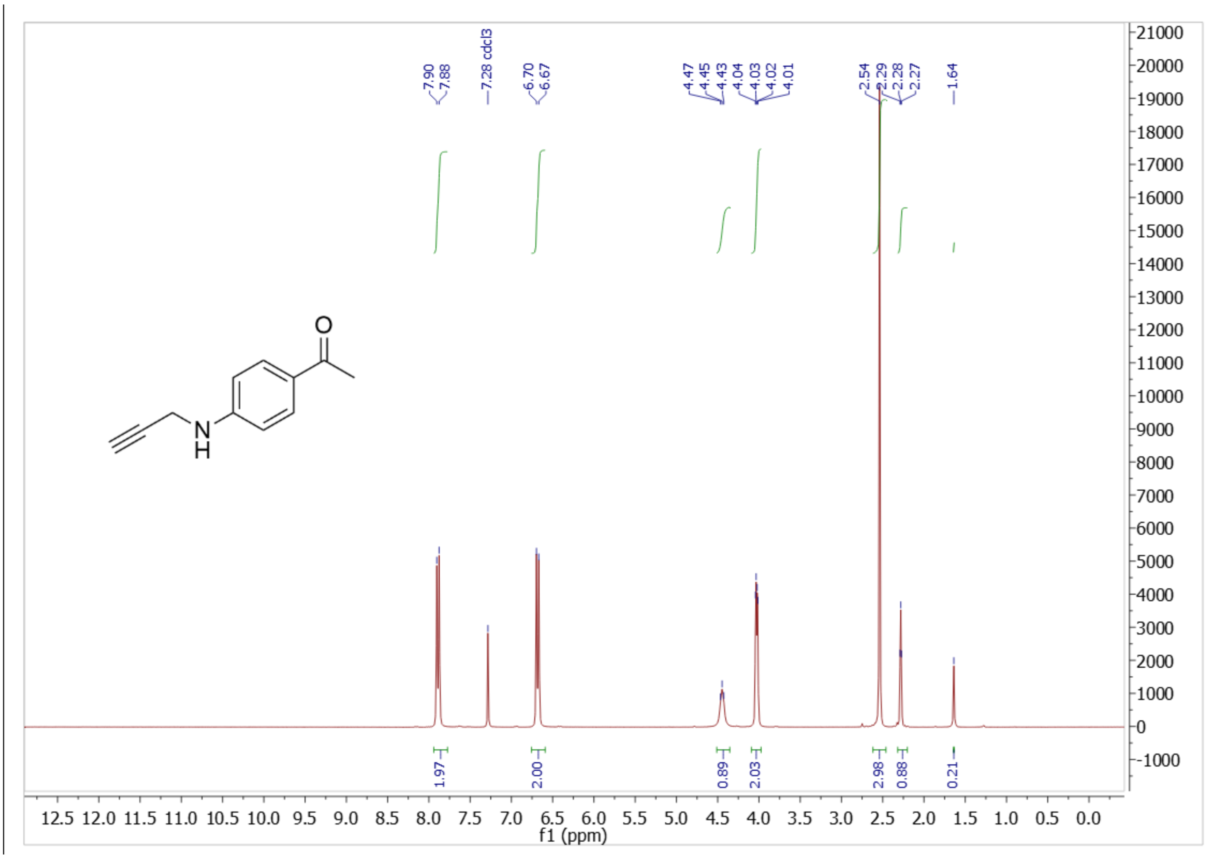

In a round bottom flask 4-methylbenzenesulfonohydrazide (0.03 g; 0.17 mmol) and 1-(4-(prop-2-yn-1-ylamino)phenyl)ethan-1-one (0.03 g; 0.17 mmol) were dissolved in 5 mL ethanol. The resulting mixture was stirred at room temperature for 5 hours. After completion of the reaction the solvent was evaporated.

Yield: 0.05 g (88%); yellow solid; m.p.: 137 °C; ^{1}H NMR (300 MHz, $CDCl_3$) δ 7.94 (d; J = 8.0 Hz; 2H), 7.57 (d; J = 8.5 Hz; 2H), 7.33 (d; J = 8.0 Hz; 2H), 6.64 (d; J = 8.7 Hz; 2H), 3.97 (d; J = 2.5 Hz; 2H), 2.43 (s; 3H), 2.25 (t; J = 2.4 Hz; 1H), 2.12 (s; 3H) ppm, ^{13}C NMR (75 MHz, $CDCl_3$) δ 153.45; 148.13; 143.99; 135.52; 129.53; 128.15; 127.69; 127.30; 112.80; 80.44; 71.54; 33.33; 21.61; 13.16 ppm. HRMS (ESI/Q-TOF) m/z: $[M+H]^+$ Calcd. for $C_{18}H_{20}N_3O_2S$ 342.1276; found 342.1268.

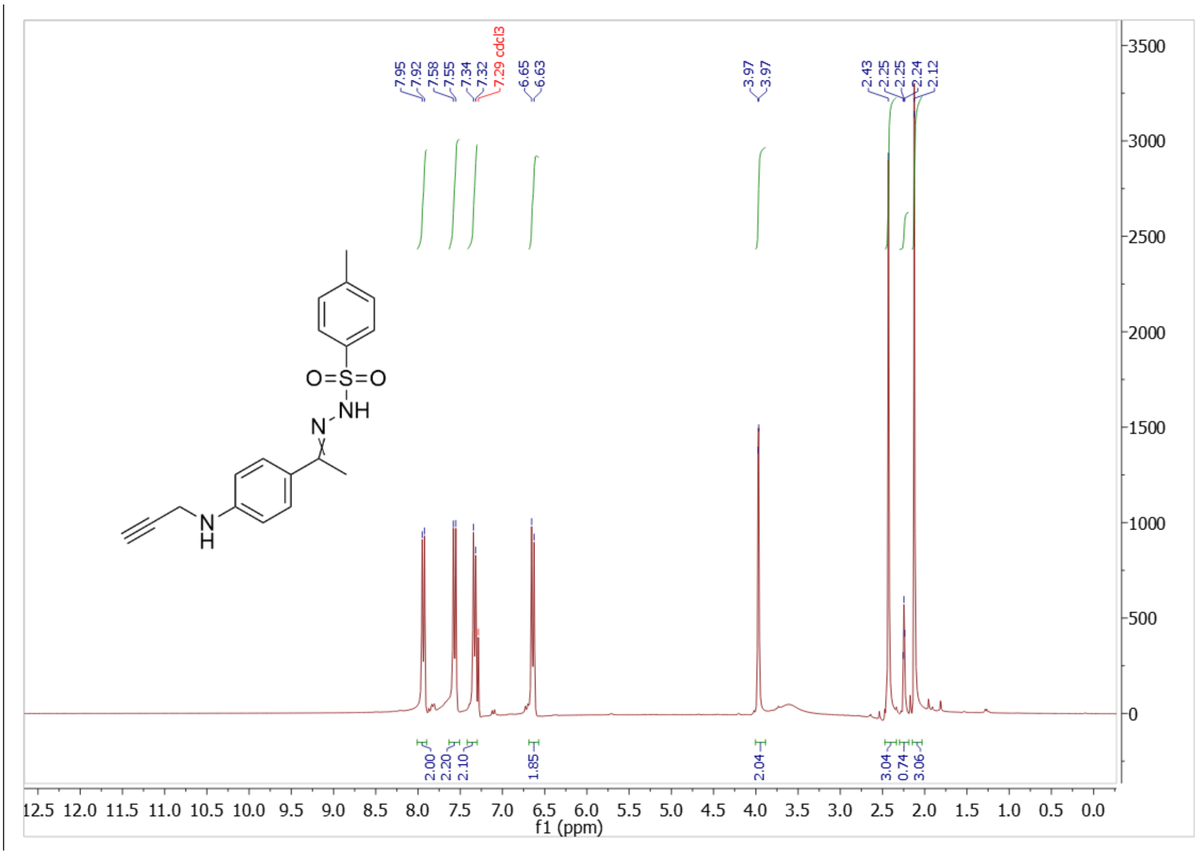

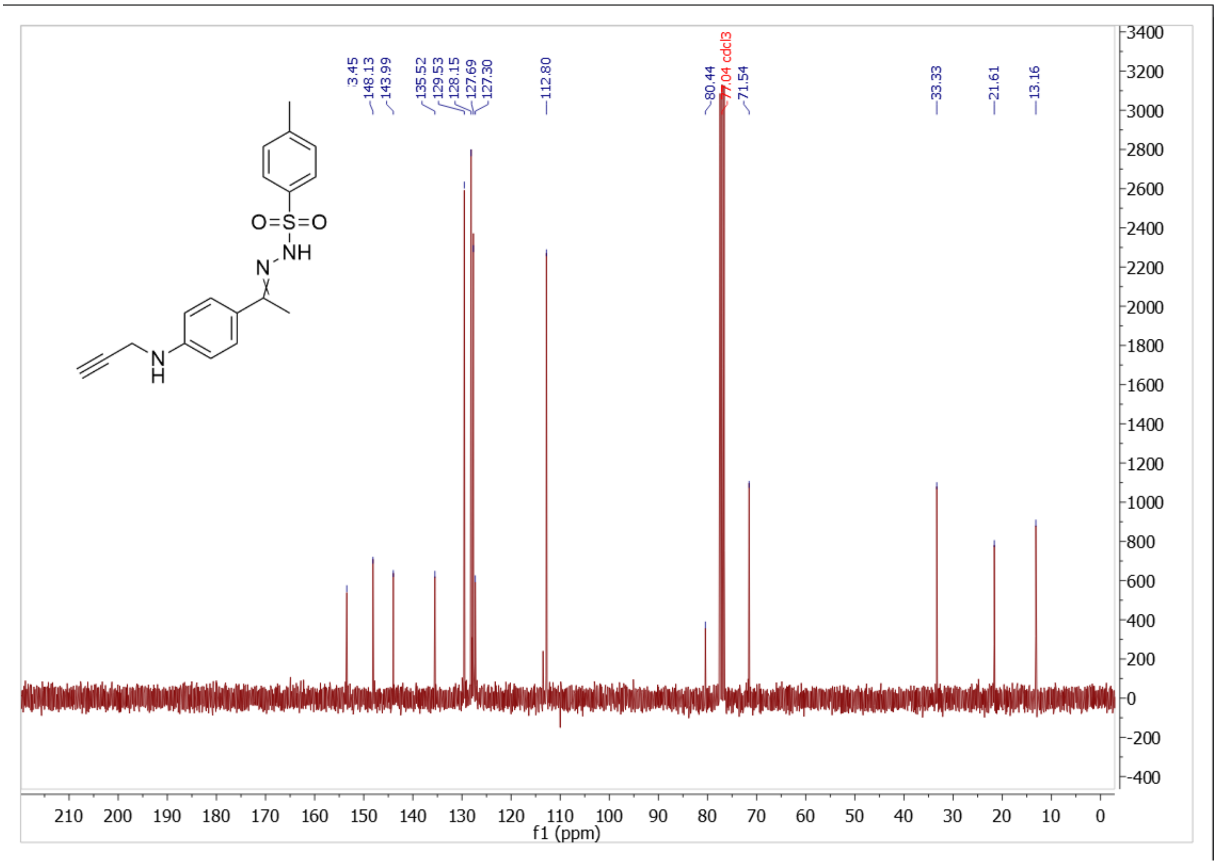

N′-(1-(4-(azidomethyl) phenyl)ethylidene)-4-methylbenzenesulfonohydrazide (**4c**):

1-(4-(Azidomethyl)phenyl)ethan-1-one

In a round bottomed flask 1-(4-(bromomethyl)phenyl)ethan-1-one (0.51 g; 2.40 mmol) and sodium azide (0.19 g; 2.88 mmol) was dissolved in 25 mL DMF. The resulting mixture was stirred at 80 °C overnight. After completion of the reaction the mixture was cooled down and 20 mL of saturated ammonium chloride was added to the mixture. The reaction was then washed with 3x30 mL of hexane:diethyl ether 1:1. The organic phase was dried over sodium sulfate and concentrated. The crude was purified by normal-phase flash chromatography.

Yield: 0.29 g (68%); pale yellow gum; ^{1}H NMR (300 MHz, DMSO-d_6) δ 7.99 (d, J = 8.1 Hz, 2H), 7.52 (d, J = 8.0 Hz, 2H), 4.57 (s, 2H), 2.59 (s, 3H).

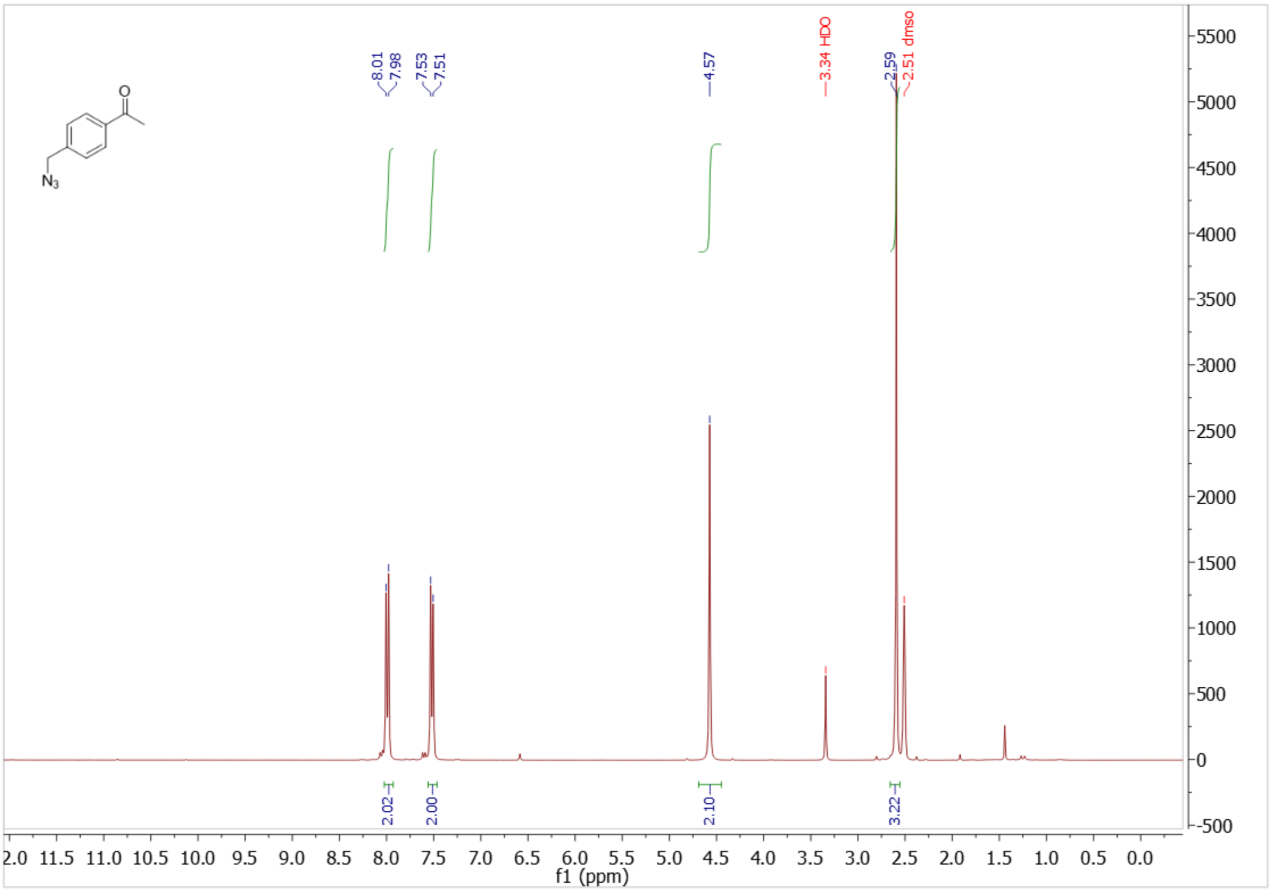

In a round bottomed flask 4-methylbenzenesulfonohydrazide (0.11 g; 0.60 mmol) and 1-(4-(azidomethyl)phenyl)ethan-1-one (0.11 g; 0.60 mmol) was dissolved in 10 mL ethanol. The resulting mixture was stirred at room temperature for 2 days. After completion of the reaction the solvent was evaporated.

Yield: 0.18 g (86%); yellow solid; m.p.: 100 °C; ^{1}H NMR (300 MHz, DMSO-d_6) δ 10.54 (s; 1H), 7.82 (d; J = 7.9 Hz; 2H), 7.65 (d; J = 7.9 Hz; 2H), 7.47 – 7.26 (m; 4H), 4.45 (s; 2H), 2.36 (s; 3H), 2.18 (s; 3H) ppm; ^{13}C NMR (75 MHz, DMSO-d_6) δ 153.09; 143.82; 137.62; 137.24; 136.65; 129.93; 128.86; 128.04; 126.75; 53.61; 21.48; 14.71 ppm; HRMS (ESI/Q-TOF) m/z: [M+H]$^+$ Calcd. for $C_{16}H_{18}N_5O_2S$ 344.1181; found 344.1180.

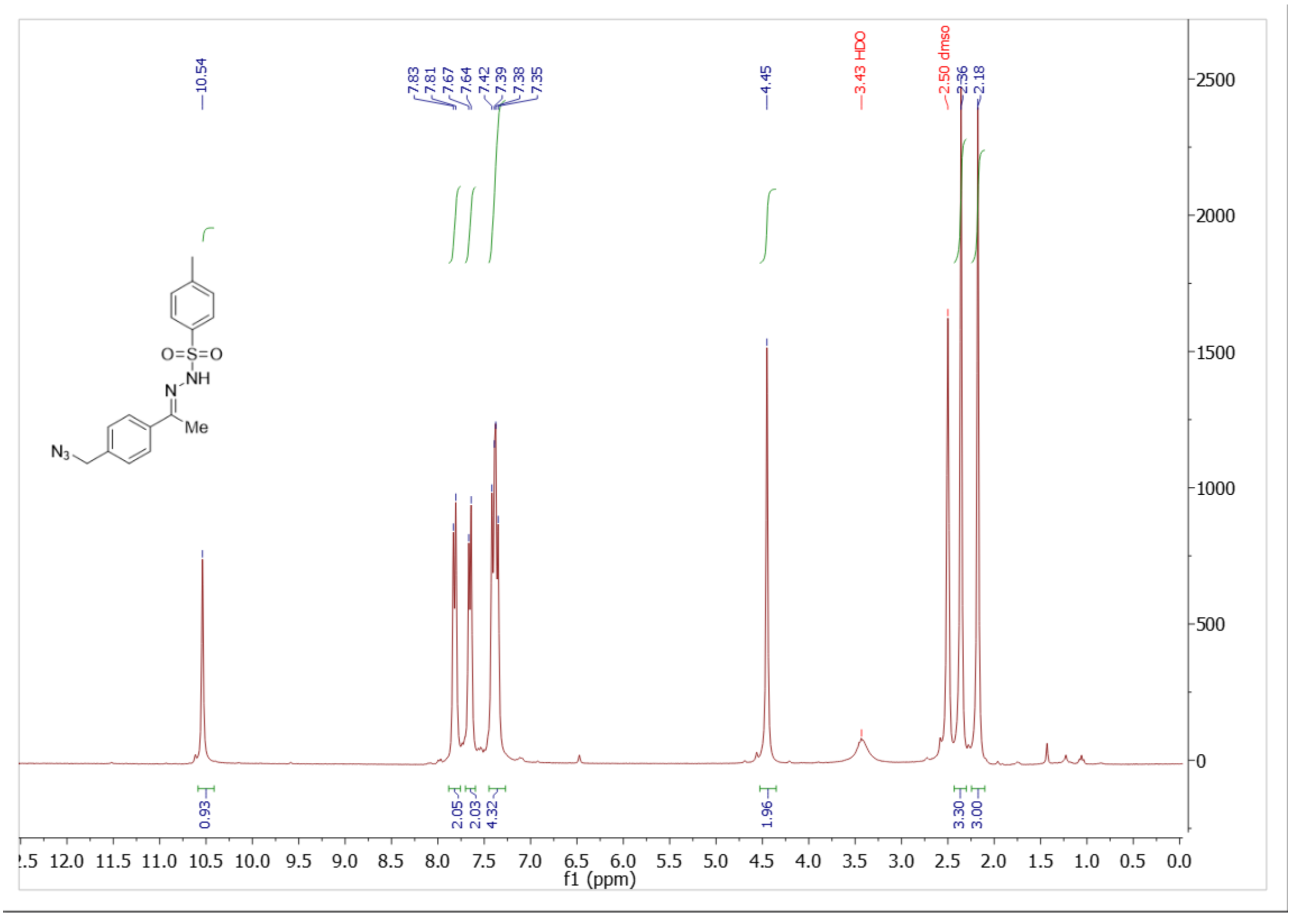

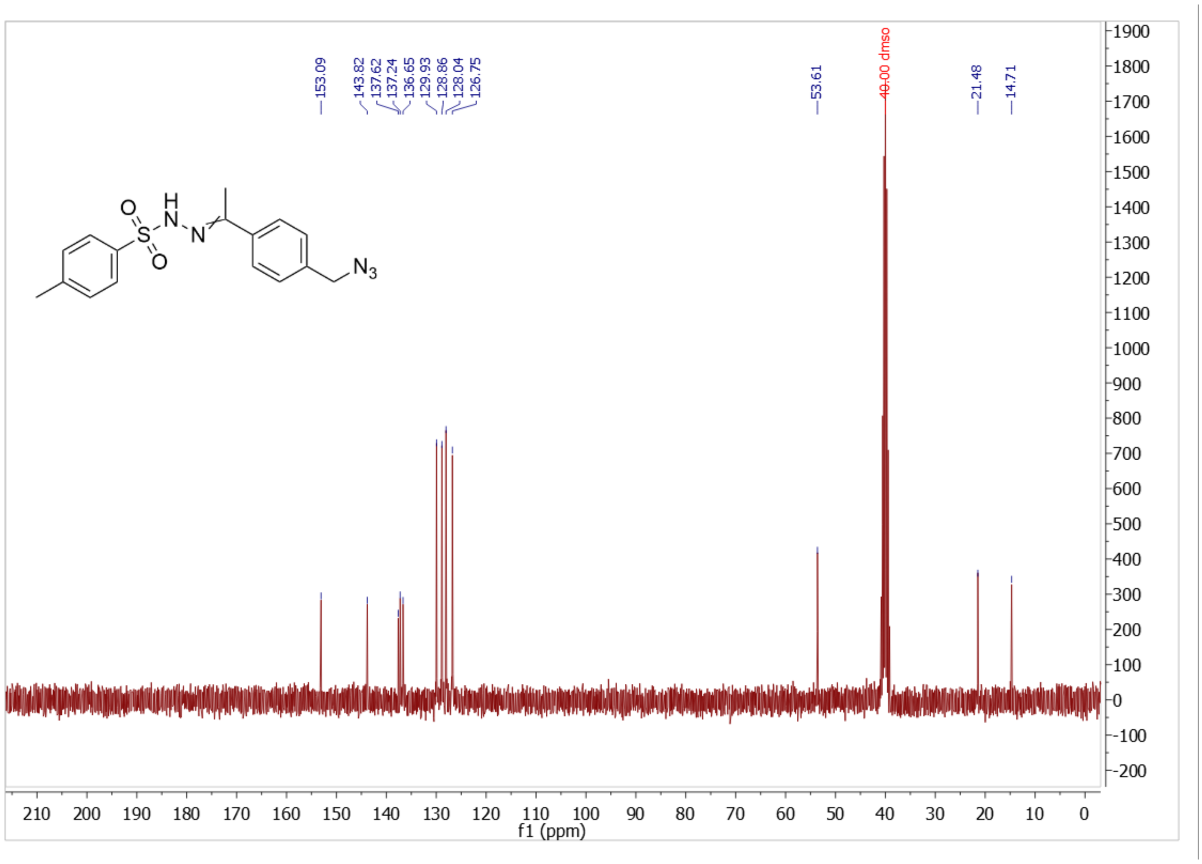

N'-(1-(4-aminophenyl)ethylidene)-4-methylbenzenesulfonohydrazide (**4e**):

In a round bottom flask 4-methylbenzenesulfonohydrazide (0.37 g; 2.00 mmol) and 4-aminoacetophenone (0.27 g; 2.00 mmol) were dissolved in 15 mL methanol. The resulting mixture was stirred at room temperature for 4 days. After completion of the reaction the product was collected by filtration.

Yield: 0.61 g (95%); white solid; ^{1}H NMR (300 MHz, DMSO-d_6) δ 10.05 (s; 1H), 7.79 (d; J = 7.9 Hz; 2H), 7.40 (d; J = 7.9 Hz; 2H), 7.33 (d; J = 8.2 Hz; 2H), 6.49 (d; J = 8.2 Hz; 2H), 5.42 (s; 2H), 2.37 (s; 3H), 2.06 (s; 3H) ppm.

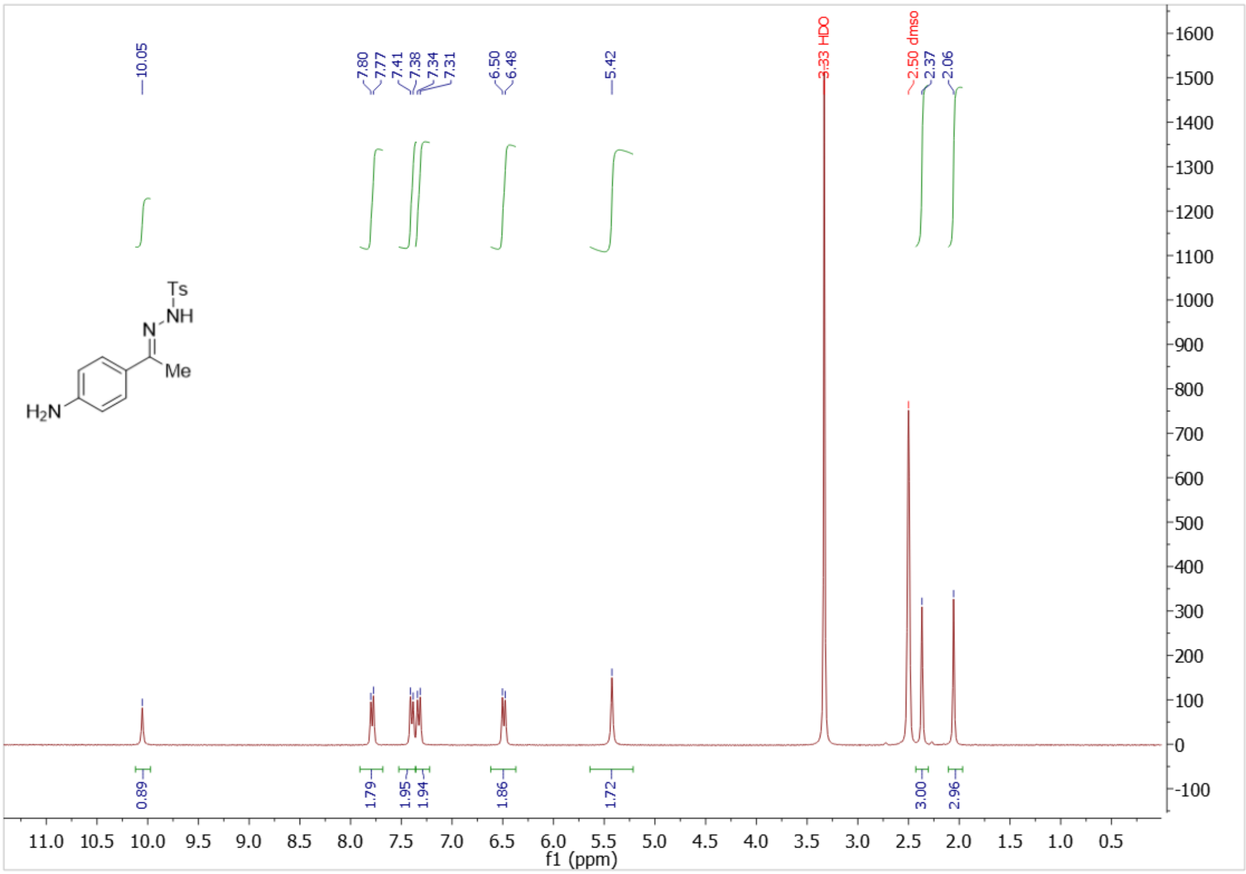

4-(1-(2-Tosylhydrazinylidene)ethyl)benzoic acid (**4f**)

In a round bottom flask 4-methylbenzenesulfonohydrazide (0.37 g; 2.00 mmol) and 4-acetylbenzoic acid (0.33 g; 2.00 mmol) were dissolved in 25 mL ethanol. The resulting mixture was stirred at room temperature for 2 days. After completion of the reaction the product was collected by filtration. The filtrate was purified by normal phase flash chromatography.

Yield: 0.05 g (62%); white solid; ^{1}H NMR (300 MHz, DMSO-d_6) δ 13.00 (s; 1H), 10.69 (s; 1H), 7.92 (d; J = 8.2 Hz; 2H), 7.81 (d; J = 8.0 Hz; 2H), 7.73 (d; J = 8.2 Hz; 2H), 7.41 (d; J = 8.0 Hz; 2H), 2.36 (s; 3H), 2.19 (s; 3H) ppm.

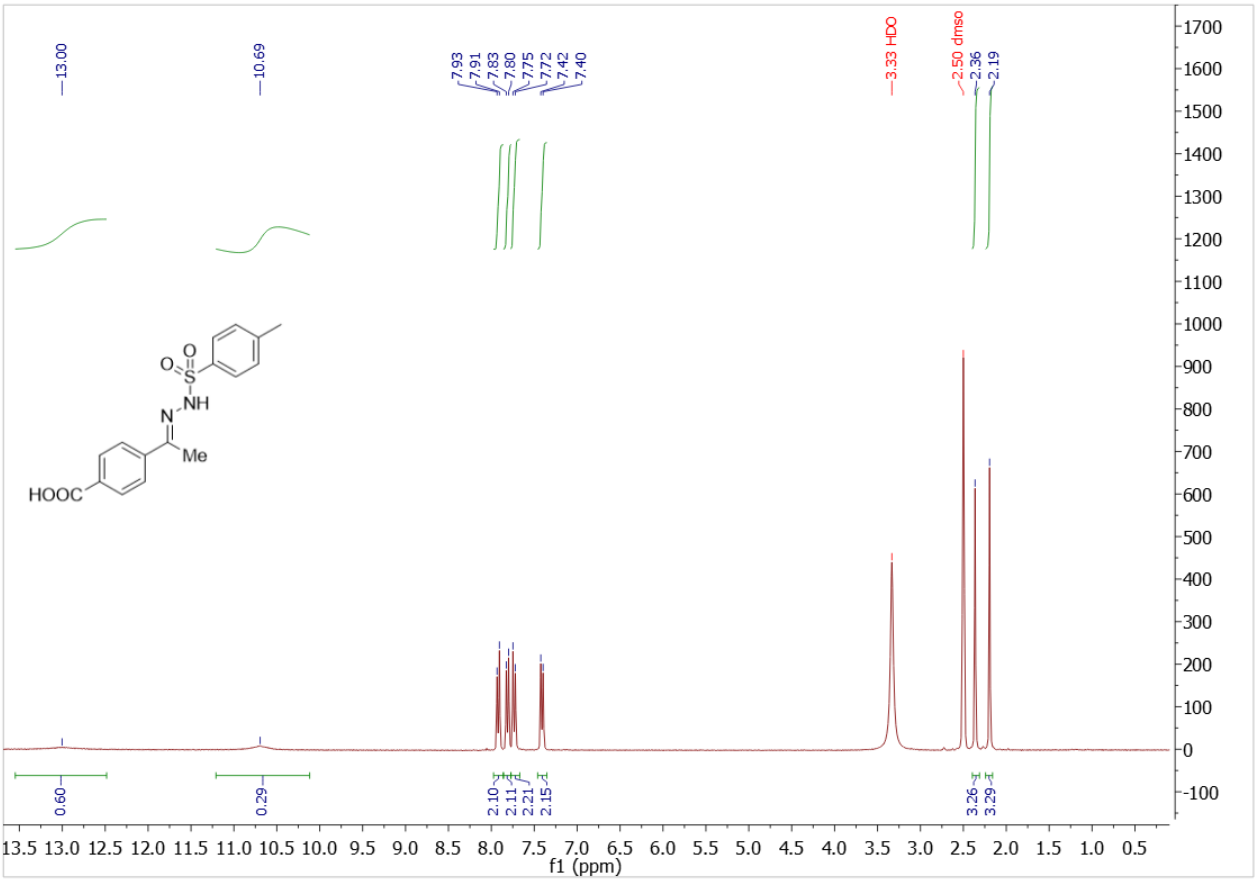

N′-(1-(4-(bromomethyl)phenyl)ethylidene)-4-methylbenzenesulfonohydrazide (**4d**):

In a round bottom flask 4-methylbenzenesulfonohydrazide (0.19 g; 1.00 mmol) and 1-(4-(bromomethyl)phenyl)ethanone (0.21 g; 1.00 mmol) were dissolved in 15 mL ethanol. The resulting mixture was stirred at room temperature for 1 day. After completion of the reaction the mixture was cooled down, and the product was collected by filtration.

Yield: 0.19 g (51%); white solid; m.p.: 140 °C; ^{1}H NMR (300 MHz, DMSO-d_6) δ 10.54 (s; 1H), 7.81 (d; J = 8.0 Hz; 2H), 7.61 (d; J = 8.0 Hz; 2H), 7.42 (m; 4H), 4.70 (s; 2H), 2.37 (s; 3H), 2.16 (s; 3H) ppm; ^{13}C NMR (75 MHz, DMSO-d_6) δ 152.96; 143.84; 139.62; 137.76; 136.63; 129.93; 129.78; 128.04; 126.73; 34.40; 21.49; 14.71 ppm; HRMS (ESI/Q-TOF) m/z: $[M+H]^+$ Calcd. for $C_{16}H_{18}BrN_2O_2S$ 381.0272; found 381.0281, 383.0260.

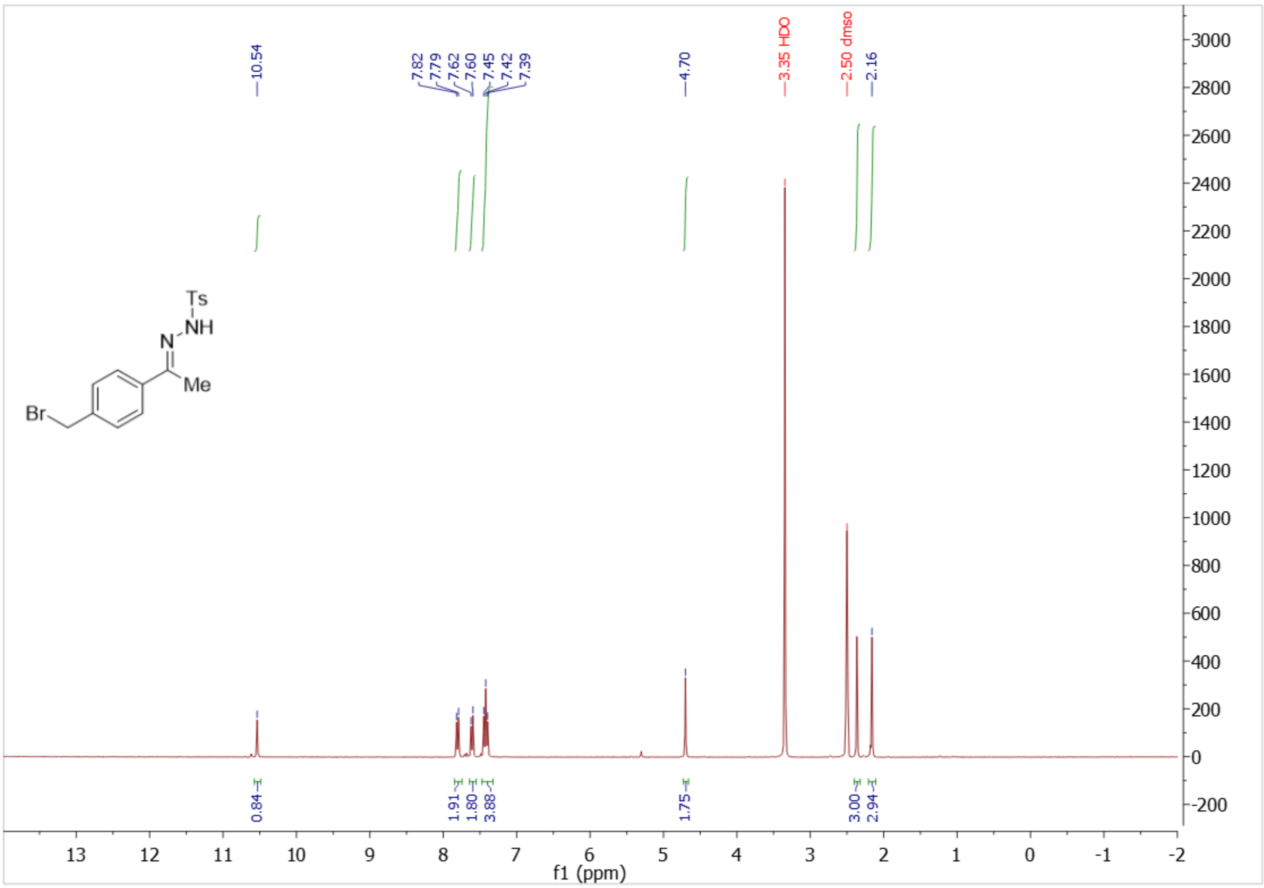
10.54
7.82
7.79
7.62
7.60
7.45
7.42
7.39
4.70
3.35 HDO
2.50 dmso
2.16
Ts
NH
N
Me
Br
0.84
1.91
1.80
3.88
1.75
3.00
2.94
f1 (ppm)

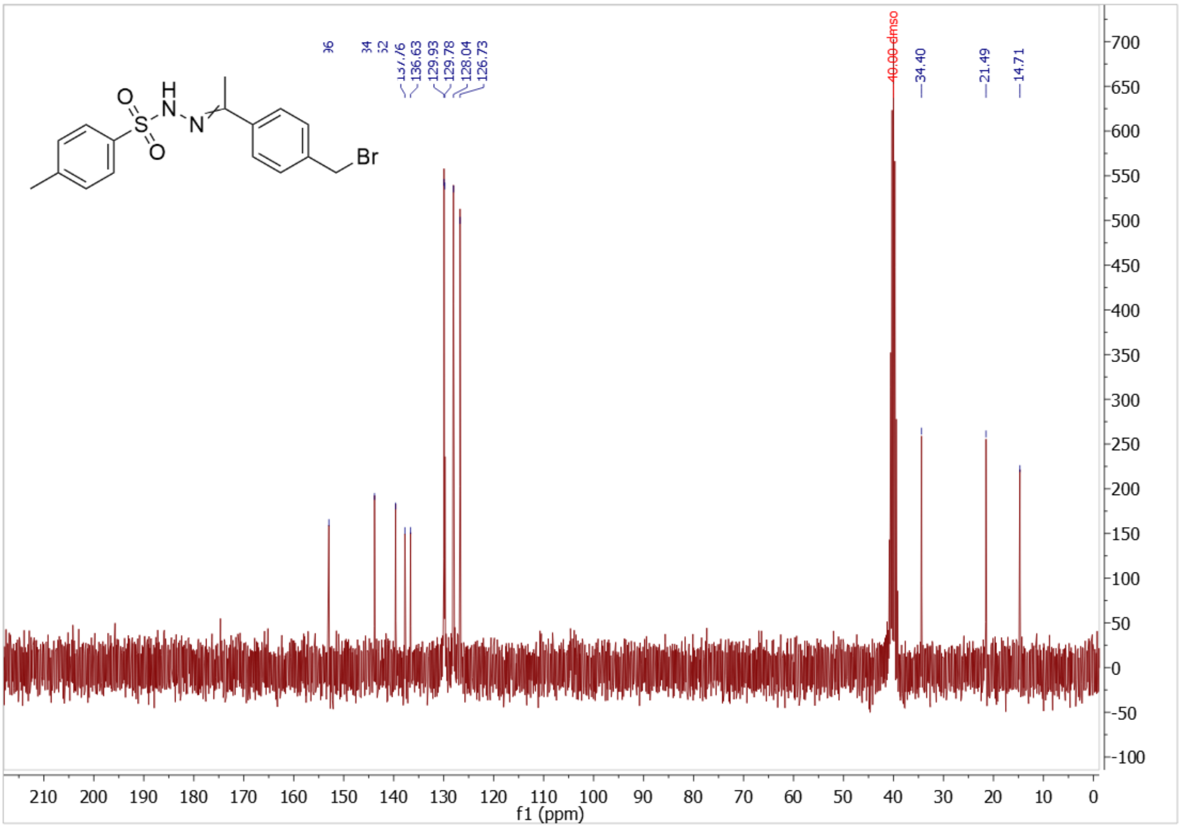
136.63
129.93
129.78
128.04
126.73
40.00 dmso
34.40
21.49
14.71
O
S
O
H
N
N
Br
f1 (ppm)

4-Methyl-N'-(1-(4-((4-phenylpiperazin-1-yl)methyl)phenyl)ethylidene)benzenesulfonohydrazide (**4g**)

In a round bottom flask 4-methylbenzenesulfonohydrazide (0.10 g; 0.26 mmol) and 1-(4-((4-phenylpiperazin-1-yl)methyl)phenyl)ethan-1-one (0.04 g; 0.26 mmol; 36.24 μL) were dissolved in 5 mL acetonitrile. The resulting mixture was stirred at room temperature for 4 hours. After completion of the reaction the mixture was filtered, and the crude was purified by normal-phase flash chromatography.

Yield: 0.06 g (52%); pale pink solid; m.p.: 172 °C; ^{1}H NMR (300 MHz, DMSO-d_6) δ 10.49 (s, 1H), 7.80 (d, J = 8.3 Hz, 2H), 7.58 (d, J = 8.1 Hz, 2H), 7.39 (d, J = 8.1 Hz, 2H), 7.31 (d, J = 8.1 Hz, 2H), 7.18 (dd, J = 8.7, 7.2 Hz, 2H), 6.96 – 6.82 (m, 2H), 6.75 (t, J = 7.2 Hz, 1H), 3.51 (s, 2H), 3.09 (dd, J = 6.5, 3.5 Hz, 4H), 2.52 – 2.37 (m, 4H), 2.35 (s, 3H), 2.16 (s, 3H); ^{13}C NMR (75 MHz, DMSO-d_6) δ 153.48 , 151.44 , 143.74 , 139.90 , 136.70 , 136.66 , 129.89 , 129.32 , 128.02 , 126.33 , 119.22 , 115.81 , 62.01 , 52.99 , 48.63 , 21.47 , 14.72 ppm; HRMS (ESI/Q-TOF) m/z: $[M+H]^+$ Calcd. for $C_{21}H_{19}N_4O_2S$ 463.2167; found 463.2173.

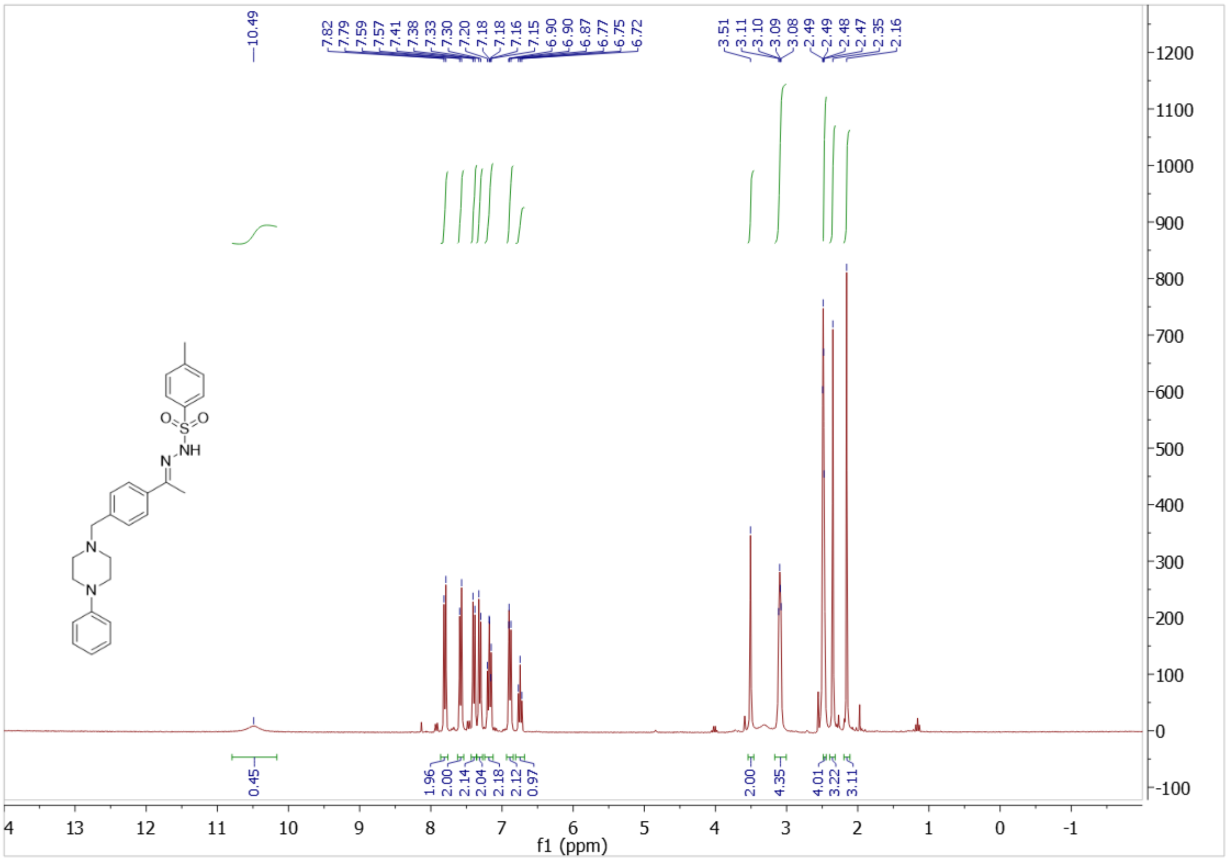

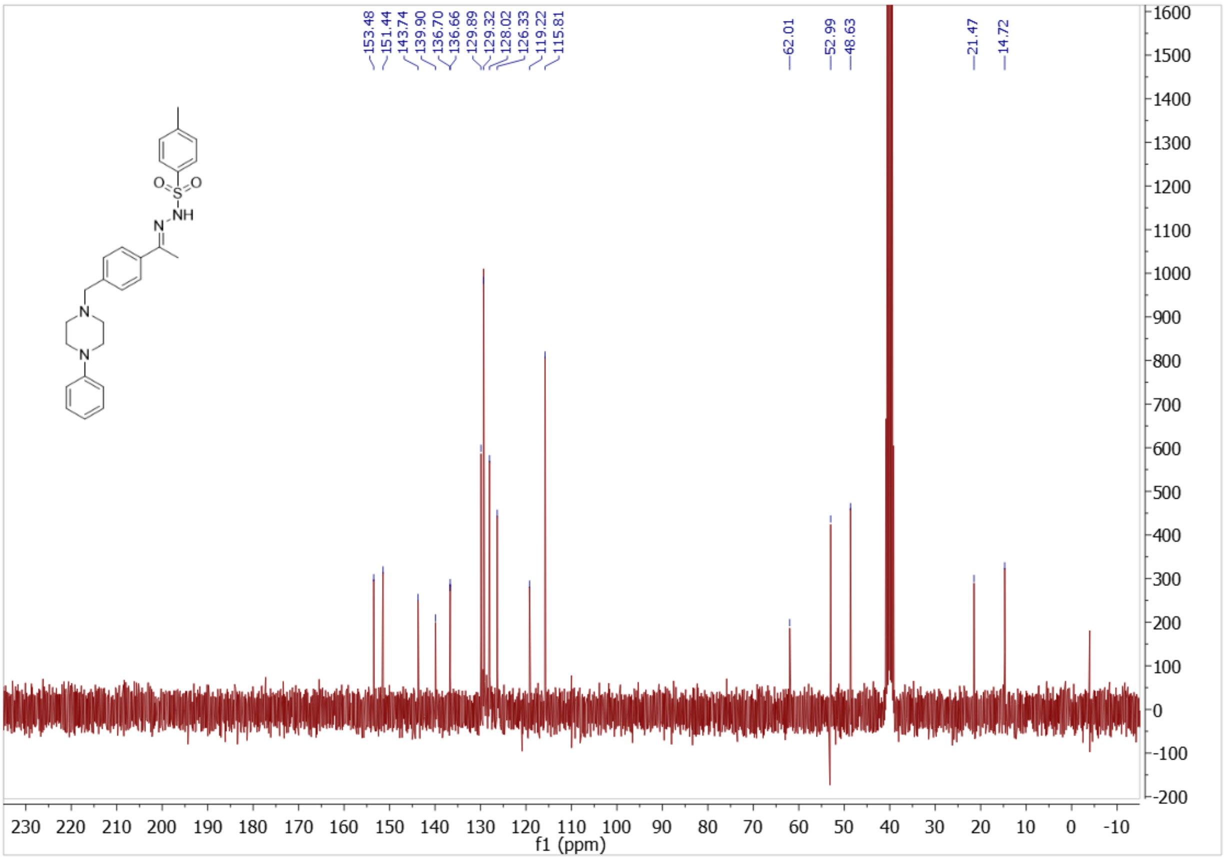

N-(4-benzoylphenyl)benzamide (**5c**)

In a round bottom flask 4-aminobenzophenone (0.20 g; 1.00 mmol) and triethylamine (0.11 g; 1.10 mmol; 0.15 mL) were dissolved in 20 mL dichloromethane. Benzoyl chloride (0.15g; 1.00 mmol; 0.13 mL) in 5 mL dichloromethane was added dropwise to the mixture. The resulting mixture was stirred at room temperature for 1 day. After completion of the reaction the mixture was washed with 20 mL distilled water, 20 mL saturated $NaHCO_3$ solution and 20 mL saturated NaCl solution. The organic phase was collected and the solvent was evaporated after drying the mixture with sodium sulfate.

Yield: 0.21 g (70%); pale brown solid; ^{1}H NMR (300 MHz, DMSO-d_6) δ 10.63 (s, 1H), 8.07 – 7.96 (m, 4H), 7.80 (d, J = 8.7 Hz, 2H), 7.77 – 7.72 (m, 2H), 7.71 – 7.47 (m, 6H).

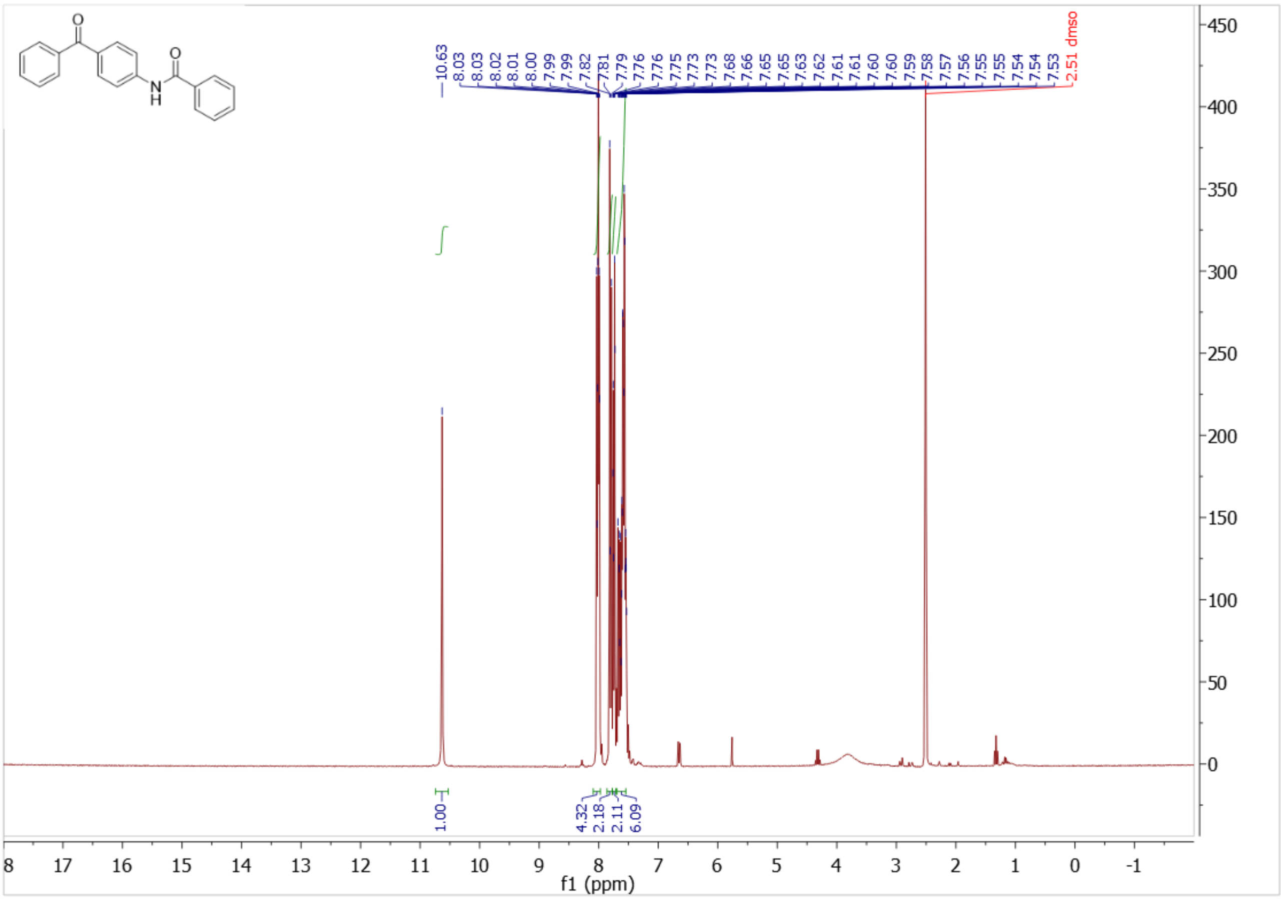

N-(4-(phenyl(2-tosylhydrazinylidene)methyl)phenyl)benzamide (**5d**)

In a round bottom flask 4-methylbenzenesulfonohydrazide (0.09 g; 0.50 mmol) and *N*-(4-benzoylphenyl)benzamide (0.15 g; 0.50 mmol) and catalytic amount of p-toluenesulfonic acid were dissolved in 20 mL ethanol. The resulting mixture was stirred at 80 °C for 6 hours. After completion of the reaction the mixture was cooled down, and the product was collected by filtration.

Yield: 0.05 g (20%); white solid; m.p.: 185°C ^{1}H NMR (300 MHz, DMSO-d_6) δ 10.38 (s, 1H), 10.30 (s, 1H), 8.10 – 7.90 (m, 2H), 7.83 (d, J = 8.0 Hz, 2H), 7.76 (d, J = 8.6 Hz, 2H), 7.59 (d, J = 7.0 Hz, 1H), 7.54 (q, J = 3.0, 2.2 Hz, 5H), 7.45 (d, J = 8.0 Hz, 2H), 7.24 (dd, J = 8.1, 5.9 Hz, 4H), 2.41 (s, 3H) ppm; ^{13}C NMR (75 MHz, DMSO-d_6) δ 166.10 , 154.91 , 143.74 , 141.02 , 136.54 , 135.16 , 133.06 , 132.70 , 132.15 , 129.86 (2 peaks) , 129.25 (2 peaks), 128.83 , 128.25 , 128.17 (2 peaks), 120.23 , 21.52 ppm; HRMS (ESI/Q-TOF) m/z: [M+H]$^+$ Calcd. for $C_{27}H_{24}N_3O_3S$ 470.1538; found 470.1542.

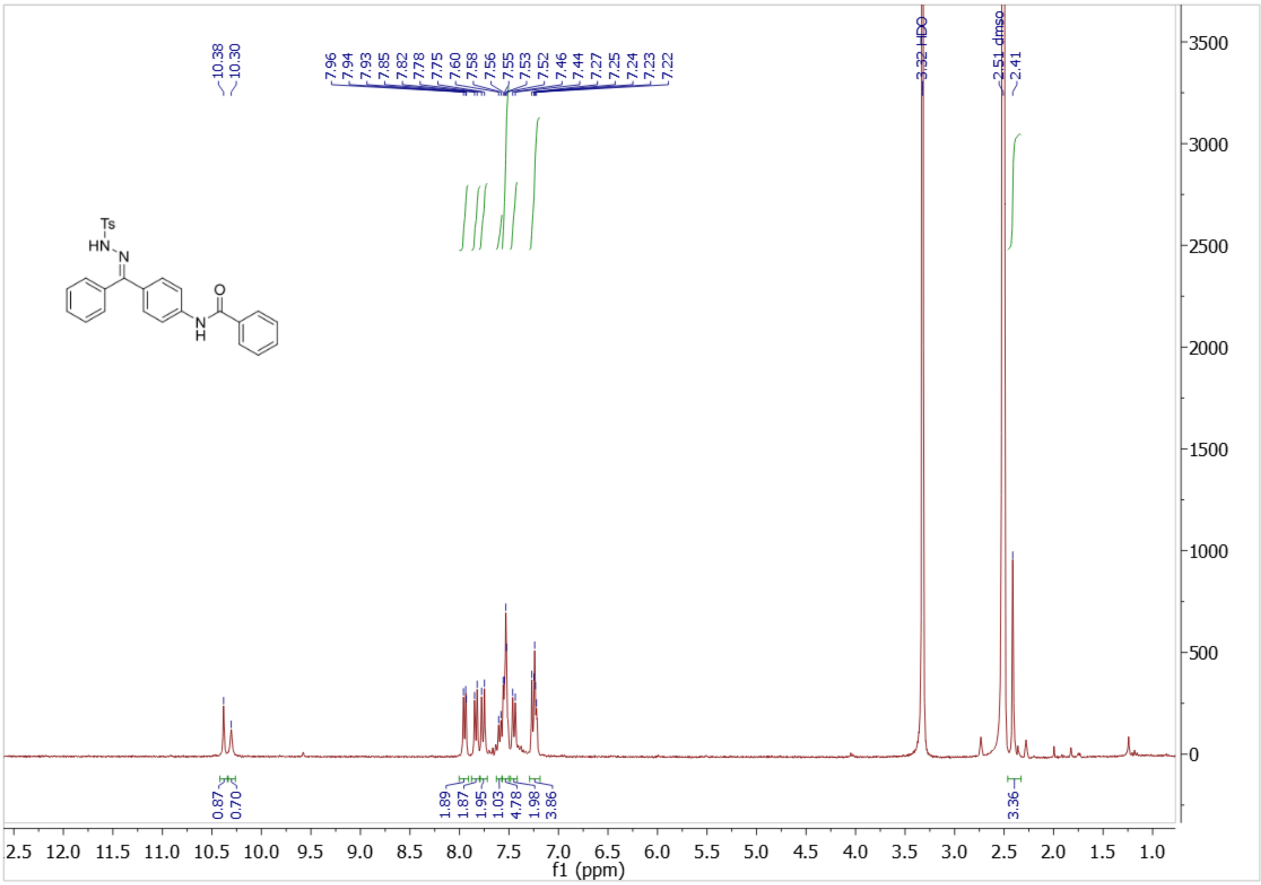

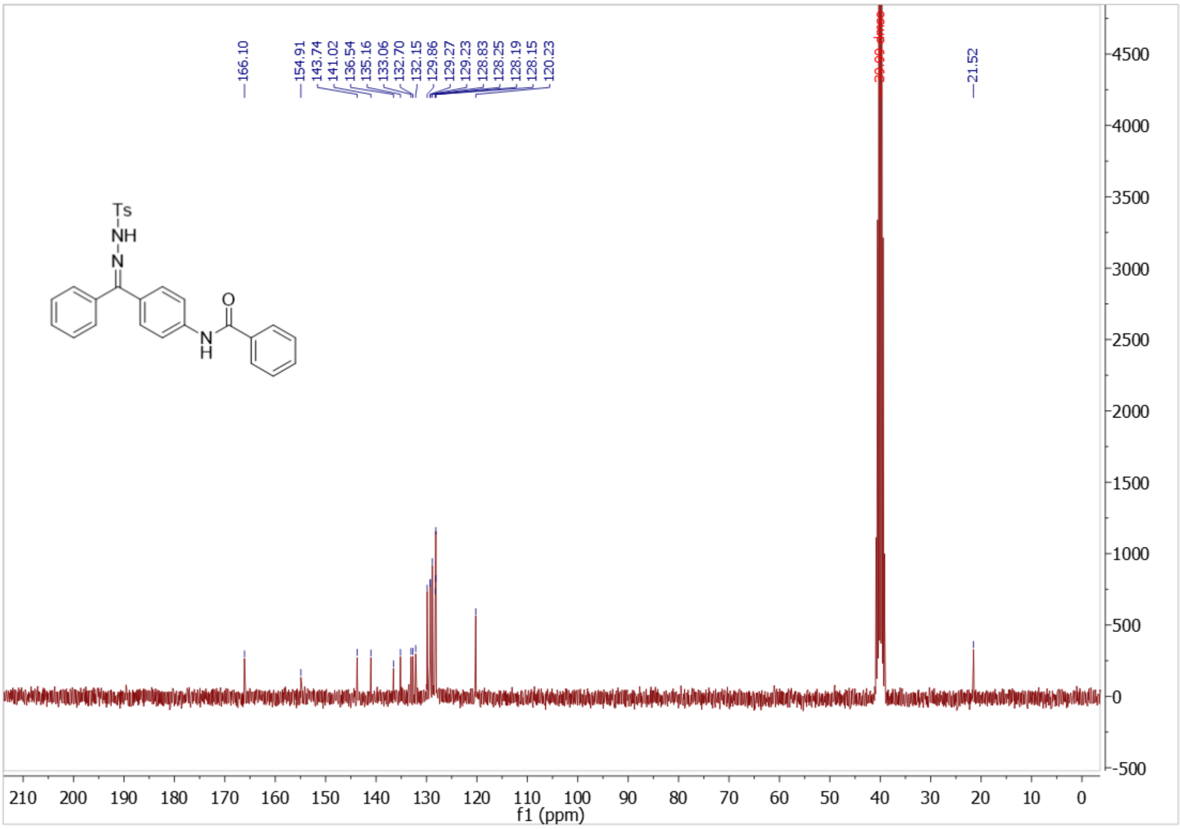

N'-(4-hydroxy-3-methoxybenzylidene)-4-methylbenzenesulfonohydrazide (**6**):

In a round bottom flask 4-methylbenzenesulfonohydrazide (0.19 g; 1.00 mmol) and vaniline (0.15 g; 1.00 mmol) were dissolved in 15 mL ethanol. The resulting mixture was stirred at room temperature for 4 hours. After completion of the reaction the solvent was evaporated from the mixture.

Yield: 0.22 g (69%); pale brown gum; ^{1}H NMR (300 MHz, DMSO-d_6) δ 11.13 (s; 1H), 9.50 (s; 1H), 7.77 (d; J = 3.5 Hz; 2H), 7.74 (s; 1H), 7.40 (d; J = 8.0 Hz; 2H), 7.09 (s; 1H), 6.96 (dd; J = 8.2, 1.9 Hz; 1H), 6.76 (dd; J = 8.1, 1.8 Hz; 1H), 3.77 (s; 3H), 2.35 (s; 3H) ppm.

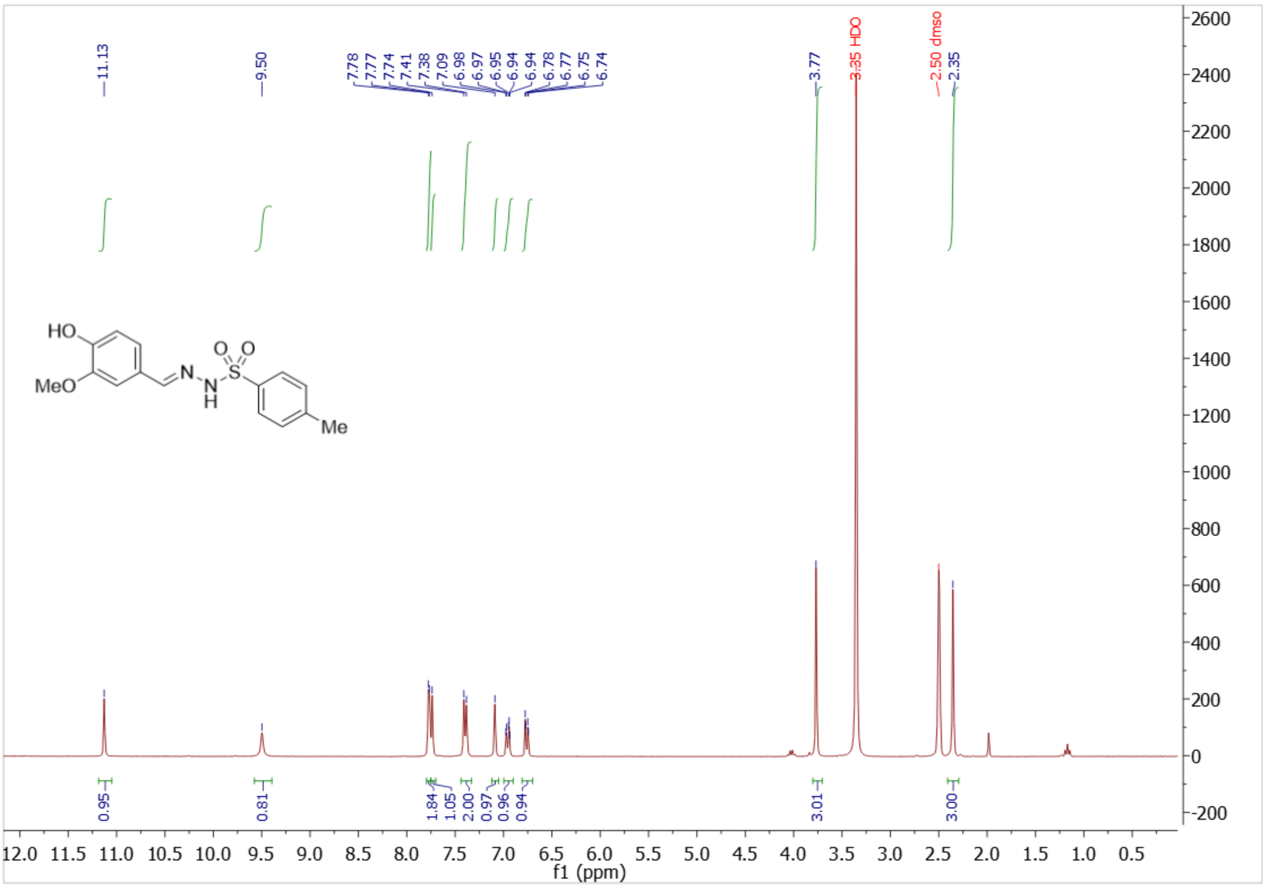

N′-((4-hydroxyphenyl)(phenyl)methylene)-4-nitrobenzenesulfonohydrazide (**7**):

In a round bottom flask 4-nitrobenzenesulfonylhydrazide (0.22 g; 1.00 mmol) and 4-hidroxybenzophenone (0.20 g; 1.00 mmol) were dissolved in 15 mL ethanol. The resulting mixture was stirred at room temperature for 3 hours. After the solvent was evaporated, and the mixture was dissolved in 15 mL 1M HCl. The resulting mixture was stirred at room temperature for 2 days. After completion of the reaction the product was collected by filtration.

Yield: 0.28 g (69%); yellow solid; ^{1}H NMR (300 MHz, DMSO-d_6) δ 10.77 (s, 1H), 9.90 (s, 1H), 8.53 – 8.40 (d, 2H), 8.24 – 8.11 (d, 2H), 7.44 – 7.19 (m, 5H), 7.17 – 7.02 (d, 2H), 6.98 – 6.82 (d, 2H).

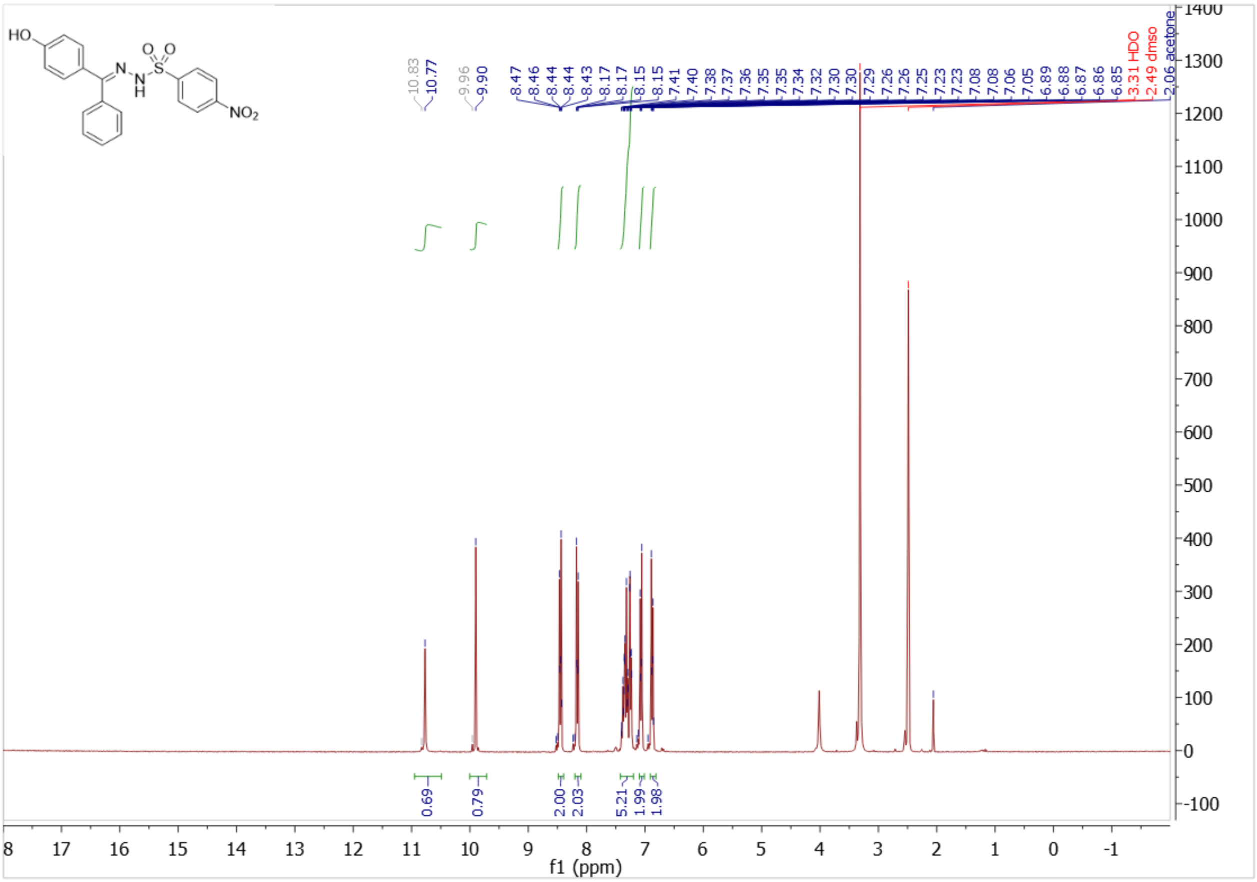

4-Methyl-*N′*-((4-oxo-4a,8a-dihydro-4H-chromen-3-yl)methylene)benzenesulfonohydrazide (**8**):

In a round bottom flask 4-methylbenzenesulfonohydrazide (0.19 g; 1.00 mmol) and 4-oxo-4H-chromene-3-carbaldehyde (0.15 g; 1.00 mmol) were dissolved in 15 mL ethanol. The resulting mixture was stirred at 70 °C for 5 hours. After completion of the reaction the mixture was cooled down, and the product was collected by filtration.

Yield: 0.34 g (99%); white solid; ^{1}H NMR (300 MHz, DMSO-d_6) δ 11.53 (s, 1H), 8.63 (s, 1H), 8.12 – 7.92 (m, 2H), 7.90 – 7.74 (m, 3H), 7.69 (d, *J* = 8.6 Hz, 1H), 7.53 (t, *J* = 7.8 Hz, 1H), 7.42 (d, *J* = 7.9 Hz, 2H), 2.37 (s, 3H).

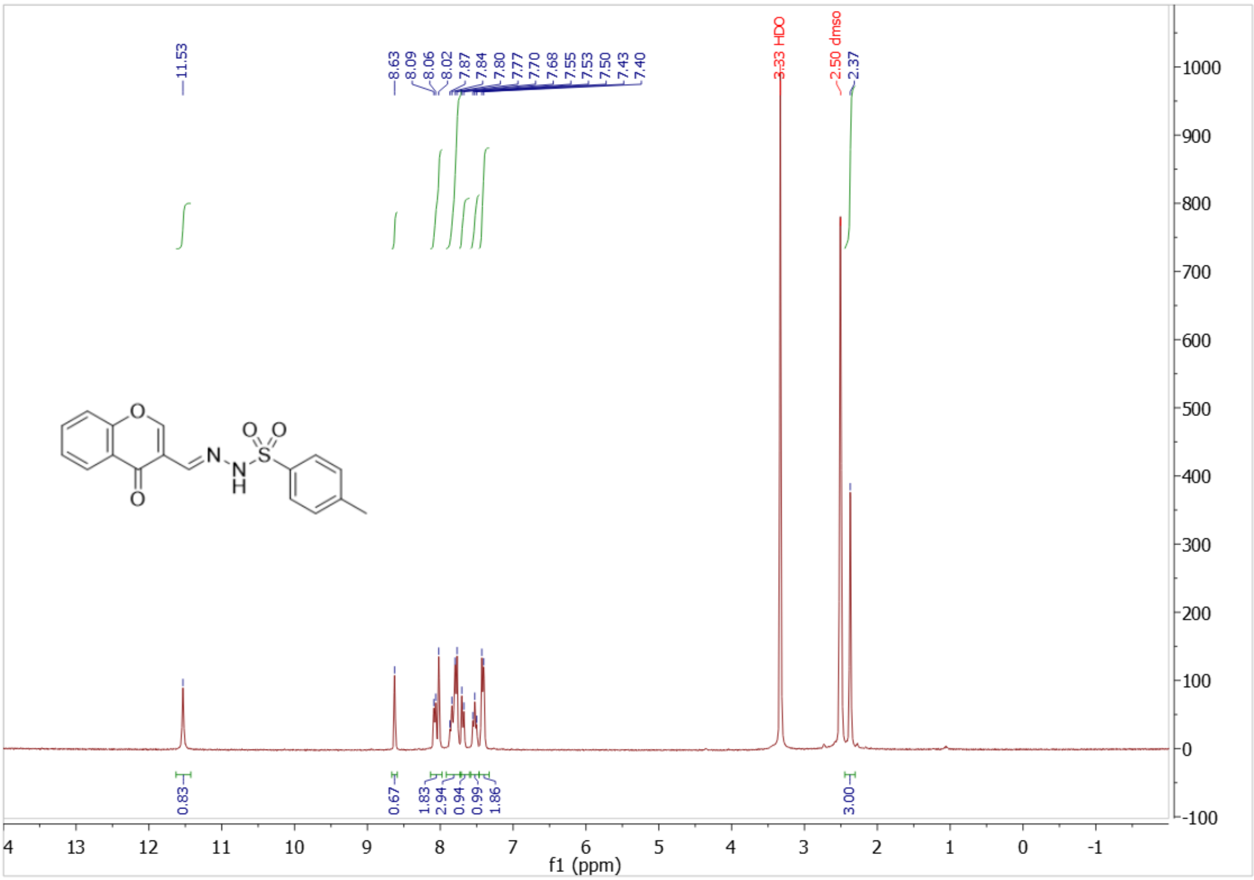

N′-(1-(1H-indol-2-yl)ethylidene)-5-bromo-2-methoxybenzenesulfonohydrazide (**9**)

5-Bromo-2-methoxybenzenesulfonohydrazide

In a round bottom flask hydrazine hydrate (0.06 g; 1.20 mmol; 0.58 mL) was dissolved in 30 mL distilled water and cooled down. To the mixture 5-bromo-2-methoxybenzenesulfonylchloride (0.34 g; 1.20 mmol) was added dropwise in 5 mL THF. The resulting mixture was stirred at room temperature for 3 hours. After completion of the reaction the product was collected by filtration.

Yield: 0.14 g (42%); white solid; ^{1}H NMR (300 MHz, DMSO-d_6) δ 8.17 (d, J = 2.9 Hz, 1H), 7.78 (d, J = 2.8 Hz, 2H), 7.43 – 6.89 (m, 1H), 4.21 (s, 2H), 3.89 (s, 3H).

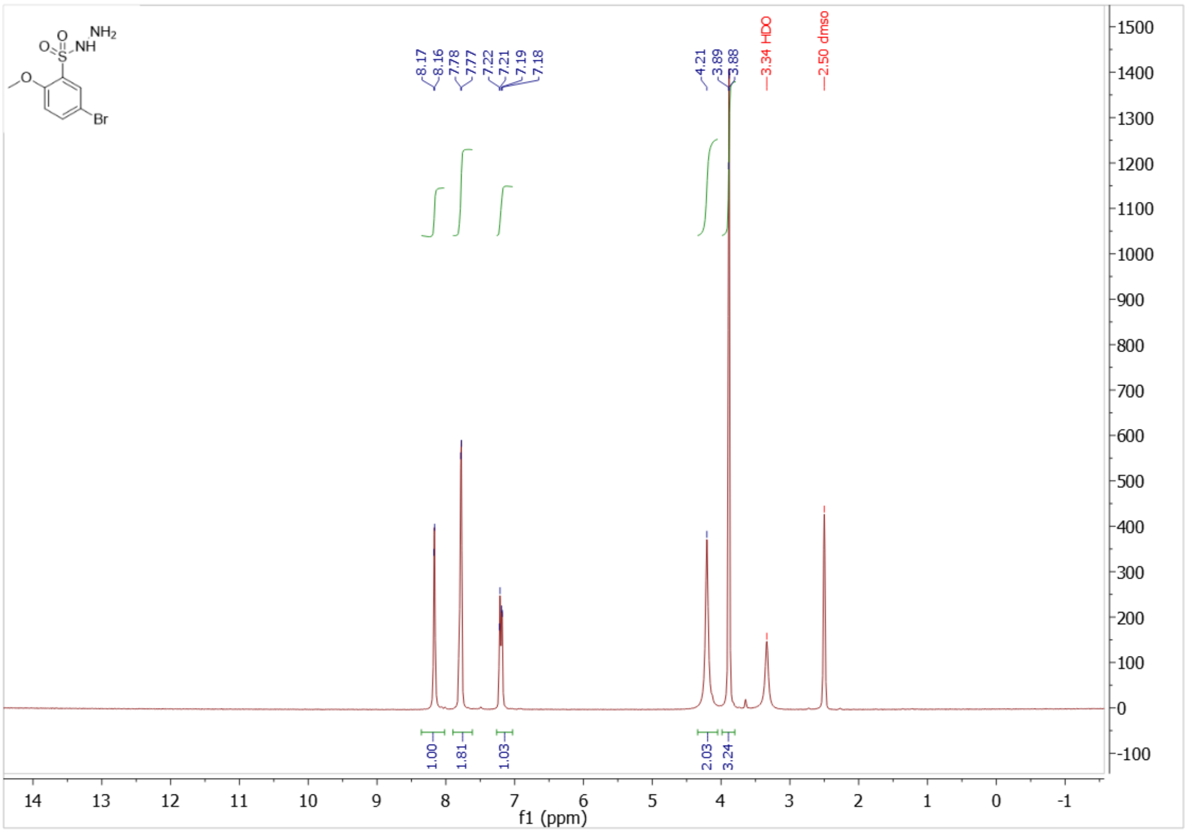

In a round bottom flask 5-bromo-2-methoxybenzenesulfonohydrazide (0.06 g; 0.36 mmol) and 1-(1H-indol-2-yl)ethan-1-one (0.10 g; 0.36 mmol) were dissolved in 10 mL ethanol. The resulting mixture was stirred at room temperature for 2 days. After completion of the reaction the product was collected by filtration.

Yield: 0.05 g (34%); white solid; ^{1}H NMR (300 MHz, DMSO-d_6) δ 10.79 (s; 1H), 10.49 (s; 1H), 7.95 (s; 1H), 7.77 (d; J = 8.5 Hz; 1H), 7.49 (d; J = 7.8 Hz; 1H), 7.39 (d; J = 7.9 Hz; 1H), 7.18 (d; J = 8.8 Hz; 1H), 7.10 (t; J = 7.2 Hz; 1H), 6.96 (t; J = 7.7 Hz; 1H), 6.81 (s; 1H), 3.89 (s; 3H), 2.28 (s; 3H) ppm.

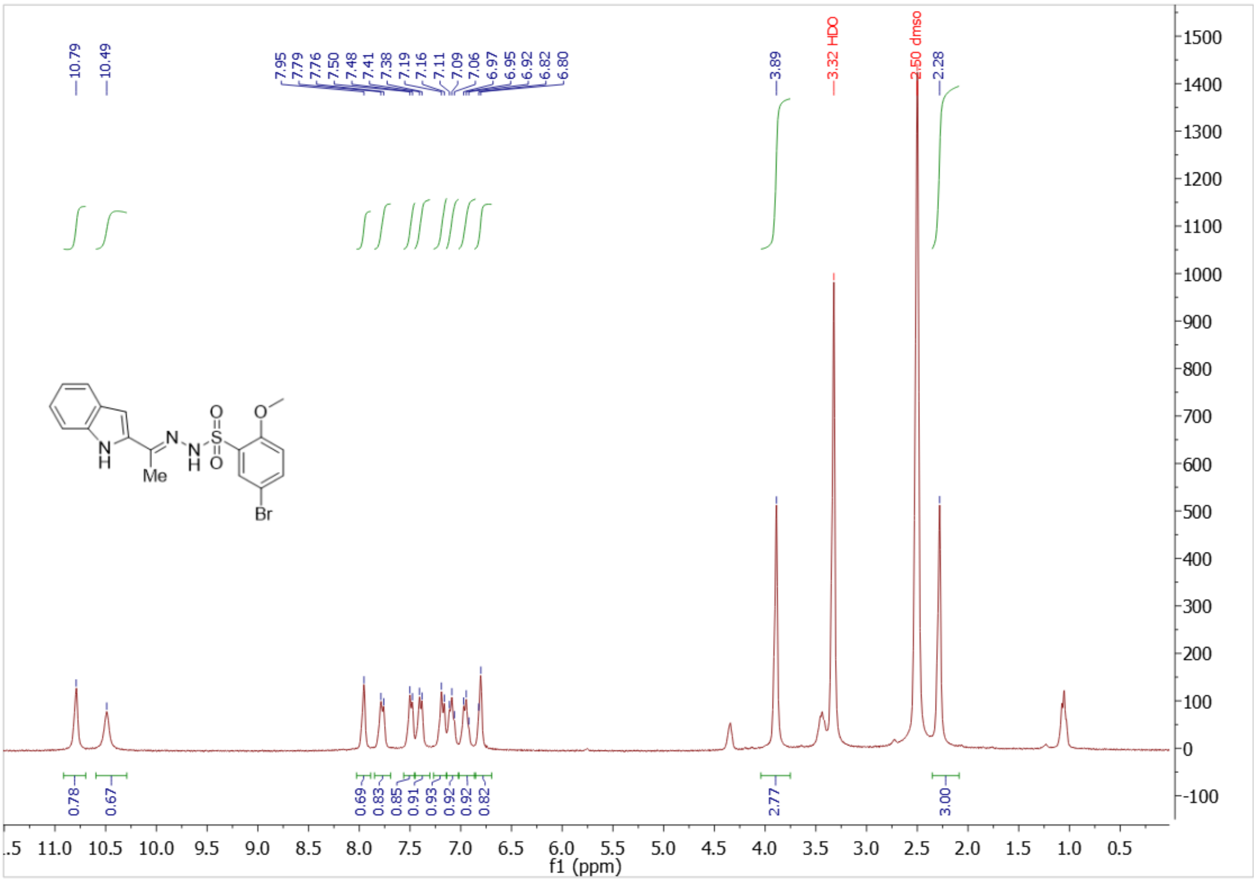

N′-((4-oxo-4a,8a-dihydro-4H-chromen-3-yl)methylene)pyridine-3-sulfonohydrazide (**10**):

Pyridine-3-sulfonohydrazide

In a round bottom flask hydrazine (0.06 g; 1.20 mmol; 0.58 mL) was dissolved in 30 mL distilled water and cooled down. To the mixture pyridine-3-sulfonyl chloride (0.20 g; 1.15 mmol) was added dropwise in 5 mL THF. The resulting mixture was stirred at room temperature for 2 hours. After completion of the reaction the solvent was evaporated, the mixture was dissolved in 20 mL dichloromethane. The organic phase was then washed with distilled water, dried over sodium sulfate and concentrated.

Yield: 0.18 g (51%); white solid; ^{1}H NMR (300 MHz, DMSO-*d6*) δ 8.63 (d; *J* = 2.0 Hz; 1H), 8.49 (dd; *J* = 4.8, 1.6 Hz; 1H), 7.83 (dt; *J* = 7.8, 1.8 Hz; 1H), 7.38 (dd; *J* = 7.7, 4.8 Hz; 1H) ppm.

In a round bottom flask pyridine-3-sulfonohydrazide (0.08 g; 0.45 mmol) and 4-oxo-4a,8a-dihydro-4H-chromene-3-carbaldehyde (0.08 g; 0.45 mmol) were dissolved in 10 mL ethanol. The resulting mixture was stirred at room temperature for 2 hours. After completion of the reaction the mixture was cooled down, and the product was collected by filtration.

Yield: 0.12 g (75%); pale yellow solid; m.p.: 192 °C; ^{1}H NMR (300 MHz, DMSO-d_6) δ 11.82 (s; 1H), 9.05 (d; J = 2.3 Hz; 1H), 8.86 (dd; J = 4.8, 1.6 Hz; 1H), 8.71 (s; 1H), 8.30 (dt; J = 8.1, 1.9 Hz; 1H), 8.13 – 8.02 (m; 2H), 7.84 (ddd; J = 8.7, 7.1, 1.7 Hz; 1H), 7.73 – 7.61 (m; 2H), 7.53 (ddd; J = 8.1, 7.1, 1.1 Hz; 1H) ppm; ^{13}C NMR (75 MHz, DMSO-d_6) δ 175,09; 156,16; 155,30; 154,14; 147,98; 141,25; 135,84; 135,74; 135,19; 126,59; 125,63; 124,89; 123,75; 119,16; 118,06 ppm; HRMS (ESI/Q-TOF) m/z: $[M+H]^+$ Calcd. for $C_{15}H_{14}N_3O_4S$ 330.0548; found 330.0550.

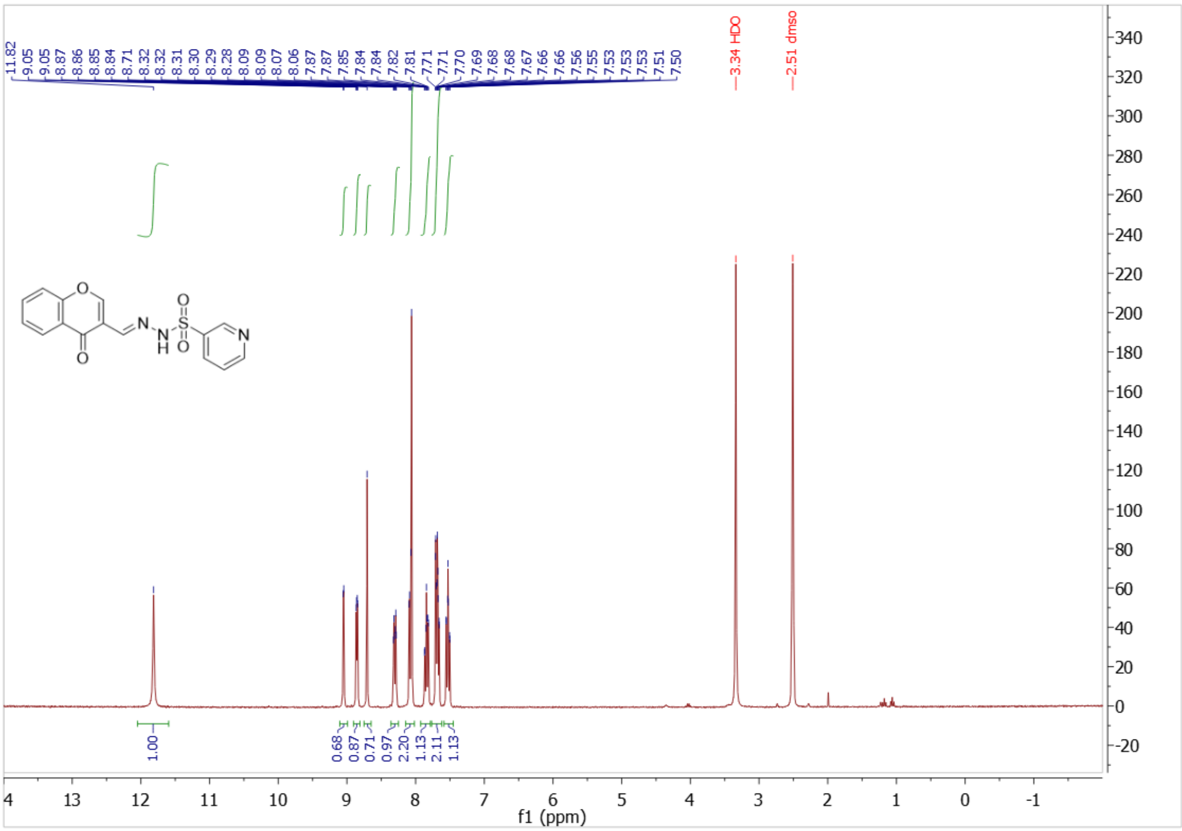

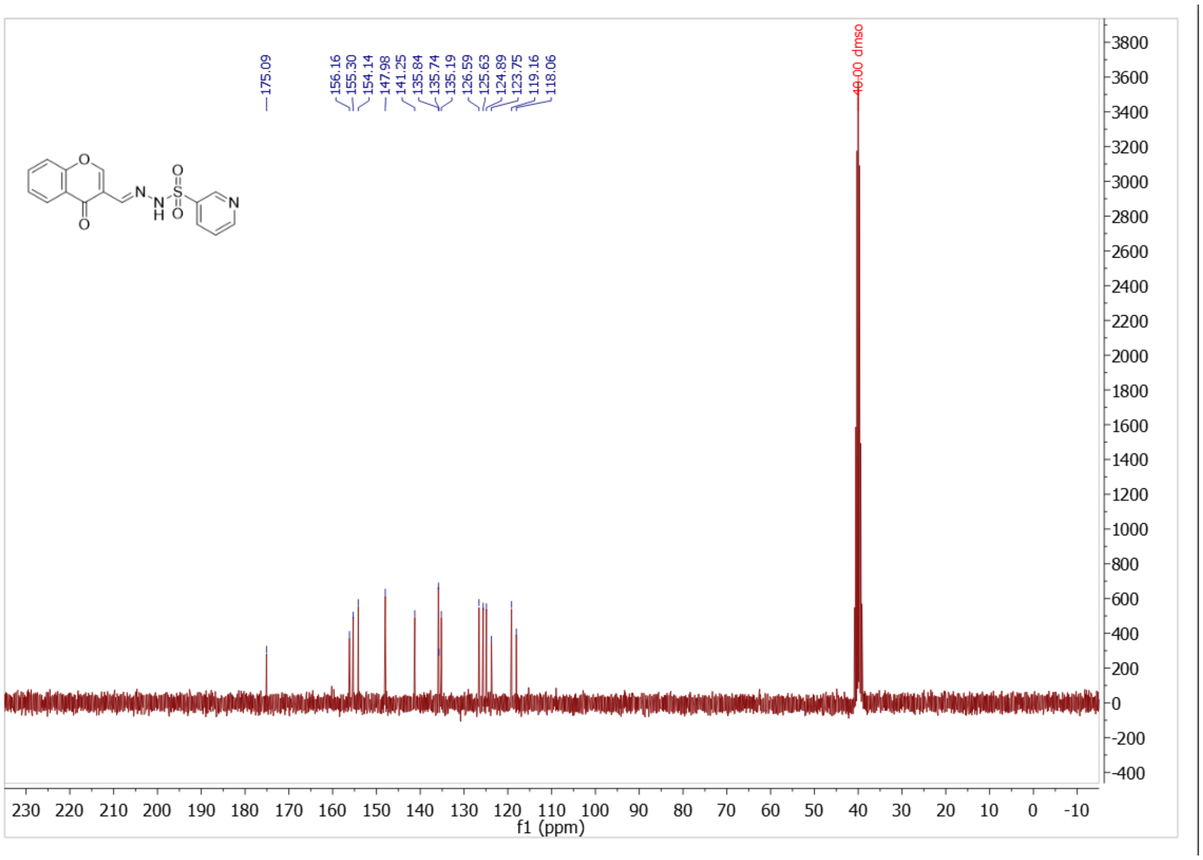

N'-((2-hydroxynaphthalen-1-yl)methylene)-4-methylbenzenesulfonohydrazide (**11**)

In a round bottom flask 4-methylbenzenesulfonohydrazide (0.09 g; 0.50 mmol) and 1-(2-hydroxynaphthalen-1-yl)ethan-1-one (0.09 g; 0.50 mmol) were dissolved in 5 mL methanol. The resulting mixture was stirred at 60 °C for 2 hours. After completion of the reaction the solvent was evaporated.

Yield: 0.17 g (100%); pale yellow solid; ^{1}H NMR (300 MHz, DMSO-d_6) δ 11.51 (s, 1H), 11.11 (s, 1H), 8.78 (s, 1H), 8.33 (dd, J = 8.7, 1.1 Hz, 1H), 7.91 – 7.71 (m, 4H), 7.55 – 7.39 (m, 3H), 7.33 (ddd, J = 8.0, 6.8, 1.1 Hz, 1H), 7.14 (d, J = 8.9 Hz, 1H), 2.34 (s, 3H) ppm.

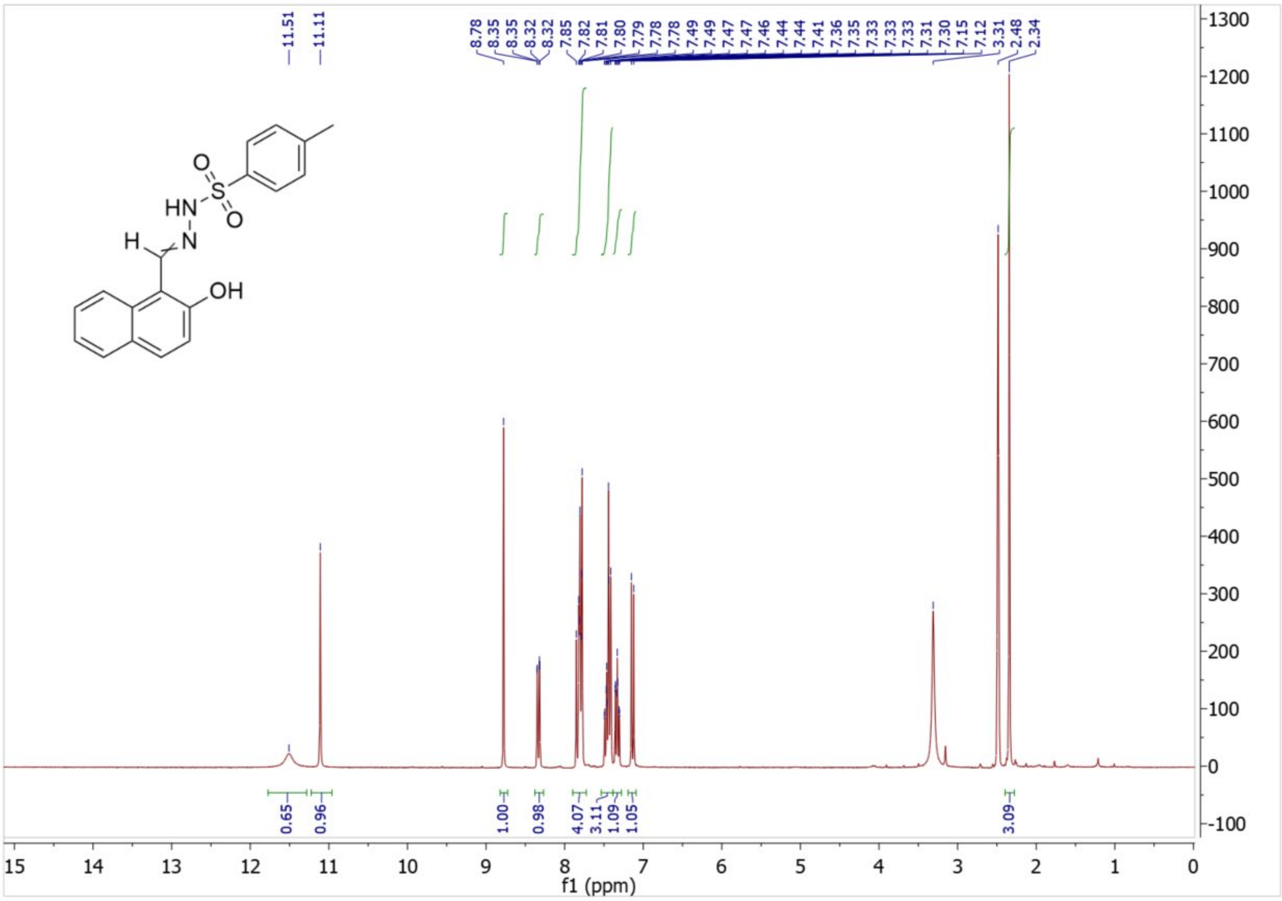

N'-(2-((1-benzylpiperidin-4-yl)methyl)-5,6-dimethoxy-2,3-dihydro-1H-inden-1-ylidene)-4-methylbenzenesulfonohydrazide (**12**)

In a round bottom flask 4-methylbenzenesulfonohydrazide (0.09 g; 0.5 mmol) and donepezil (0.19 g; 0.5 mmol) were dissolved in 10 mL ethanol in a pressure tube. The resulting mixture was stirred at 120 °C for 5 hours. After completion of the reaction the solvent was evaporated. The crude was purified by normal-phase flash chromatography.

Yield: 0.21 g (38%); white solid; m.p.: 105 °C; ^{1}H NMR (300 MHz, DMSO-d_6) δ 7.83 – 7.67 (m, 4H), 7.66 – 7.57 (m, 2H), 7.46 – 7.38 (m, 2H), 7.33 (d, J = 8.0 Hz, 2H), 7.22 – 7.10 (m, 2H), 5.23 (s, 1H), 3.04 – 2.64 (m, 6H), 2.45 – 2.26 (m, 7H), 1.91 – 1.76 (m, 2H), 1.65 (d, J = 13.5 Hz, 2H) ppm; ^{13}C NMR (75 MHz, DMSO-d_6) δ 166.25 , 152.54 , 149.22 , 143.53 , 140.89 , 136.57 , 129.71 , 129.30 , 129.02 , 128.60 , 128.17 , 127.43 , 108.52 , 103.47 , 56.01 (d, J = 2.4 Hz), 53.48 , 37.67 , 35.02 , 33.98 , 21.43 ppm. HRMS (ESI/Q-TOF) m/z: $[M+H]^+$ Calcd. for $C_{31}H_{38}N_3O_4S$ 548.2583; found 548.2575.

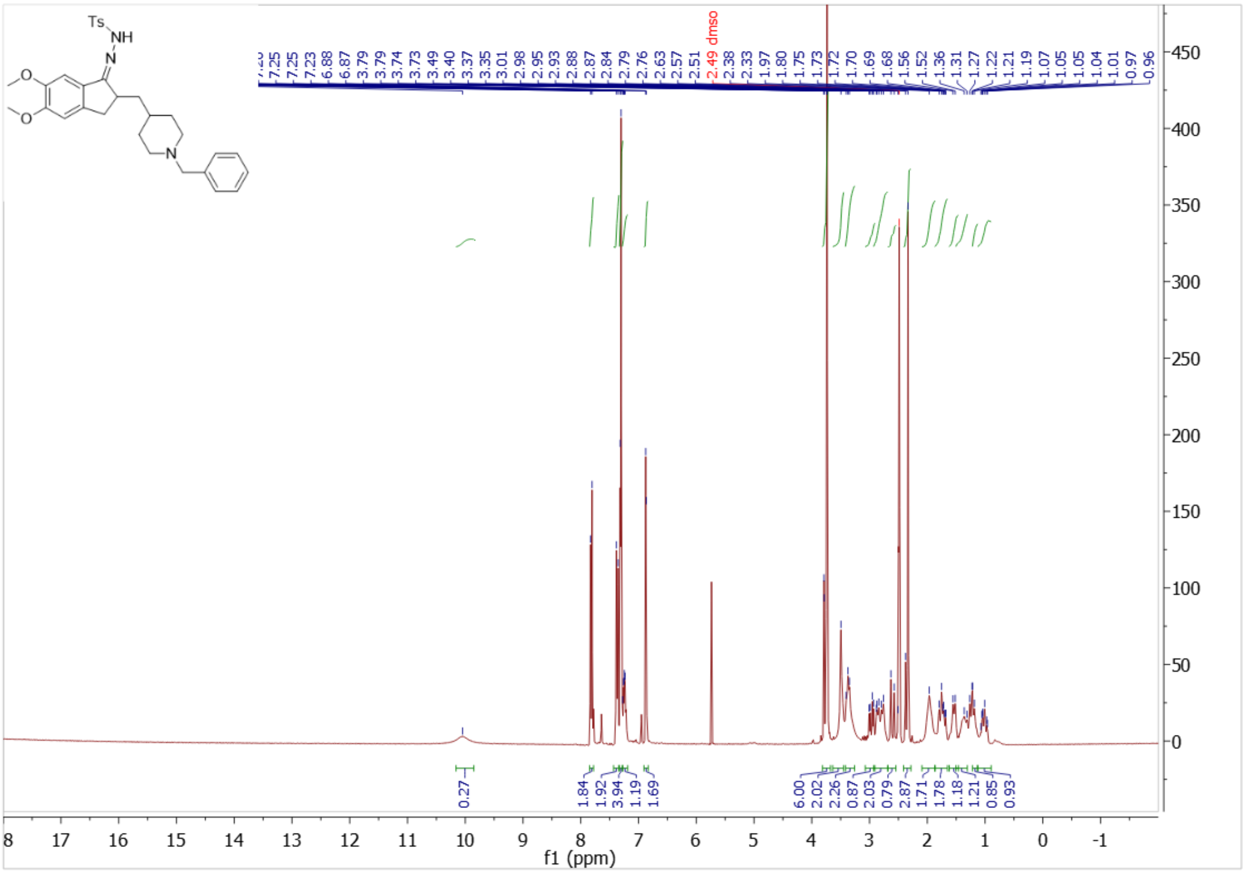

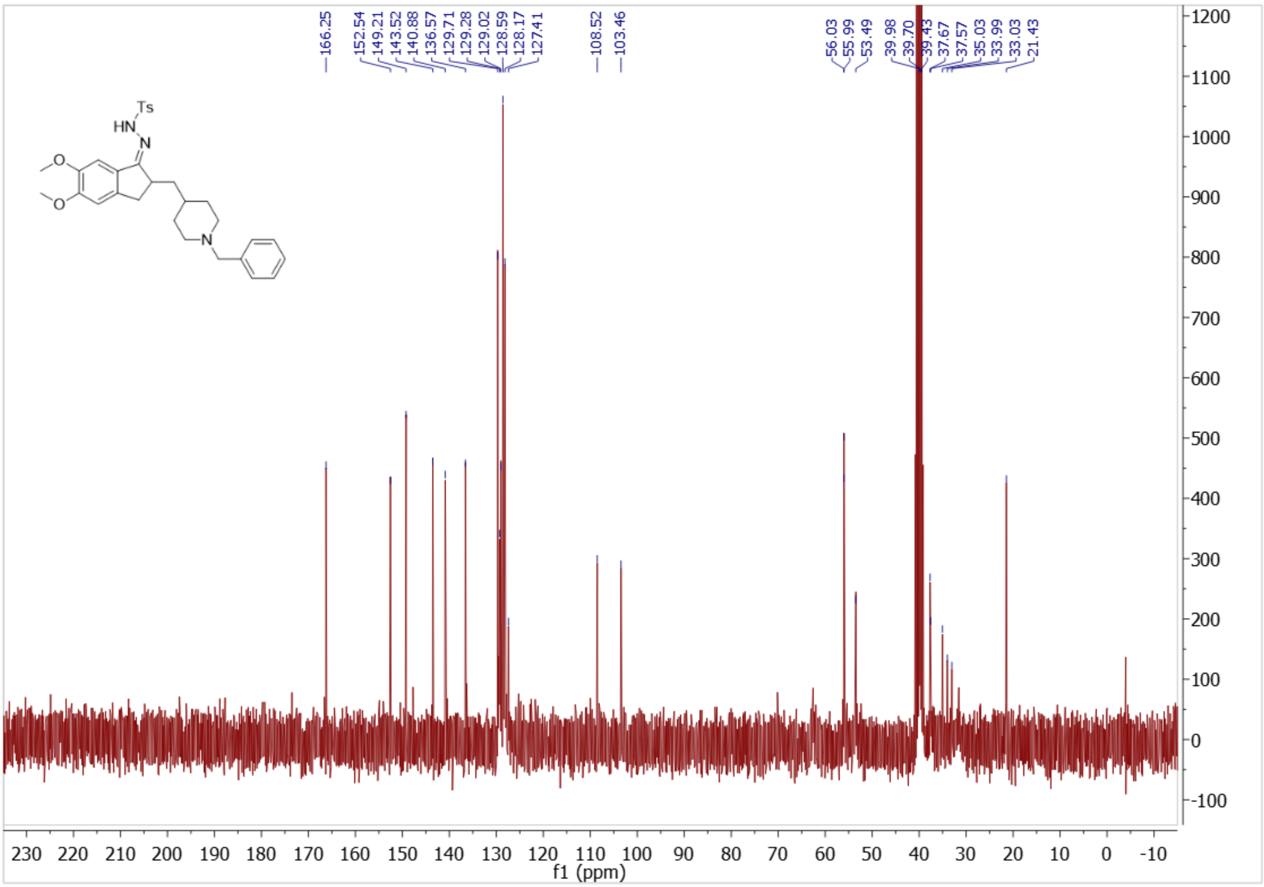

Synthesis of *N*-(19-azido-7-oxo-11,14,17-trioxa-3,4-dithia-8-azanonadecyl)-5-((3a*S*,4*S*,6a*R*)-2-oxohexahydro-1*H*-thieno[3,4-d]imidazol-4-yl)pentanamide (azido-PEG3-SS-biotin)

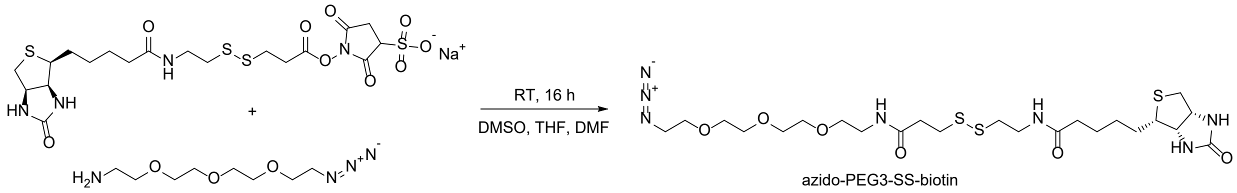

NHS-SS-biotin (10 mg, 0.017 mmol, https://www.thermofisher.com/order/catalog/product/PG82077) was dissolved in 0.5 mL DMSO. In 1 mL THF azido-PEG3-amine (3.7 mg, 0.017 mmol, https://broadpharm.com/product/bp-20580) was added. White precipitate formed that was dissolved slowly after adding 1 mL DMF. The reaction mixture was stirred at room temperature for 8 h than kept in fridge overnight. The reaction was followed by LCMS that confirmed full conversion after 24 h. The product was isolated by preparative HPLC.

Yield: 5 mg (50%), yellowish solid. ^{1}H NMR (500 MHz, DMSO-*d*$_6$) δ 8.52 (s, 1H), 8.06 – 7.98 (m, 2H), 6.37 (d, *J* = 26.4 Hz, 2H), 4.31 (s, 1H), 4.14 (s, 1H), 3.64 – 3.59 (m, 2H), 3.54 (dd, *J* = 14.0, 4.2 Hz, 8H), 3.40 (dq, *J* = 14.1, 7.5 Hz, 5H), 3.21 (q, *J* = 6.0 Hz, 2H), 3.14 – 3.07 (m, 1H), 2.90 (t, *J* = 7.1 Hz, 2H), 2.83 (dd, *J* = 12.4, 4.9 Hz, 1H), 2.77 (t, *J* = 6.6 Hz, 2H), 2.59 (d, *J* = 12.4 Hz, 1H), 2.50 – 2.45 (m, 2H), 2.08 (t, *J* = 7.2 Hz, 2H), 1.67 – 1.19 (m, 7H) ppm. ^{13}C NMR (126 MHz, DMSO-*d*$_6$) δ 203.27, 172.14, 170.05, 162.64, 69.74, 69.71, 69.64, 69.52, 69.18, 69.00, 60.98, 59.15, 55.33, 49.96, 38.56, 37.83, 37.31, 35.08, 34.95, 34.00, 28.10, 27.96, 25.15 ppm. HRMS (ESI/Q-TOF) m/z: [M+H]$^+$ Calcd. for $C_{23}H_{42}N_7O_6S_3$ 608.2280; found 608.2358.

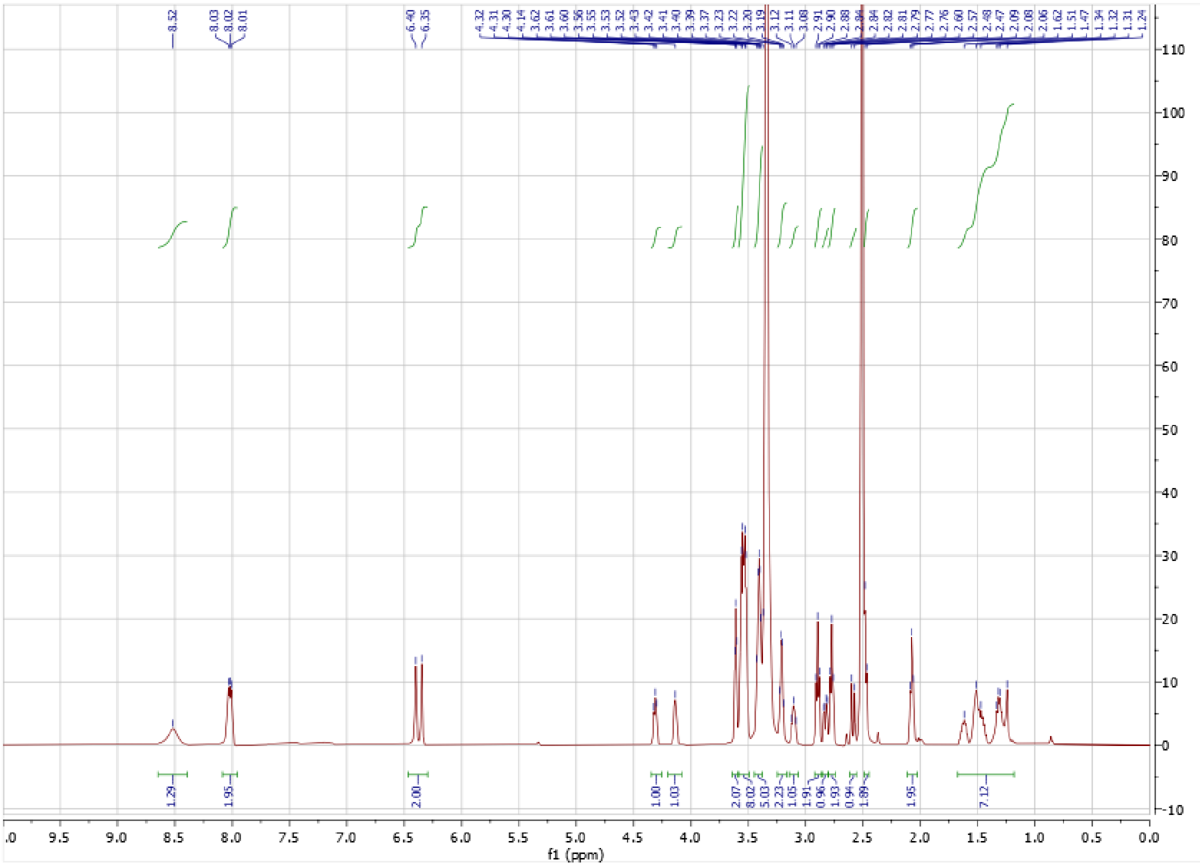

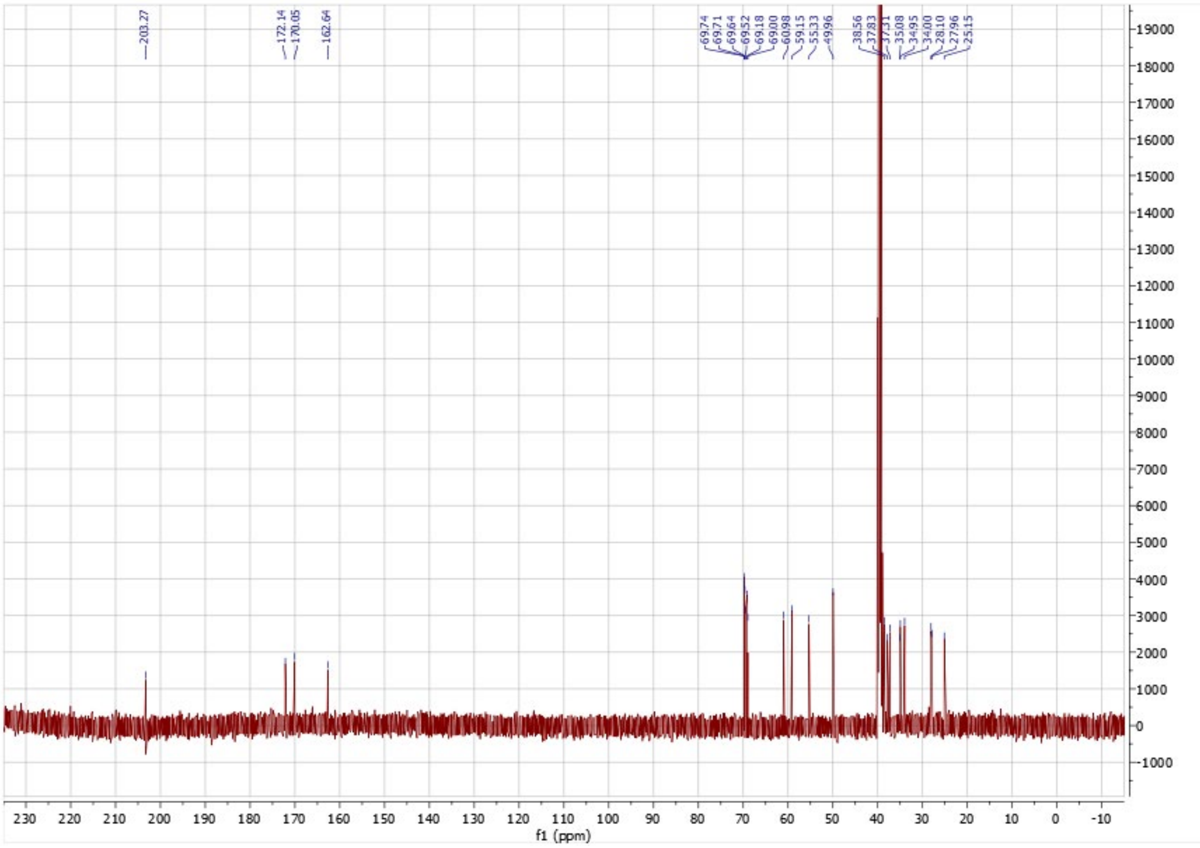

Supplement: Supplementary file 1 — Supporting Information [file ANIE-64-e202408701-s002.pdf]
